# Gasdermin D mediates endoplasmic reticulum stress via FAM134B to regulate cardiomyocyte autophagy and apoptosis in doxorubicin-induced cardiotoxicity

**DOI:** 10.1038/s41419-022-05333-3

**Published:** 2022-10-26

**Authors:** Ya’nan Qu, Rifeng Gao, Xiang Wei, Xiaolei Sun, Kun Yang, Huairui Shi, Yang Gao, Shiyu Hu, Yiwen Wang, Ji’e Yang, Aijun Sun, Feng Zhang, Junbo Ge

**Affiliations:** 1 Department of Cardiology, Shanghai Institute of Cardiovascular Diseases, Zhongshan Hospital, Fudan University, 200032 Shanghai, China; 2 Department of Cardiology, Shanghai Fifth People’s Hospital, Fudan University, 200240 Shanghai, China; 3 Key Laboratory of Viral Heart Diseases, National Health Commission, 200032 Shanghai, China; 4 Key Laboratory of Viral Heart Diseases, Chinese Academy of Medical Sciences, 200032 Shanghai, China; 5 National Clinical Research Center for Interventional Medicine, 200032 Shanghai, China; 6 Institutes of Biomedical Sciences, Fudan University, 200032 Shanghai, China

**Keywords:** Apoptosis, Autophagy, Cardiovascular diseases

## Abstract

Cardiomyocyte pyroptosis and apoptosis play a vital role in the pathophysiology of several cardiovascular diseases. Our recent study revealed that gasdermin D (GSDMD) can promote myocardial I/R injury via the caspase-11/GSDMD pathway. We also found that GSDMD deletion attenuated myocardial I/R and MI injury by reducing cardiomyocyte apoptosis and pyroptosis. However, how GSDMD mediates cardiomyocyte apoptosis and protects myocardial function remains unclear. Here, we found that doxorubicin (DOX) treatment resulted in increased apoptosis and pyroptosis in cardiomyocytes and that caspase-11/GSDMD could mediate DOX-induced cardiotoxicity (DIC) injury. Interestingly, GSDMD overexpression promoted cardiomyocyte apoptosis, which was attenuated by GSDMD knockdown. Notably, GSDMD overexpression exacerbated DIC injury, impaired cardiac function in vitro and in vivo, and enhanced DOX-induced cardiomyocyte autophagy. Mechanistically, GSDMD regulated the activity of FAM134B, an endoplasmic reticulum autophagy receptor, by pore formation on the endoplasmic reticulum membrane via its N-terminus, thus activating endoplasmic reticulum stress. In turn, FAM134B interacted with autophagic protein LC3, thus inducing cardiac autophagy, promoting cardiomyocyte apoptosis, and aggravating DIC. These results suggest that GSDMD promotes autophagy and induces cardiomyocyte apoptosis by modulating the reaction of FAM134B and LC3, thereby promoting DIC injury. Targeted regulation of GSDMD may be a new target for the prevention and treatment of DIC.

## Introduction

Pyroptosis is a novel and cryptic form of programmed cell death, identified as cell swelling until membrane rupture, leading to the release of cell contents and triggering a pronounced inflammatory response [1, 2]. Gasdermin D (GSDMD) is a protein closely related to pyroptosis [1–4]. Some studies have reported that GSDMD-mediated pyroptosis inhibits apoptosis [5–7], while others have reported that it is irrelevant to apoptosis [8, 9]. Hence, the type of cell death mediated by GSDMD remains controversial.

Pyroptosis and apoptosis occur in myocardial ischemia reperfusion (I/R) and myocardial infarction (MI), and GSDMD plays an important role in I/R, MI, and atherosclerosis [10–12]. Specifically, GSDMD inhibition reduces cardiomyocyte pyroptosis in I/R and MI as well as apoptosis. Further, cardiomyocyte pyroptosis is regulated by the caspase-11/GSDMD pathway under oxidative stress conditions [12]. However, whether GSDMD is involved in cardiomyocyte apoptosis remains unclear, as is its role in doxorubicin (DOX)-induced cardiotoxicity (DIC).

DOX is used extensively in a broad range of malignancies in children and adults [13]. Despite its wide clinical application, DOX treatment is largely restricted by its accumulative cardiotoxicity. The incidence of cardiomyopathy and the subsequent heart failure is high when the cumulative DOX dose used exceeds 550 mg per square meter of body-surface area [14]. However, no definitive evidence for the specific underlying mechanism between DOX and cardiomyocyte pyroptosis has been revealed.

DOX reportedly affects cardiomyocyte autophagic flux and the autophagosome to autolysosome ratio [15–17]. Autophagy is a conserved process of degradation and recovery of cytoplasmic components. It is regarded as a protective mechanism that maintains cellular homeostasis. However, dysregulation of autophagy may cause cardiac dysfunction and even lead to irreversible degenerative cardiomyopathy and congestive heart failure [18]. Autophagy is initiated at the omegasome, a cup structure on the endoplasmic reticulum (ER) [19, 20]. Further, some stimuli that induce ER stress (ERS) also cause autophagy. Degradation of specific ER components, lipid membranes, and large protein aggregates in or at the ER occurs during ER-phagy [21]. The selective destruction is driven by the recognition of autophagy receptors and their LC3-interacting region (LIR) domains [22]. ERS triggers non-selective macroautophagy and also directly induces ER-phagy [23]. Hence, there is considerable interest in understanding the role of ER in DIC.

Herein, we investigated the role of GSDMD in DIC and the underlying molecular mechanisms, with an aim to identify novel targets for the prevention and treatment of DIC.

## Materials and methods

### Method overview

Molecular signaling events in cardiac tissue and cardiomyocytes were determined by immunoblotting, immunostaining, and RNA sequencing. Cardiac function and autophagy flow were investigated by echocardiography and mRFP-GFP-LC3 probing, respectively. Detailed experimental descriptions are provided in the Supplementary Methods.

### Animals

All animal experiments were performed under specific sterile barrier conditions in accordance with institutional guidelines, and the experimental protocols were approved by the Ethics Committee of Animal Experimentation of Fudan University. Detailed descriptions of these experiments are provided in the Supplementary Methods.

### Statistical analysis

Statistical analyses were conducted using GraphPad Prism 8.0 software (GraphPad Software, San Diego, CA). *P* < 0.05 was considered statistically significant. All experiments were performed at least three times. Details of statistical analysis are provided in the Supplementary Methods.

## Results

### GSDMD is essential for myocardial I/R injury and myocardial infarction

Myocardial I/R and MI are associated with pyroptosis and apoptosis [10, 12, 24]. We confirmed in I/R and MI model mice that the expression of pyroptosis-related proteins and apoptosis-related proteins was markedly increased but decreased after GSDMD knockout (GSDMD-KO) (Fig. S1A–D). Echocardiography verified that GSDMD loss reduced the impairment of cardiac function in I/R or MI mice (Fig. S1E–S1H). Importantly, GSDMD deficiency decreased serum lactate dehydrogenase (LDH) release (Fig. S1I), oxidative stress [reactive oxygen species (ROS) levels; Fig. S1J)], and the number of terminal deoxynucleotidyl transferase dUTP nick-end labeling (TUNEL)-positive cardiomyocytes (Fig. S1K). Taken together, these observations indicated that GSDMD-related pyroptosis and apoptosis play an important role in I/R and MI.

### DIC injury in cardiomyocytes mediated by the Caspase 11-GSDMD-N pathway

The role of GSDMD-mediated pyroptosis and apoptosis in I/R and MI (Fig. S1) indicated that GSDMD participated in myocardial injury. DIC is also a kind of myocardial injury. To investigate the effect of DOX on cardiomyocytes in vivo, WT mice were injected intraperitoneally with DOX to induce acute cardiotoxicity. Sham mice were injected with normal saline (NS), as a control group. Samples were collected 7 d after DOX treatment. The survival rate, heart weight, and body weight (BW) of DOX-treated mice were lower than those of sham mice (Fig. S2A and S2B). Consistently, DOX administration significantly impaired cardiac function (Fig. S2C and S2D) and triggered an evident cardiomyocyte contractile dysfunction (Fig. S2E and S2F), detected as reduced maximal velocity of shortening/re-lengthening (+dL/dt), peak shortening (PS), and time to peak shortening (TPS) (all at *P* < 0.001) without affecting [12] time to 90% re-lengthening (TR90). Moreover, DOX-induced myocardial injury obviously upregulated the expression of cleaved caspase-3 (CC3) and BAX and decreased BCL-2 levels (Fig. S2G and S2H).

We used wheat germ agglutinin (WGA) to visualize cardiomyocyte diameter. Further, since DOX is thought to act as an electron acceptor to generate ROS, we also assessed ROS levels in mouse tissues. WGA staining revealed cardiomyocyte diameter reduction after DOX administration, and ROS staining suggested more oxidative stress, as indicated by high fluorescence intensity of dihydroethidium (DHE) in cardiac tissues of the DOX-treated group (Fig. S2I). We also used TUNEL assay to analyze left ventricular tissue sections 7 d post DOX administration. Compared with those in the sham control, the numbers of TUNEL-positive cells were increased in DOX-treated mice (Fig. S2J and S2L).

The above results indicated that the DIC model was successfully constructed, and DOX administration triggered adverse effects in cardiomyocytes. Finally, we probed GSDMD involvement in DIC, finding that the levels of full-length GSDMD and N-terminus of GSDMD were increased in the DOX-treated group (Fig. S2K). Collectively, these observations confirmed that GSDMD plays a role in DIC.

Caspase-11 reportedly cleaved GSDMD and induced upregulation of GSDMD-N expression in myocardial I/R injury [12]. Meanwhile, caspase-1 and caspase-3 may also be involved in the activation of GSDMD and GSDME [25, 26]. To explore the molecular mechanism of DOX-induced activation of GSDMD, we used adenovirus to knock down caspase-11, caspase-1, and caspase-3 in adult mouse cardiomyocytes. We found that GSDMD expression did not decrease when caspase-1 and caspase-3 were knocked down in the DOX-treated group, but only when caspase-11 was knocked down, indicating that caspase-11 regulated GSDMD as its upstream molecule (Fig. S3A–S3C). Taken together, we considered that the caspase-11-GSDMD pathway mediated DIC injury in cardiomyocytes.

### GSDMD plays a role in DIC by mediating cardiomyocyte apoptosis

To investigate whether GSDMD plays a role in DIC by inducing cardiomyocyte pyroptosis, we examined the expression of GSDMD, lactate dehydrogenase (LDH) release, and interleukin (IL)-18 levels in adult mouse cardiomyocytes supernatants treated with 1 μM DOX at different time points. Western blot showed that the level of GSDMD-N increased significantly at 3 h and gradually increased with the prolongation of DOX treatment time (Fig. S4A and S4B). The same changes were observed for apoptosis-related proteins (Fig. S4A). Surprisingly, LDH release and IL-18 levels increased after 3 h of DOX treatment (Fig. S4C and S4D). However, the concentration of IL-18 decreased to normal level around 12 h, while LDH continued to increase. Furthermore, a 24 h-consecutive microscopic imaging of cell death showed that a large number of cardiomyocytes gradually shrunk and died in an apoptotic manner after DOX treatment (Fig. S5A and S5B). At the same time, we used confocal microscopy to observe the dynamic changes in cardiomyocytes from adult mice treated with DOX for 12 h and captured continuous dynamic images of multiple cardiomyocyte death. We found that adult mouse cardiomyocytes gradually shrunk with intact membrane integrity and chromatin pyknosis after DOX treatment (Fig. S6A and S6B). These results indicated that cardiomyocyte pyroptosis could be induced in the early stage of DOX treatment, and a large number of adult mouse cardiomyocytes died via apoptosis with the prolongation of DOX treatment time. Therefore, to explore whether GSDMD plays a role in DIC by mediating cardiomyocyte apoptosis, we selected 12 h of DOX treatment as the time point for the subsequent study.

### GSDMD deficiency alleviates DIC and GSDMD overexpression aggravates DIC

**Fig. 1:**
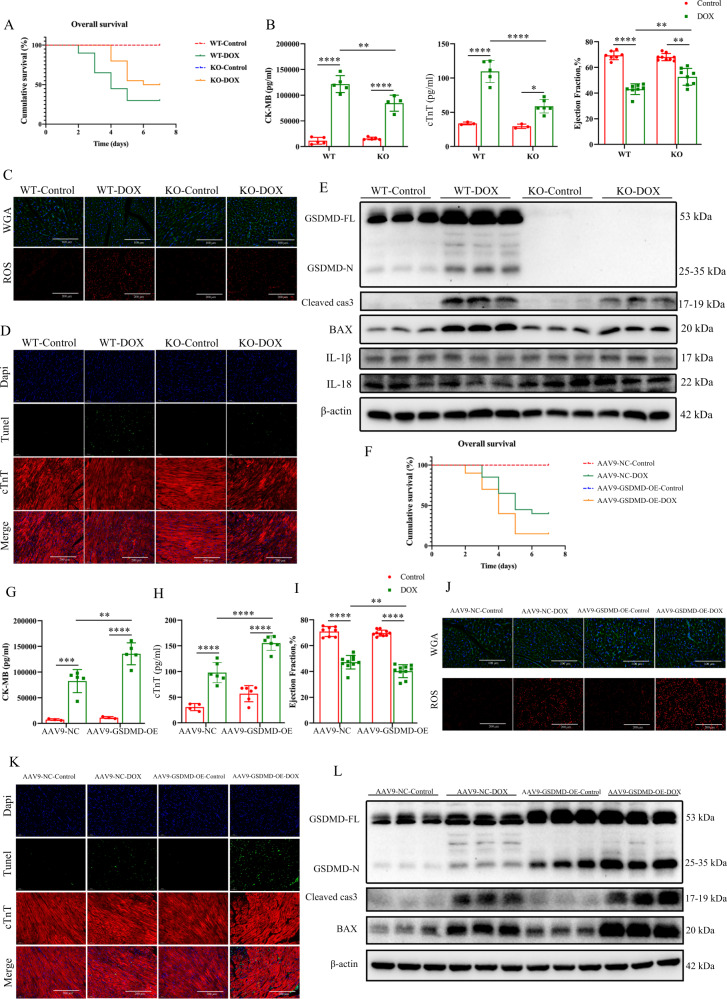
Deficiency or overexpression of GSDMD alleviates or aggrevates DOX-induced cardiotoxic injury. **A** Kaplan–Meier test showing the overall survival rates of WT and GSDMD-KO mice treated with saline or DOX. *N* = 10 for Control groups and *n* = 20 for DOX groups. **B** Serum of CK-MB and cTnT release detected by Elisa. *N* = 3–6 per group. Ejection fraction (EF) of four different groups measured by echocardiography. *N* = 7–9 per group. **C** Epifluorescence microscopy of WGA and ROS. *N* = 10–11 per group. **D** TUNEL assay in myocardium treated with vehicle or DOX co-staining with cTnT (scale bar: 200 μm). *N* ≥ 6 per group. **E** Western blots of GSDMD-FL, GSDMD-N, Cleaved-caspase 3 (Cleaved cas3), BAX, IL-18, IL-1β, and β-actin proteins from vehicle-treated control and DOX-treated hearts in the absence and presence of GSDMD. *N* = 6 per group. **F** Survival rates of DOX-treated mice and respective sham mice in adeno-associated virus serotype 9 (AAV9-GSDMD-OE) mice or negative control respectively. *N* = 10 for Control groups and *n* = 20 for DOX groups. **G**, **H** Serum concentrations of CK-MB and cTnT, respectively. *N* = 3–6 per group. **I** Ejection fraction (EF) measured by echocardiography. *N* = 8–10 per group. **J** WGA (scale bar: 100 μm) and ROS staining (scale bar: 200 μm) detected by epifluorescence microscopy. *N* = 10 for WGA groups and *n* = 11 for ROS groups. **K** Apotosis of myocardium by TUNEL staining co-stained with cTnT (scale bar: 200 μm). *N* = 10 per group. **L** Detection of GSDMD, Cleaved-caspase 3 (Cleaved cas3), BAX, and β-actin proteins levels by western blot. *N* = 6 mice per group. Data are depicted as the mean ± SEM. Statistical significance was determined by one-way and two-way ANOVA with a post hoc Holm–Sidak test, ns, not significant; *P < 0.05; **P < 0.01; ***P < 0.001; ****P < 0.0001.

To explore the biological function of GSDMD in acute animal models of DIC, we established GSDMD-deficient mice. DOX injection led to a high mortality among WT mice, and GSDMD-KO mice further declined (Fig. 1A). Further, the reduction in body weight was lower in DOX-treated KO mice than in WT mice, and DOX-treated KO mice exhibited significantly increased ratios of heart weight (Fig. S7A and S7B). DOX treatment-induced increase in serum creatine kinase-MB (CK-MB) levels and cardiac troponin T (cTnT) activity was further enhanced in WT mice compared with levels in GSDMD-KO mice (Fig. 1B). Further, cardiac function reduction upon DOX injection was significantly improved in GSDMD-KO mice, with an increased ejection fraction (EF) and fractional shortening (FS) in DOX-injected GSDMD-KO mice (Fig. 1B and Fig S7C–S7E). Decreased +dL/dt max and -dL/dt min represent systolic dysfunction and diastolic dysfunction, respectively [27], and we observed that DOX exacerbated cardiac function in WT mice but the effect was not pronounced in KO mice (Fig. S7G and S7H). Other contractile indexes displayed a similar tendency (Fig. S7F and S7H). Consistently, WGA staining revealed that cardiomyocyte diameter decreased upon DOX treatment, with GSDMD deficiency suppressing the DIC-induced shrinkage (Fig. 1C and Fig. S7I). Interestingly, LDH levels in adult mouse cardiomyocytes treated with DOX for 12 h did not differ significantly between WT and KO groups, in addition to the inapparent levels of IL-18 (Fig. S4E). Collectively, these observations demonstrate that GSDMD downregulation mitigates cell death after DOX administration.

Next, we infected adult mice with an adenovirus-associated serotype 9 GSDMD-overexpression construct (AAV9-GSDMD-OE) or an adenovirus-associated normal control construct (AAV-NC) to further verify the role of GSDMD in DOX cardiotoxicity. DOX treatment resulted in increased mortality, cardiac injury, as determined by serum biomarker analysis, and cardiac dysfunction of AAV9-GSDMD-OE mice compared with those in WT mice (Fig. 1G–I, and Fig. S7P, S8C, and S8D). Furthermore, ROS levels in DOX-treated GSDMD-overexpressing mice were enhanced compared with those in the control (Figs. 1J and S8H). Compared with that in the control, the number of TUNEL-positive cardiomyocytes in GSDMD-KO mice treated with DOX was reduced (Figs. 1D and S7M); however, this effect was reversed by AAV9-GSDMD-OE infection (Figs. 1K and S8K). Further, the levels of apoptosis-related proteins BAX and CC3 were significantly increased in DOX treatment groups, and GSDMD loss attenuated this effect (Fig. 1E and Fig. S7K and S7L). The decrease in protein levels caused by DOX in GSDMD-KO mice was reversed by GSDMD overexpression (Fig. 1L and Fig. S8I and S8J). In summary, these observations indicated GSDMD involvement in DOX-related cardiomyopathy.

Interestingly, GSDMD deficiency alleviated cardiomyocyte apoptosis, whereas GSDMD overexpression aggravated cardiomyocyte apoptosis. Taken together, these results demonstrate that GSDMD-mediated cardiomyocyte apoptosis plays an important role in DIC.

### Cardiomyocyte-specific GSDMD deficiency alleviates acute and chronic DIC

**Fig. 2:**
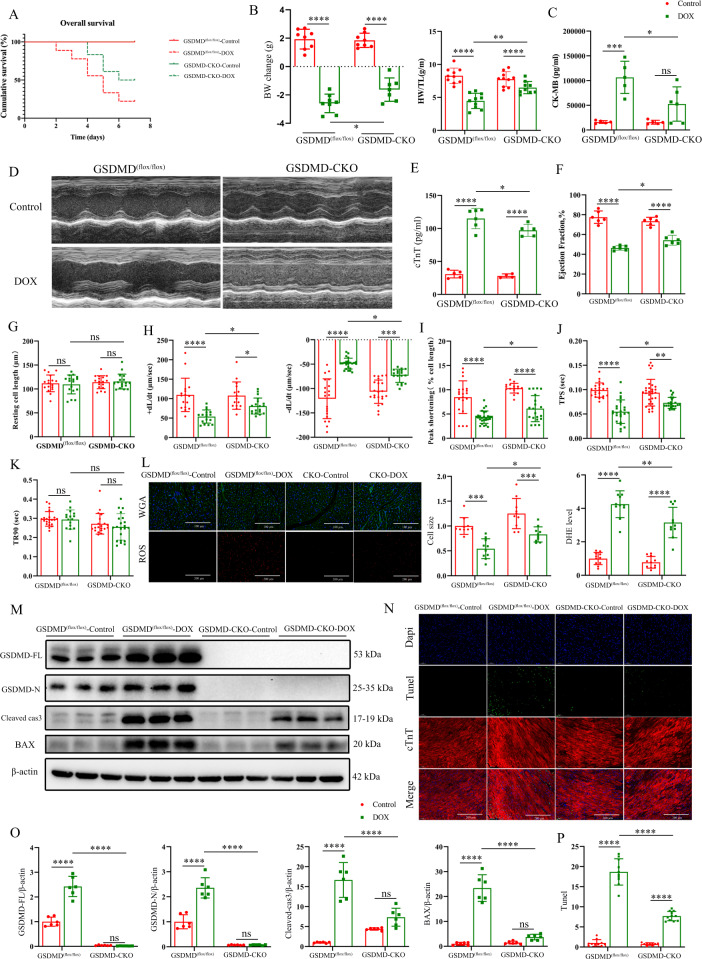
Cardiomyocyte-specific GSDMD deficiency alleviates acute and chronic DIC. **A** Cumulative survival rates of GSDMD^(flox/flox)^ mice and GSDMD-CKO mice treated with saline or DOX. *N* ≥ 10. **B** Body weight change (g) and ratio of heart weight to tibial length (HW/TL) in GSDMD^(flox/flox)^ and GSDMD-CKO mice with or without DOX treatment. *N* = 7–10 per group. **C** Changes of serum cardiac biomarkers (CK-MB) after DOX intervention. *N* = 5–6 per group. **D** Representative echocardiographic images in different groups. *N* = 6 per group. **E** Changes of serum cardiac biomarkers (cTnT) after DOX intervention. *N* = 4–6 per group. **F** Ejection fraction (EF) measured by echocardiography. *N* = 6 per group. **G**–**K** Effect of DOX on hemodynamic measurements. **L** Cardiomyocyte size and ROS level in the indicated groups (scale bar: 100 μm for WGA and 200 μm for ROS). *N* = 10 for WGA groups and n = 11 for ROS groups. **M**, **O** Representative western blots and analysis of GSDMD (FL and N teminals), cleaved-caspase 3 (Cleaved cas3), BAX, and β-actin proteins levels in GSDMD^(flox/flox)^ and GSDMD-CKO hearts. *N* = 6 per group. **N**, **P** TUNEL and cTnT staining and analysis in DOX-treated hearts of GSDMD^(flox/flox)^ and GSDMD-CKO mice (scale bar: 200 μm). *N* = 9–11 per group. Data are depicted as the mean ± SEM. Statistical significance was determined by one-way and two-way ANOVA with a post hoc Holm–Sidak test, ns, not significant; *P < 0.05; **P < 0.01; ***P < 0.001; ****P < 0.0001.

Figure 1 suggests that GSDMD plays a vital role in regulating the DOX-associated myocardial injury. GSDMD reportedly induces cell death [1, 3]. However, whether the same is true in the cardiac context remains unknown. Accordingly, to investigate this, we established mice with cardiomyocyte-specific GSDMD KO (GSDMD-CKO). GSDMD-CKO totally abolished the expression of GSDMD protein (Fig. 2M). Cardiac GSDMD deficiency decreased the impairment of DOX-induced myocardial injury. After DOX intervention, compared with the control group, the survival rate of GSDMD-CKO mice was significantly prolonged, BW and heart weight loss were decreased, and levels of the myocardial injury markers CK-MB and cTnT were remarkably decreased (Fig. 2A–C, E). The damage of cardiac function in DOX-treated GSDMD-CKO mice was reduced, with a greater increase in ejection fraction, fractional shortening, and contractile indexes than those in control mice (Fig. 2D, F–K and Fig. S9A). In addition, following DOX treatment, cardiomyocytes in GSDMD-CKO mice were bigger than those in GSDMD^(flox/flox)^ mice (Fig. 2L). ROS staining based on DHE fluorescence revealed that ROS levels in DOX-treated GSDMD-CKO mice were lower than those in GSDMD^(flox/flox)^ littermates (Fig. 2L). Cardiomyocyte apoptosis after DOX administration in GSDMD-CKO mice was reduced compared to that in GSDMD^(flox/flox)^ mice, as indicated by decreased CC3 and BAX protein levels (Fig. 2M, O) and the number of TUNEL-positive cells (Fig. 2N, P).

We next repeated the above experiments in a 6-week model of chronic DOX cardiotoxicity. Consistent with the observations in the acute model, DOX-treated GSDMD-CKO mice had an improved survival rate, and with higher heart weight and BW at the end of the observation period than those in control mice (Fig. S9B–S9D). Echocardiography at weeks 2 and 4 did not reveal any significant differences between DOX-treated GSDMD^(flox/flox)^ and GSDMD-CKO mice; however, cardiac function notably improved by week 6 in CKO mice compared with that in GSDMD^(flox/flox)^ DOX-treated controls (Fig. S9E and S9F). Western blotting revealed a remarkable reduction in the levels of apoptosis-related proteins in CKO heart after DOX treatment (Fig. S9G and S9H). Collectively, these observations indicated that GSDMD KO in the heart is protective against DIC in both acute and chronic models of DOX cardiomyopathy.

### GSDMD deficiency leads to cardiomyocyte apoptosis and exacerbates DIC

**Fig. 3:**
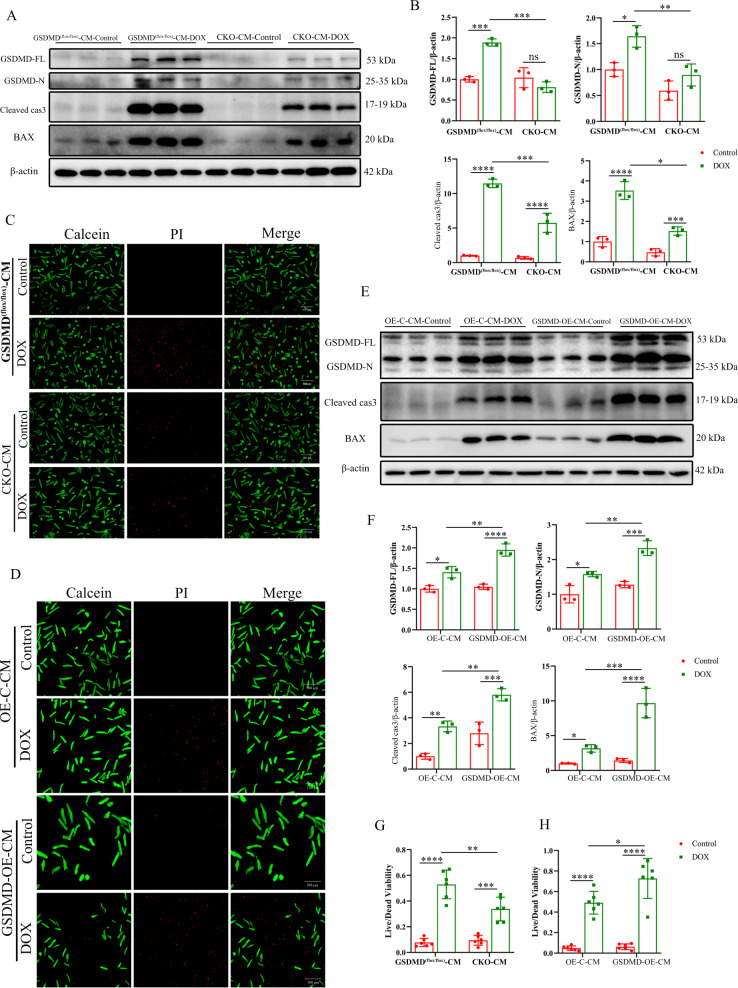
GSDMD deficiency causes apoptosis in cardiomyocytes and exacerbates DIC. **A**, **B** GSDMD-FL, GSDMD-N, Cleaved-caspase 3 (Cleaved cas3) and BAX were measured using western blot assay, and relative band density was quantified. *N* = 3 per group. **C**, **G** Cell viability was determined using PI staining, and the live-to-dead cell ratios of GSDMD^(flox/flox)^ and GSDMD-CKO cardiomyocytes were quantitated (scale bar: 100 μm). *N* = 6 per group. **E**, **F** The protein levels of GSDMD-FL, GSDMD-N, cleaved-caspase 3 (Cleaved cas3), and BAX in OE-C and GSDMD-OE cardiomyocytes treated with saline or DOX. *N* = 3 per group. **D**, **H** Cell viability was determined using PI staining, and the live-to-dead cell ratios of OE-C and GSDMD-OE cardiomyocytes were quantitated (scale bar: 100 μm). *N* = 6 per group. Data are depicted as the mean ± SEM. Statistical significance was determined by one-way and two-way ANOVA with a post hoc Holm–Sidak test, ns, not significant; *P < 0.05; **P < 0.01; ***P < 0.001; ****P < 0.0001.

Similar effects of GSDMD deletion on DOX treatment-induced damage of cardiomyocytes were accordantly observed in vivo. GSDMD KO in cardiomyocytes reduced DOX-induced increase in GSDMD N-terminus, CC3, and BAX protein levels (Fig. 3A, B) and the number of dead cardiomyocytes, as assessed by propidium iodide staining (Fig. 3C, G). All these effects were reversed by adenovirus-mediated GSDMD overexpression (Fig. 3D–G). Collectively, these observations confirmed that GSDMD causes apoptosis in cardiomyocytes, thus exacerbating DIC.

### GSDMD promotes DOX-induced myocardial autophagy

**Fig. 4:**
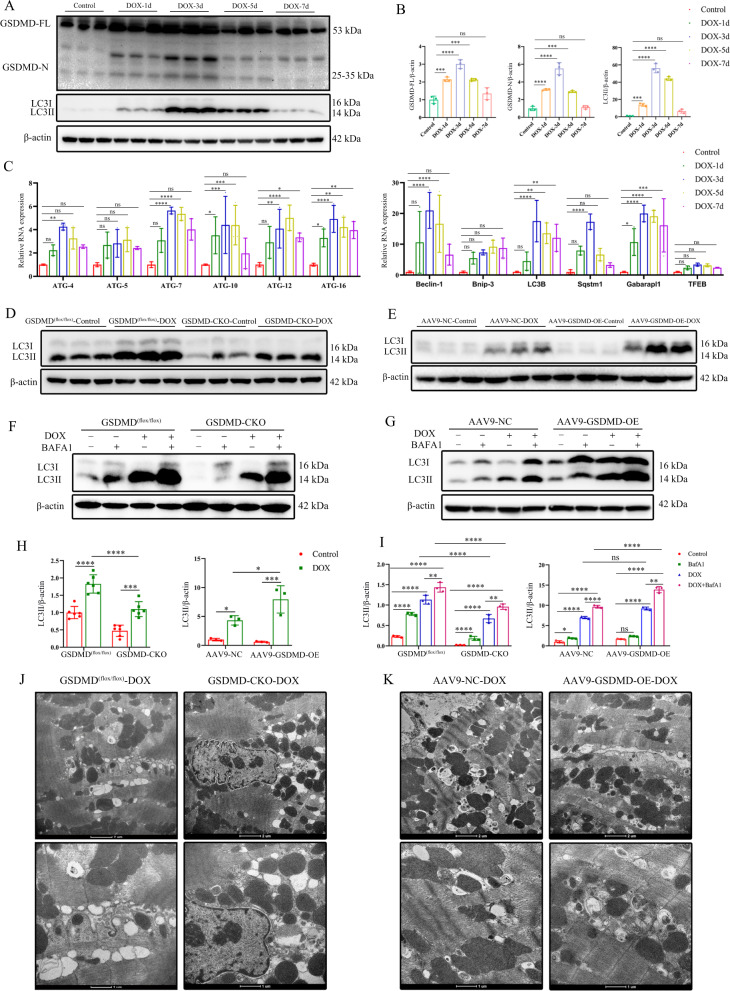
DOX activates cardiac autophagy, and GSDMD promotes myocardial autophagy levels. **A**, **B** Temporal changes in GSDMD (FL and N teminals), LC3II (microtubule-associated protein 1A/1B-light chain 3) protein levels. *N* = 3 per group. **C** PCR analysis of autophagy-related-genes (Atg4, Atg5, Atg7, Atg10, Atg12, Atg16, Beclin-1, Bnip-3, LC3B, Sqstm1, Gabarapl1, and TFEB) in mouse hearts at 0, 1, 3, 5, and 7 days after DOX injection. *N* = 3 per group. **D**, **E** Representative western blots of LC3 in DOX-induced GSDMD^(flox/flox)^ and GSDMD-CKO hearts or AAV9-NC and AAV9-GSDMD-OE hearts. *N* = 6 for former groups and *n* = 3 for later groups. **F**, **G** Representative western blots of LC3 in DOX-induced GSDMD^(flox/flox)^ and GSDMD-CKO hearts or AAV9-NC and AAV9- GSDMD-OE hearts with or without BAFA1. *N* = 3 per group. BAFA1 treatment, 2.5 mg/kg, intraperitoneal injection 2 h before the surgery. **H** Analysis of LC3II protein expression in GSDMD^(flox/flox)^ and GSDMD-CKO or AAV9-NC and AAV9-GSDMD-OE hearts in acute DOX model. *N* = 6 for GSDMD^(flox/flox)^ and GSDMD-CKO mice group, *n* = 3 for AAV9-NC and AAV9-GSDMD-OE groups. **I** Statistics of LC3II protein expression in different mice groups treated with DOX and/or BafA1. *N* = 3 per group. **J**, **K** Representative electron micrograph images of murine cardiac muscle derived from mice treated with DOX. Magnified section showing ultrastructural defects including autophagosomes and autolysosomes. (scale bar: 2 μm or 1 μm). Data are depicted as the mean ± SEM. Statistical significance was determined by one-way and two-way ANOVA with a post hoc Holm–Sidak test, ns not significant; *P < 0.05; **P < 0.01; ***P < 0.001; ****P < 0.0001.

The contribution of autophagy to DOX-induced cardiac injury is well-documented [15]. To evaluate the effect of DIC on autophagy in GSDMD inhibition hearts, we first performed time-point analysis of hearts 1, 3, 5, and 7 d after DOX injection. Western blotting revealed the same trends in the levels of GSDMD and microtubule-associated protein-1 light chain 3-II (LC3II), a marker of autophagy. Specifically, protein levels began to increase 1 d after DOX intervention, peaked after 3 d, and began to decline after 5 d (Fig. 4A, B). We then determined the expression of several autophagy-associated genes, including ATGs, Beclin-1, LC3B, and Sqstm 1/*p*62 genes. The expression of most genes showed a similar trend to that of protein expression (Fig. 4C), reflecting the response to DOX. Therefore, we selected day 3 as the time of cardiac intervention in mice for subsequent exploration of autophagy.

We quantified the levels of LC3-II protein in mouse heart. While the levels were increased by DOX administration, the increase was reduced in the GSDMD-CKO background, and partially rescued by AAV9-GSDMD-OE infection (Fig. 4D, E). LC3-II level increase could be associated with the initiation of autophagy or prevention of LC3-II degradation. To distinguish between these two possibilities, we used bafilomycin A1 (BAFA1), a lysosomal inhibitor of late-stage autophagy that blocks the degradation of LC3-II [28]. We observed that LC3-II levels were obviously increased in DOX-treated heart and further increased upon BAFA1 treatment (Fig. 4F, H). However, the increase was much lower in DOX-treated GSDMD-CKO heart than in the GSDMD^(flox/flox)^ DOX-treated heart (Fig. 4F). Further, the reduced LC3-II levels in GSDMD-deficient heart were rescued by GSDMD overexpression (Fig. 4G, I). These observations suggest that LC3-II accumulation in GSDMD-overexpressing heart and DOX-treated heart is caused by its blocked degradation.

We next used transmission electron microscopy (TEM) to investigate the autophagosomes and autolysosomes in the heart of DOX-treated mice. GSDMD-CKO mice were relatively resistant to DOX injection, with a reduced number of autophagosomes and autolysosomes (Fig. 4J). However, a marked increase in autophagosomes and autolysosomes was observed in AAV9-GSDMD-OE DOX-treated mice compared with that in control mice (Fig. 4K). This indicated that GSDMD promotes DOX-induced myocardial autophagy.

### DOX activates cardiomyocyte autophagic flux and GSDMD aggravates myocardial autophagy levels in adult cardiomyocytes

**Fig. 5:**
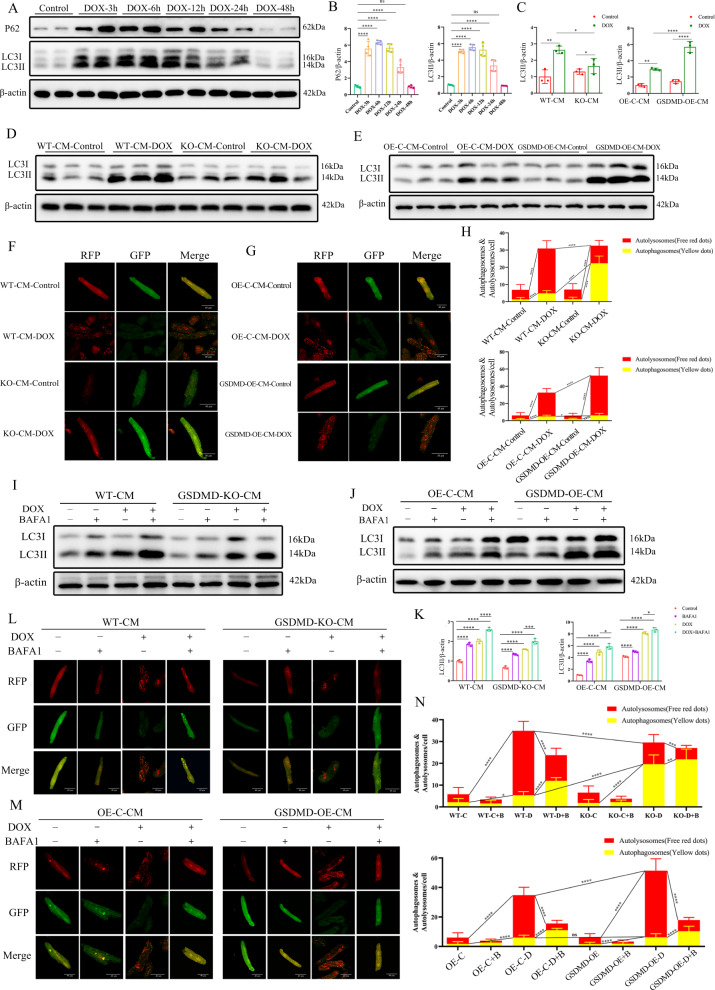
DOX activates cardiomyocyte autophagy, and GSDMD aggrevates myocardial autophagy levels in adult cardiomyocytes. **A**, **B** Autophagy-related genes (including p62/Sqstm1 and LC3) at different time points after DOX treatment in cardiocyocytes. Quantification of LC3II/β-actin and p62/β-actin was analyzed. *N* = 4 per group. **C**–**E** Immunoblotting of LC3 in WT and GSDMD-KO or GSDMD-overexpression cardiomyocytes treated with saline or DOX and quantifications are shown. *N* = 3 per group. **F**–**H** Representative images of RFP (red fluorescent protein)-GFP (green fluorescent protein)-LC3 (microtubuleassociated protein 1A/1B-light chain 3) puncta in WT and KO/OE cardiomyocytes treated with saline or DOX and the number of autophagosomes (yellow dots) and autolysosomes (red dots) per cell. The *P* values on the yellow or red lines represent the comparison between the corresponding autophagosome or autolysosome groups, respectively. Scale bar, 40 μm. **I**–**K** Autophagic flux was assessed by western blot using BAFA1 in cardiomyocytes of different groups. Quantification of LC3II/β-actin was analyzed. *N* = 3 per group. **L**–**N** Representative images of mRFP-LC3 puncta (red) and GFP-LC3 puncta (green) after treated with DOX and/or BAFA1 and quantified red puncta number and yellow puncta number in different groups of cardiomyocytes. Scale bar, 40 μm. Data are depicted as the mean ± SEM. Statistical significance was determined by one-way and two-way ANOVA with a post hoc Holm–Sidak test, ns, not significant; *P < 0.05; **P < 0.01; ***P < 0.001; ****P < 0.0001.

In vivo studies verified the relationship between GSDMD and autophagy. We next explored whether this effect could be reproduced in cultured cardiomyocytes. The levels of LC3-II protein in DOX-treated cardiomyocytes first increased and then fell, reaching a peak 6–12 h after DOX intervention (Fig. 5A, B). We, therefore, chosed 12 h as the time point for cardiomyocyte autophagy studies. DOX markedly induced the accumulation of autophagy-associated proteins in the WT group, but the levels were more reduced in cardiomyocytes isolated from adult GSDMD-KO mice (Fig. 5C, D). By contrast, LC3-II accumulated in GSDMD-overexpressing cardiomyocytes (Fig. 5C, E). We concluded that DOX promotes autophagy induction, contributing to a high level of LC3-II expression, with GSDMD enhancing this effect.

To identify the specific stages of autophagic flux induced by DOX, we transfected cardiomyocytes with a tandem double-labeled probe, monomeric red fluorescent protein–green fluorescent protein–LC3 (mRFP-GFP-LC3). The cells were then incubated with DOX for 12 h. Since GFP protein is sensitive to low pH, only red fluorescence is detected upon GFP fluorescence after autophagosome and lysosome fusion, as mRFP fluorescence is stable [28]. This allows the assessment of autophagic flux, distinguishing autophagosomes (yellow) from autolysosomes (red). Accordingly, red puncta are more numerous than yellow puncta upon autophagy induction, with opposite observations being made upon autophagy inhibition.

We observed a remarkable increase in the number of autolysosomes and autophagosomes in DOX-treated WT cardiomyocytes, with the former more significant than the latter (Fig. 5F). GSDMD deficiency reduced the autolysosome to autophagosome ratio compared with that in the DOX-treated WT cardiomyocyte group (Fig. 5F, H), whereas GSDMD overexpression had the opposite effect (Fig. 5G, H). This suggested that downregulation of GSDMD reduces autophagy. These observations, together with immunoblot analysis (Fig. 5A–E), indicated that DOX treatment promotes autophagy, and that GSDMD overexpression enhances this effect.

To verify the above conclusions, we used BAFA1 to prevent the fusion of autophagosomes with lysosomes. The enhanced expression of LC3-II protein upon DOX treatment further increased in the presence of BAFA1 (Fig. 5I–K). By contrast, as anticipated, LC3-II levels were decreased in GSDMD-KO cardiomyocytes (Fig. 5I, K) and were significantly increased in GSDMD-overexpressing cells (Fig. 5J, K). We then repeated the autophagic flux analysis using the mRFP-GFP-LC3 reporter system introduced by AAV infection. BAFA1 drastically reduced the number of autolysosomes in WT, GSDMD-KO-CM, and GSDMD-OE-CM groups compared with that in the controls, with and without DOX treatment, but increased the number of autophagosomes (Fig. 5L–N). In other words, the autolysosome to autophagosome ratio significantly decreased upon BAFA1 treatment, suggesting that the autophagic flux was inhibited, especially in GSDMD-KO cells. Together with western blot data (Fig. 5I–K), these observations confirmed that BAFA1 induces the aggregation of LC3-II by inhibiting LC3-II degradation at the late stage of autophagy, and that downregulation of GSDMD expression weakens this effect.

### GSDMD deletion ameliorates DIC by inhibiting autophagy

The role of autophagy reduction by GSDMD inhibition was further investigated using the autophagy inhibitor 3-methyladenine (3-MA) and autophagy agonist rapamycin (Rapa) in vivo. 3-MA inhibits the formation of autophagosomes, and Rapa is an inhibitor of mechanistic target of rapamycin (mTOR) [29]. As anticipated, intraperitoneal injection of 3-MA and Rapa decreased BW in DOX-treated groups, but the weight loss was more pronounced in Rapa-treated groups and declined to a lesser extent in DOX-treated CKO mice than in other mice, while the heart weight to tibia length (HW/TL) ratio showed the opposite trend (Fig. S10A). Cardiac function improved after 3-MA treatment, with an increased ejection fraction and fractional shortening, while Rapa treatment exacerbated cardiac function (Fig. S10B, S10E, and S10F). Echocardiography readings for the DOX-treated CKO group were significantly better than those for the control group (Fig. S10C). Furthermore, 3-MA markedly decreased the LC3-II levels, regardless of DOX treatment (Fig. S10D). Meanwhile, Rapa induced accumulation of LC3-II and apoptosis-related proteins, and fully abolished 3-MA–induced decrease in these protein levels upon DOX administration; these protein levels were slightly decreased in CKO mice (Fig. S10D, S10G–S10I). Hence, the downregulation of GSDMD levels in the heart during DIC ameliorates the impairment of autophagy.

### RNA-sequencing (RNA-seq) reveals potential target genes of GSDMD

**Fig. 6:**
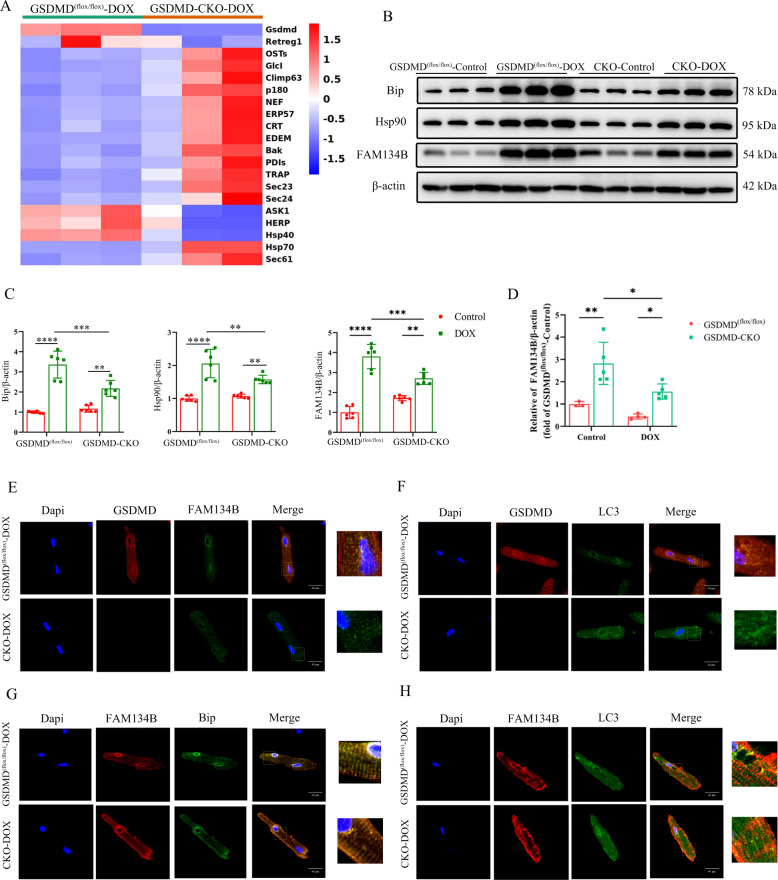
RNA-seq revealed potential target genes of GSDMD. **A** Heatmaps show differentially expressed genes between GSDMD^(flox/flox)^-DOX and GSDMD-CKO-DOX mice based on RNA-seq data. **B**, **C** Western blot analysis of endoplasmic reticulum related genes, Bip, Hsp90, and FAM134B. Representative immunoblots and quantifications are shown. *N* = 6 per group. **D** q-PCR analysis of FAM134B in GSDMD^(flox/flox)^ and GSDMD-CKO hearts. *N* = 3–5 per group. **E**–**H** Representative GSDMD and FAM134B expression, GSDMD and LC3 expression, FAM134B and Bip expression, FAM134B and LC3 expression, and colocalization in cardiomyocytes as shown by immunofluorescence analysis in adult mice hearts after DOX treatment. Scale bar, 40 μm. Data are depicted as the mean ± SEM. Statistical significance was determined by one-way and two-way ANOVA with a post hoc Holm–Sidak test, ns not significant; *P < 0.05; **P < 0.01; ***P < 0.001; ****P < 0.0001.

The above experiments suggested that GSDMD deficiency can play a protective role in the myocardium by inhibiting cardiac autophagy. However, how GSDMD mediates cardiac autophagy remains unclear. To further investigate it, we explored GSDMD-mediated regulatory genes using RNA-seq. The analysis revealed a significant change in the expression of ER-related genes between DOX-treated GSDMD CKO mice and control mice (Fig. 6A). ERS can lead to the accumulation of unfolded or misfolded proteins in the ER lumen and induce autophagy in different ways. Western blot analysis indicated that ERS-related proteins were significantly downregulated after GSDMD inhibition (Fig. 6B, C), further suggesting that GSDMD may induce cardiac autophagy by regulating ERS. Among these ER injury-related genes, FAM134B might be a special one. FAM134B is a common receptor involved in ER autophagy and mediates the recognition and removal of ER by autophagosomes [30, 31]. Downregulation of FAM134B leads to ER-phagy, causing ER expansion, ERS, and cell death [30]. Real-time PCR and western blotting indicated upregulation of ER-regulatory protein Retreg1 (FAM134B) after DOX intervention and its downregulation during GSDMD deficiency (Fig. 6B–D). We observed that the ER marker FAM134B colocalized with GSDMD and presented as positive spots upon immunofluorescence staining (Fig. 6E). This revealed that GSDMD and FAM134B were expressed in the cardiomyocyte ER, with FAM134B downregulation upon GSDMD deletion (Fig. 6E). GSDMD and LC3 (Fig. 6F), FAM134B and Bip (Fig. 6G), and FAM134B and LC3 (Fig. 6H) also colocalized in cardiomyocytes. Co-Immunoprecipitation also revealed that GSDMD and FAM134B, GSDMD, and LC3 colocalized (Fig. S11A), which were consistent with immunofluorescence staining results. Taken together, these results suggested that GSDMD may cause cardiac autophagy and cardiomyocyte apoptosis by regulating ER-phagy receptor FAM134B.

### GSDMD promotes autophagy by promoting ERS to activate FAM134B, thus aggravating cardiomyocyte apoptosis

To demonstrate whether GSDMD attaches to ER and forms pores, we extracted ER proteins and verified the efficiency (Fig. S11B). Calreticulin is a molecular chaperone of ER when upregulated in response to ERS. Subsequently, the extracted cardiac ER proteins were used for western blot quantitative analysis as ER-related proteins, and it was found that the levels increased after DOX administration and decreased after GSDMD-CKO (Fig. S11C and 11D). Immunofluorescence staining also revealed that GSDMD colocalized with Bip (Fig. S12A). Importantly, TEM showed that more pores appeared in the ER membrane in the DOX treatment group than in the control group, further confirming the attachment of GSDMD to the ER for pore formation (Fig. S12B).

**Fig. 7:**
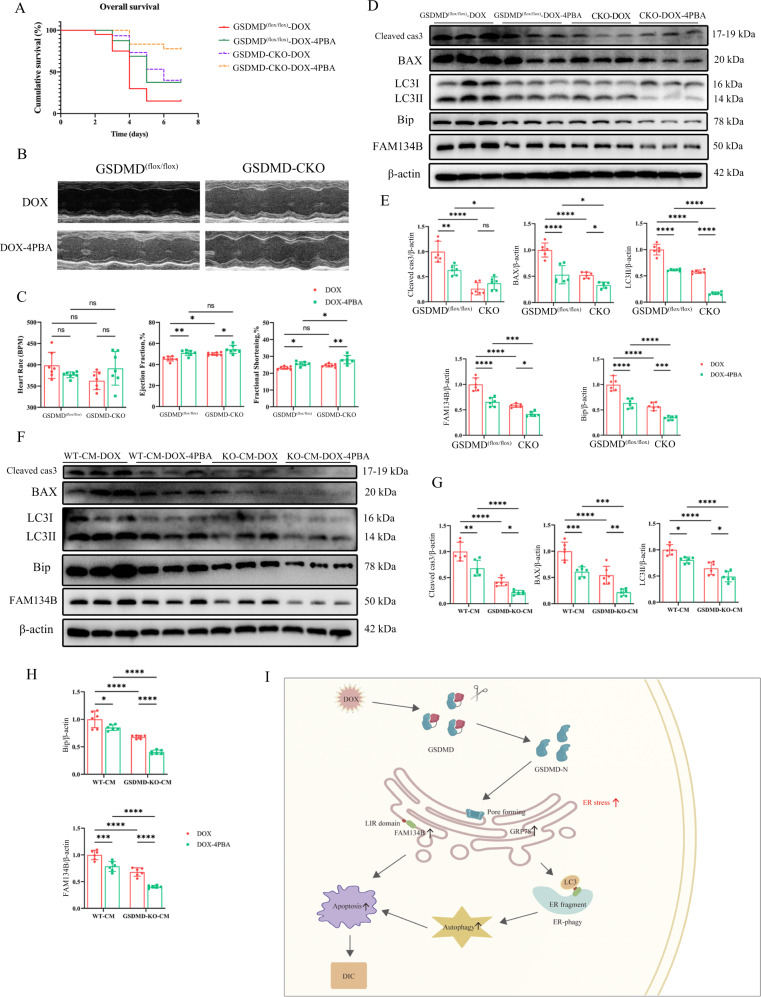
FAM134B functions downstream of GSDMD/ERS to regulate DIC. **A** Survival curve of GSDMD^(flox/flox)^ and GSDMD-CKO mice after DOX and 4PBA treatments was created by Kaplan–Meier method. *N* ≥ 15 per group. **B**, **C** Representative echocardiograms from GSDMD^(flox/flox)^ and GSDMD-CKO mice treated with DOX and 4PBA are shown. Heart rate (HR), ejection fraction (EF) and fractional shortening (FS) are measured by echocardiography. *N* = 7 per group. **D**, **E** Western blot detection of Cleaved-caspase 3 (Cleaved cas3), BAX, LC3, Bip, and FAM134B protein abundance from mice after DOX and 4PBA treatments. Quantification of protein abundance as illustrated in panel. *N* = 6 per group. **F**–**H** Western blot detection of Cleaved-caspase 3 (Cleaved cas3), BAX, LC3, Bip, and FAM134B protein abundance in cardiomyocytes after DOX treatments. Quantification of protein abundance as illustrated in panel. *N* = 6 per group. **I** Working model by which how GSDMD induces cardiotoxicity after DOX administration. Data are depicted as the mean ± SEM. Statistical significance was determined by one-way and two-way ANOVA with a post hoc Holm–Sidak test, ns, not significant; *P < 0.05; **P < 0.01; ***P < 0.001; ****P < 0.0001.

To determine whether GSDMD plays a role in regulating ERS, we used the ERS inhibitor 4-phenylbutyric acid (4PBA) to inhibit ERS and observed changes in cardiac autophagy and cardiomyocyte apoptosis. 4PBA inhibited ERS and autophagy in GSDMD^(flox/flox)^ mice and neutralized the effects of GSDMD deficiency on ERS and autophagy (Fig. 7D, E). Further, 4PBA reduced GSDMD^(flox/flox)^ mouse mortality (Fig. 7A), improved cardiac function (Fig. 7B, C), and decreased cardiomyocyte apoptosis (Fig. 7D, E). In vitro data were consistent with the in vivo observation that 4PBA reduces ERS and apoptosis (Fig. 7F–H). These findings confirmed that GSDMD loss inhibits cardiac autophagy, reduces myocardial apoptosis, improves cardiac function, and alleviates DIC by mediating ERS in cardiomyocytes.

Furthermore, the expression of FAM134B in GSDMD^(flox/flox)^ mice was remarkably downregulated after 4PBA administration and was further downregulated in the CKO group (Fig. 7D), demonstrating that FAM134B is a key downstream target gene of GSDMD regulating ERS. In summary, the presented results indicate that GSDMD-N activates ERS by promoting ER perforation, stimulating FAM134B to fragment ER, thus promoting autophagy, aggravating cardiomyocyte apoptosis, and ultimately aggravating DIC (Fig. 7I).

## Discussion

To the best of our knowledge, this study provides the first direct evidence that GSDMD is upregulated in DIC, exacerbates cardiac injury, and impairs cardiac function by promoting cardiomyocyte apoptosis and autophagy. Here, we found that GSDMD-N is significantly upregulated in DIC. In addition, cardiomyocyte pyroptosis mainly occurred in the early stage of DIC; however, many cardiomyocytes underwent apoptosis in the late stage. GSDMD-N attaches to the ER and forms pores, resulting in increased ROS production, which activates the expression of FAM134B and induces ERS. Subsequently, FAM134B binds to LC3 to induce autophagy (Fig. 7I). These findings thus reveal the previously undiscovered roles of GSDMD and FAM134B in DIC and provide novel insights into autophagic regulation mechanisms.

DOX exerts pleiotropic effects in cardiotoxicity via multiple mechanisms, including myocardial apoptosis, autophagy, oxidative stress, mitochondria damage, and Ca^+^ disturbance [32, 33]. Apoptosis is characterized by chromatin condensation, DNA fragmentation, cell shrinkage, and the formation of apoptotic bodies [34, 35]. We observed a significant elevation of myocardial apoptosis and deterioration of cardiac function in the constructed DIC models, suggesting that DOX causes apoptosis. Pyroptosis is another form of programmed cell death, with GSDMD playing a key role. GSDMD inhibition reportedly significantly reduces cardiomyocyte pyroptosis and I/R-induced myocardial injury, and cardiomyocyte pyroptosis is mainly regulated by the caspase-11/GSDMD signaling pathway under oxidative stress [12]. The NF-κB/GSDMD axis is also important in bridging the oxidative stress response and cardiomyocyte pyroptosis after MI [36]. Similarly, herein, we found that pyroptosis plays an important role in I/R and MI. However, the role of GSDMD in DIC remains elusive. Interestingly, we found that cardiomyocyte pyroptosis mainly occurred in the early stage of DIC, while massive cardiomyocyte apoptosis occurs in the late stage. Therefore, we subsequently studied the effect of cardiomyocyte apoptosis in DIC. We found that DOX treatment induces an increase in GSDMD levels, suggesting that GSDMD may be involved in the occurrence of tumor-related heart disease and is mainly regulated by the caspase-11/GSDMD signaling pathway. Accordingly, GSDMD-KO and AAV9-GSDMD-OE mice were obtained to explore the specific underlying mechanism. We observed that GSDMD deletion alleviated DIC and that mortality and cardiac injury increased after GSDMD overexpression in mice. Although cardiomyocytes account for a large proportion of the heart tissue, which is composed of a variety of cells, we could not exclude experimental interference from other cells. Hence, to investigate whether GSDMD plays a role in DIC, we constructed DIC models in GSDMD-CKO mice. In vitro and in vivo analyses showed that cardiomyocyte-specific GSDMD KO exacerbated apoptosis and DIC.

Autophagy is a process whereby the cell degrades damaged organelles and macromolecules using lysosomes and is regulated by autophagy-related genes [37]. Previous studies have reported on the relationship between DOX and autophagy, but the issue remains controversial. DOX reportedly blocks cardiomyocyte autophagic flux mainly by driving lysosomal acidification and lysosomal function [15]. However, another study indicated that early autophagy flux in adult zebrafish heart after DOX intervention is not blocked but upregulated [38]. How autophagy regulates and protects cardiomyocyte function in DIC is still controversial, and the outcomes mainly depend on the DOX dose and observation time. Considering the lack of clarity on the relationship between autophagy and DOX, we here used autophagic regulators (BAFA1, 3-MA, and Rapa) to clarify the mechanism regulating autophagy in DIC. Accordingly, 3-MA significantly decreased the expression of LC3-II in DOX-treated mice, while BAFA1 and Rapa significantly increased the levels of LC3-II protein, which confirmed that BAFA1 and 3-MA inhibited autophagy by blocking the autophagosome–lysosome fusion and autophagosome formation, respectively. Further, experiments using the mRFP-GFP-LC3 probe revealed an increase in autophagic flux upon DOX treatment. These observations suggest enhanced cardiac autophagic flow and aggravated cardiac autophagy upon DOX intervention. We also found that autophagy is downregulated by GSDMD deficiency but enhanced by GSDMD overexpression, suggesting that GSDMD deficiency may play a protective role in the myocardium by blocking myocardial autophagy.

The ER is an organelle in which proteins fold to function. High expression of GRP78 at baseline is considered to be a marker of cellular ERS [39–41]. Furthermore, enhanced expression of GRP78 in ischemic cardiomyopathy and heart failure was reported [42–44]. We showed here that the expression of GRP78 was significantly upregulated in DIC and the expression of Hsp90, another ERS marker, was also increased. The expression of both proteins was decreased after GSDMD deletion, indicating that DOX caused ERS and that GSDMD deletion alleviated this effect.

The N-terminal domain of GSDMD specifically binds to phosphoryl phosphatidylinositol or cardiolipin, which are unique to the eukaryotic and prokaryotic cell membrane, respectively, and forms pores [2]. Since the ER membrane contains phosphatidylinositol, we speculated that GSDMD-N could bind to ER membrane and perforate it, leading to ER oxidative stress. Indeed, TEM analysis confirmed the presence of multiple pores in the ER membrane after DOX intervention. ERS suppression resulted in decreased cardiomyocyte apoptosis and improved mouse survival after GSDMD deletion compared with control mice. Our study demonstrated that GSDMD could mediate ER membrane perforation and activate ERS to promote cardiomyocyte apoptosis, while inhibition of ERS could alleviate cardiomyocyte apoptosis.

Finally, ERS can trigger autophagy. ER-phagy, a selective autophagic degradation of the ER, is a regulatory mechanism maintaining ER homeostasis under stress conditions and can reduce ERS [45]. ER-phagy is regulated by several receptors, namely, reticular phagocytic regulator 1 (also known as FAM134B or JK1), reticulon 3, preprotein translocus factor SEC62, cell cycle progression 1, testis-expressed gene 264, and atlastin GTPase3 [46–48]. FAM134B is the first identified selective ER phagocytic receptor protein anchored at the ER and is considered to be the most characteristic ER phagocytic receptor in mammalian cells. It mediates the recognition and removal of the ER by autophagosomes [30]. Downregulation of FAM134B eliminates ER-phagy, leading to ER expansion, ERS, and human nerve cell death [30]. Further, FAM134B has two specific domains: reticular homologous domain (RHD; N-terminal) and LIR motif (C-terminal) [30]. LIR binds to LC3/GABARAP family proteins and accumulates autophagy. Herein, we showed that ER oxidative stress activates FAM134B, which causes ER fragmentation and binding of its LIR motif to LC3 to induce autophagy, while inhibiting FAM134B reduces autophagy, thus reducing DIC. We also showed that ERS is caused by GSDMD forming pores in the ER.

Collectively, herein, we revealed a new interaction between GSDMD and FAM134B in DIC. On the one hand, GSDMD-N causes ER oxidative stress by forming pores in the ER membrane. Oligomerization of activated FAM134B causes ER membrane fragmentation, which is subsequently recognized and enveloped by autophagosomes after a direct interaction between FAM134B and LC3, further leading to cardiomyocyte autophagy. On the other hand, DOX directly causes ERS and induces cardiomyocyte apoptosis. Together, these data suggest that GSDMD may be a potential biomarker and therapeutic target for DIC, and inhibition of GSDMD provides new insights into the treatment of tumor-related heart disease.

## Supplementary information

WB(full length)

Supplementary Figure 1

Supplementary Figure 2

Supplementary Figure 3

Supplementary Figure 4

Supplementary Figure 5

Supplementary Figure 6

Supplementary Figure 7

Supplementary Figure 8

Supplementary Figure 9

Supplementary Figure 10

Supplementary Figure 11

Supplementary Figure 12

supplementary figure legends

supplementary materials

A reproducibility checklist

supplementary materials table 1

## Data Availability

Upon reasonable request, the corresponding author will provide data to support the results of this study. Please refer to the main resources in the Supplementary Information.
