# Correction to: Gasdermin D mediates endoplasmic reticulum stress via FAM134B to regulate cardiomyocyte autophagy and apoptosis in doxorubicin-induced cardiotoxicity

**DOI:** 10.1038/s41419-022-05400-9

**Published:** 2022-11-22

**Authors:** Ya’nan Qu, Rifeng Gao, Xiang Wei, Xiaolei Sun, Kun Yang, Huairui Shi, Yang Gao, Shiyu Hu, Yiwen Wang, Ji’e Yang, Aijun Sun, Feng Zhang, Junbo Ge

**Affiliations:** 1 Department of Cardiology, Shanghai Institute of Cardiovascular Diseases, Zhongshan Hospital, Fudan University, 200032 Shanghai, China; 2 Department of Cardiology, Shanghai Fifth People’s Hospital, Fudan University, 200240 Shanghai, China; 3 Key Laboratory of Viral Heart Diseases, National Health Commission, 200032 Shanghai, China; 4 Key Laboratory of Viral Heart Diseases, Chinese Academy of Medical Sciences, 200032 Shanghai, China; 5 National Clinical Research Center for Interventional Medicine, 200032 Shanghai, China; 6 Institutes of Biomedical Sciences, Fudan University, 200032 Shanghai, China

**Keywords:** Apoptosis, Autophagy, Cardiovascular diseases

Correction to: *Cell Death and Disease* 10.1038/s41419-022-05333-3, published online 26 October 2022

The original version of this article contained an error in the corresponding authorship. The authors state the following: Feng Zhang was also a corresponding author and made an important contribution to our article. In the version submitted in the submission system and e.Proofing system, Feng Zhang appears in the manuscript as one of the corresponding authors (his email address is listed in the manuscript). Unfortunately, we found that Feng Zhang was not listed as a corresponding author in the published article online. We all informed and consented to his appointment as a corresponding author. The original article has been corrected.

