# Supplementary material for: Gasdermin D mediates endoplasmic reticulum stress via FAM134B to regulate cardiomyocyte autophagy and apoptosis in doxorubicin-induced cardiotoxicity
Source: Cell Death Dis. 2022 Oct 26;13(10):901. doi: 10.1038/s41419-022-05333-3 (PMC9606128; doi:10.1038/s41419-022-05333-3)

Figure 1

E

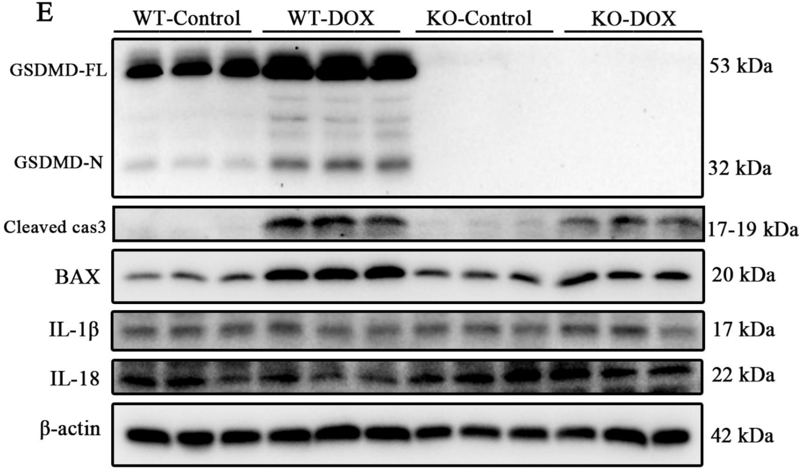
WT-Control
WT-DOX
KO-Control
KO-DOX
GSDMD-FL
GSDMD-N
Cleaved cas3
BAX
IL-1β
IL-18
β-actin
53 kDa
32 kDa
17-19 kDa
20 kDa
17 kDa
22 kDa
42 kDa

GSDMD

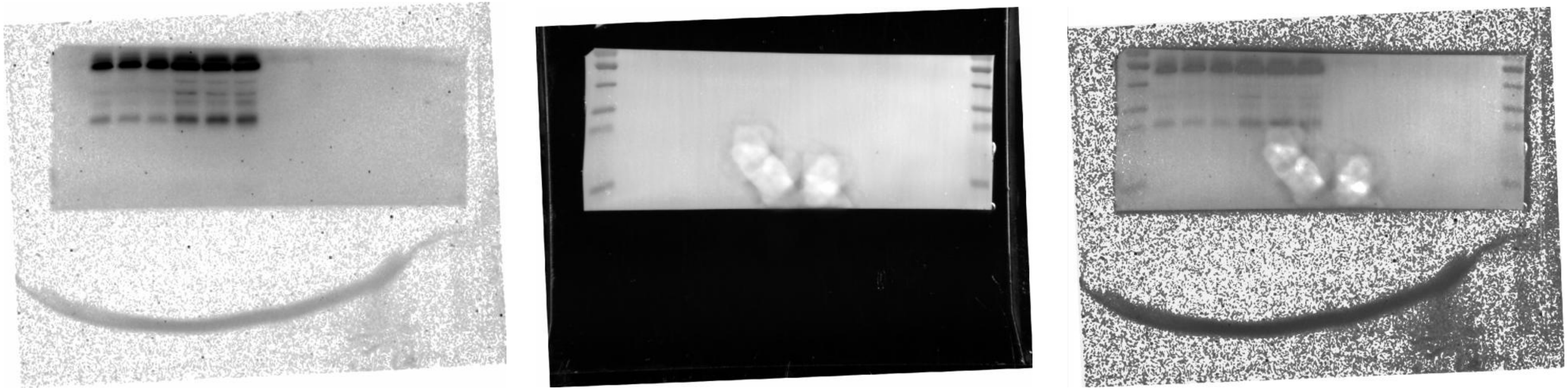

BAX

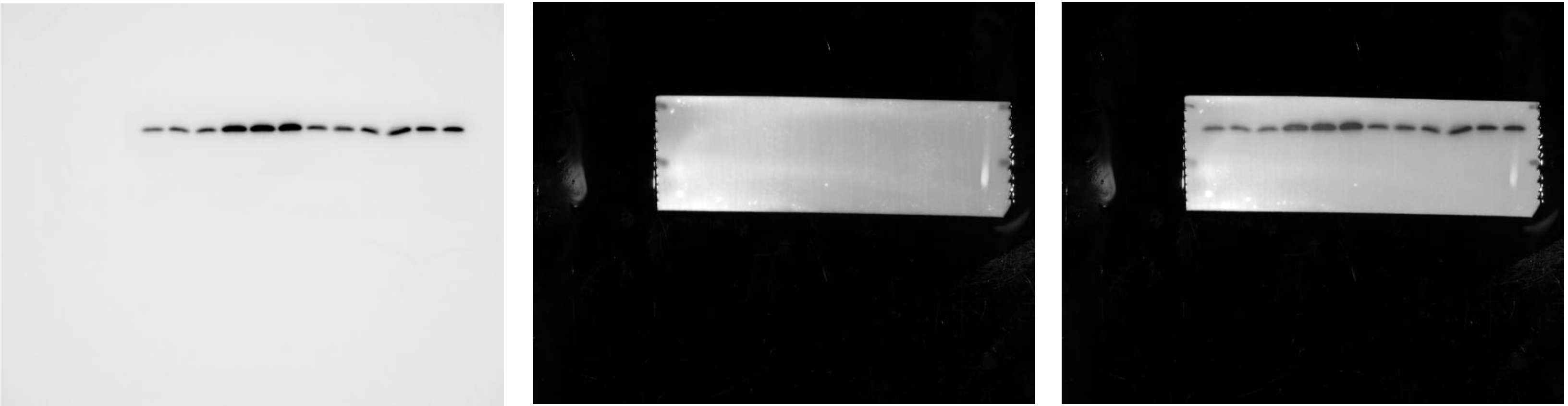

Cleaved cas3

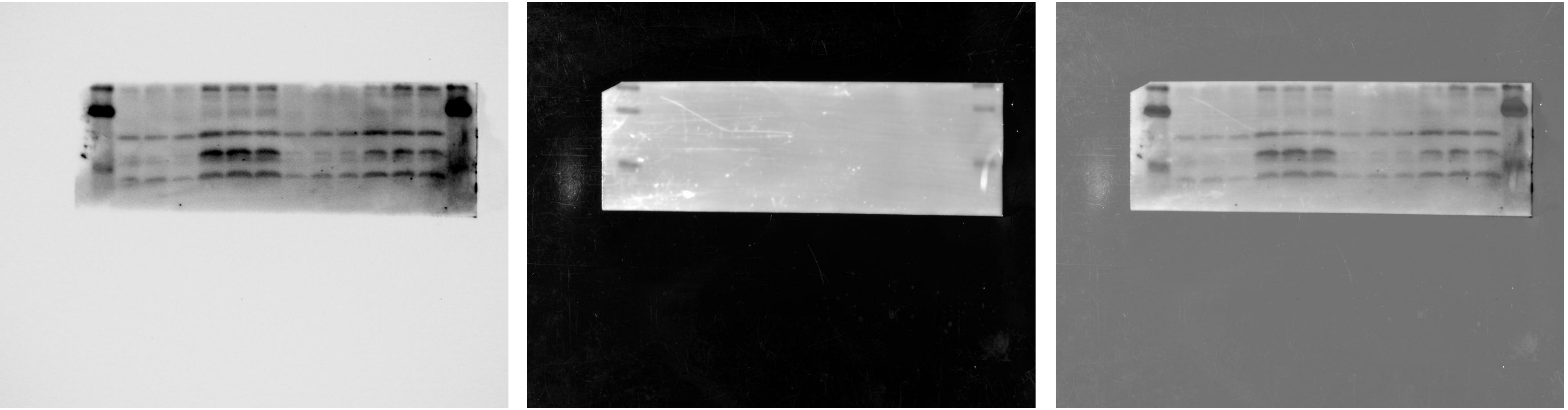

IL-1β

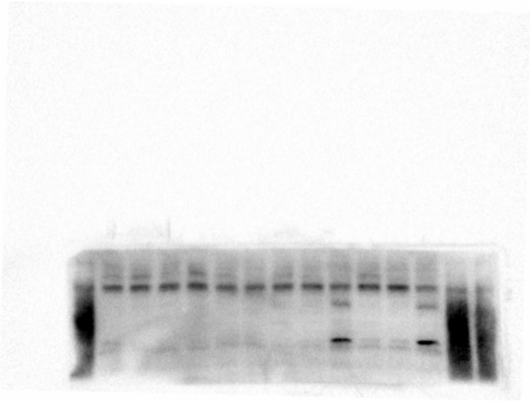

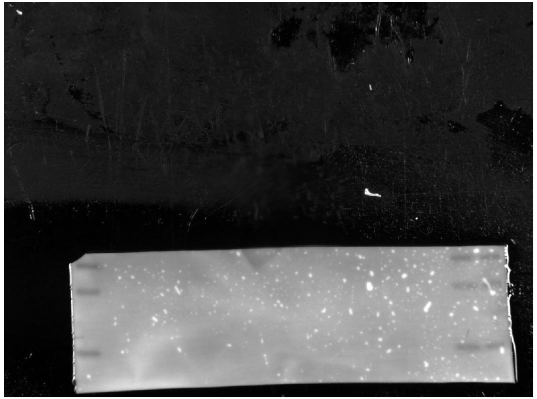

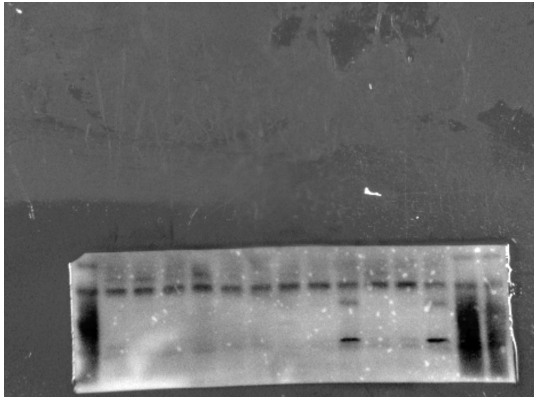

IL-18

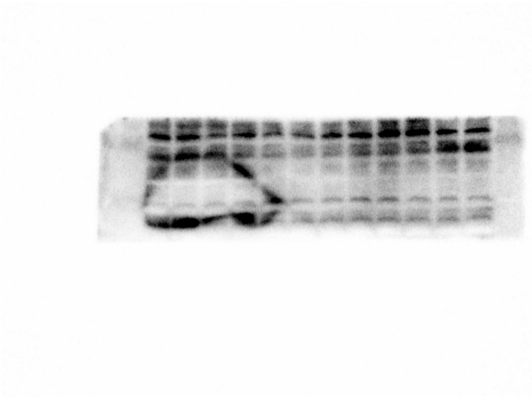

β-actin

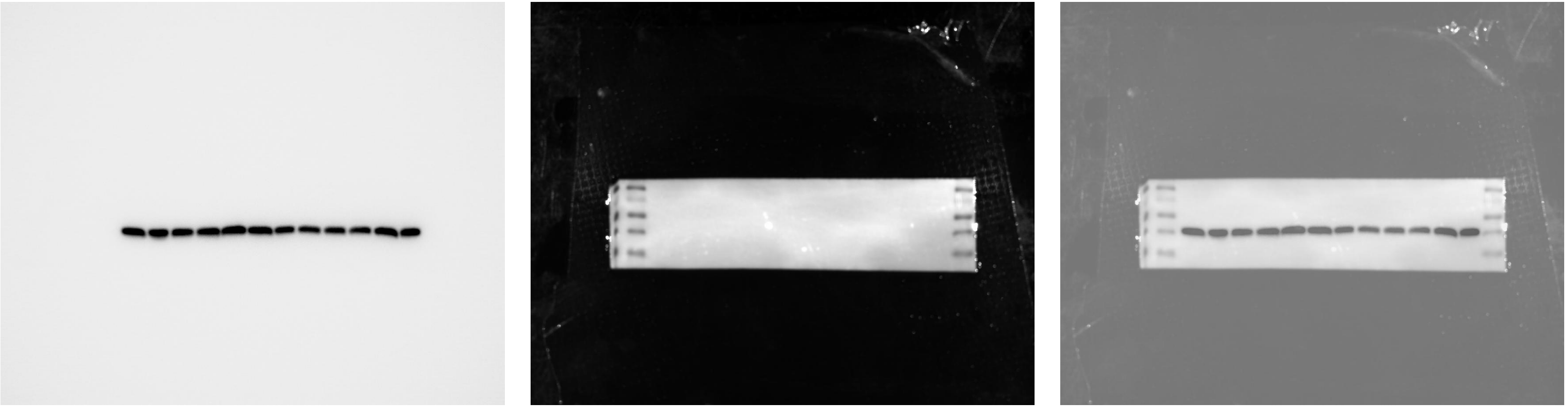

L

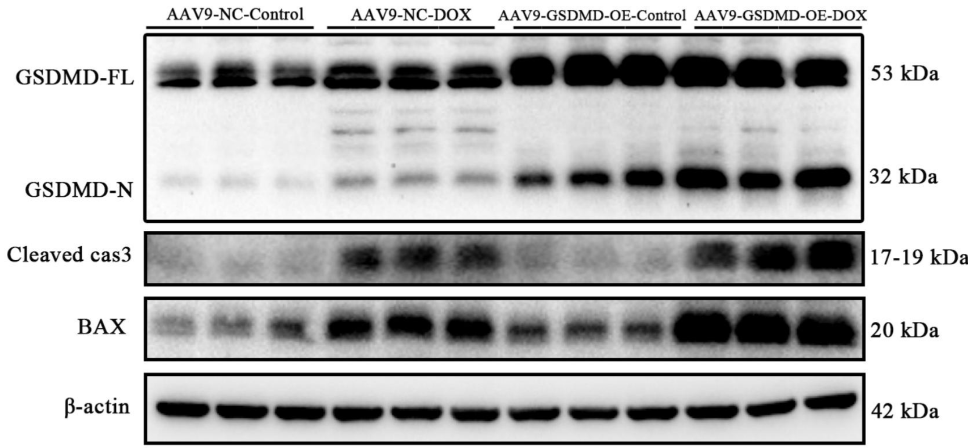
AAV9-NC-Control
AAV9-NC-DOX
AAV9-GSDMD-OE-Control
AAV9-GSDMD-OE-DOX
GSDMD-FL
53 kDa
GSDMD-N
32 kDa
Cleaved cas3
17-19 kDa
BAX
20 kDa
β-actin
42 kDa

GSDMD

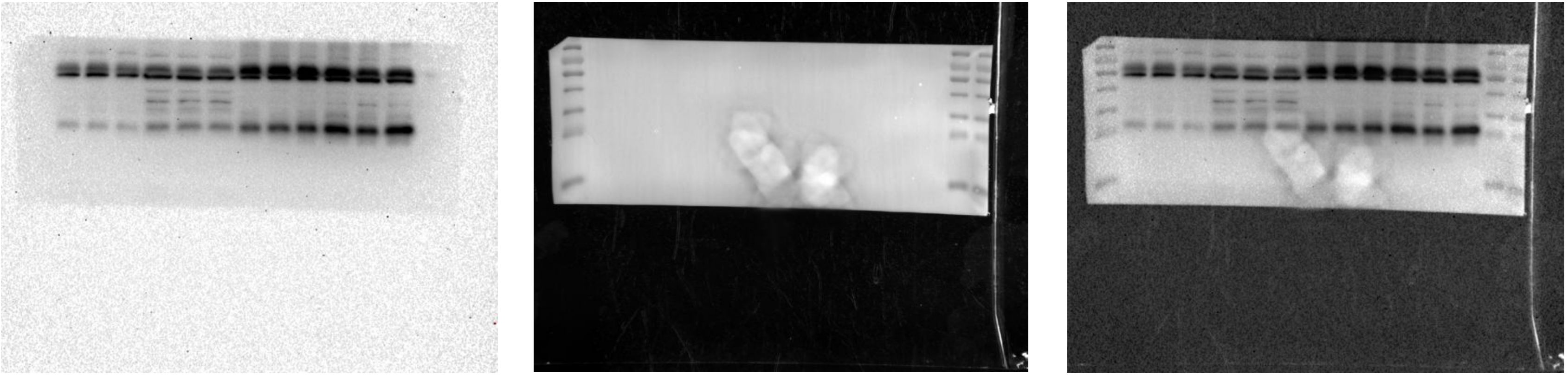

BAX

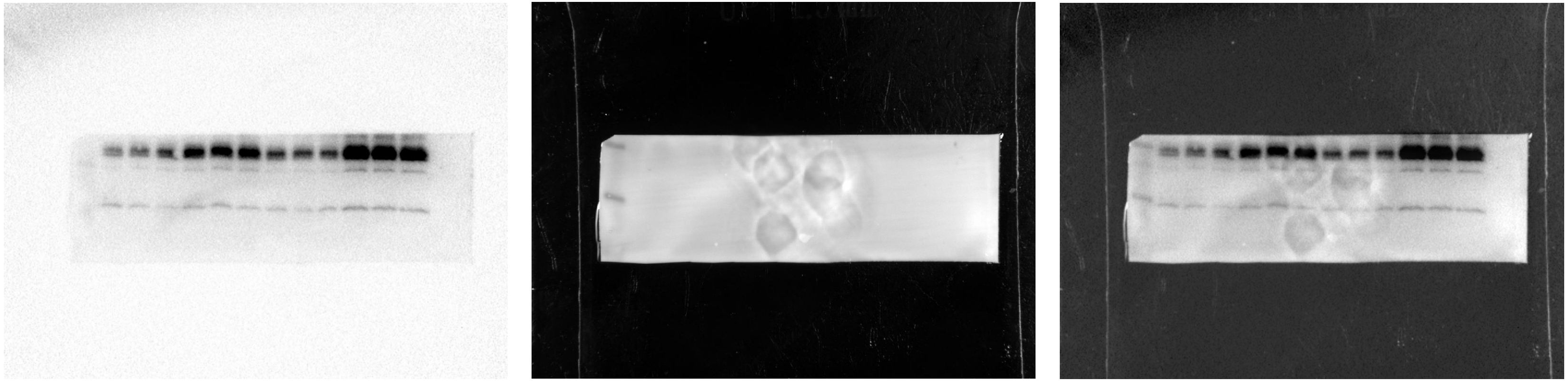

Cleaved cas3

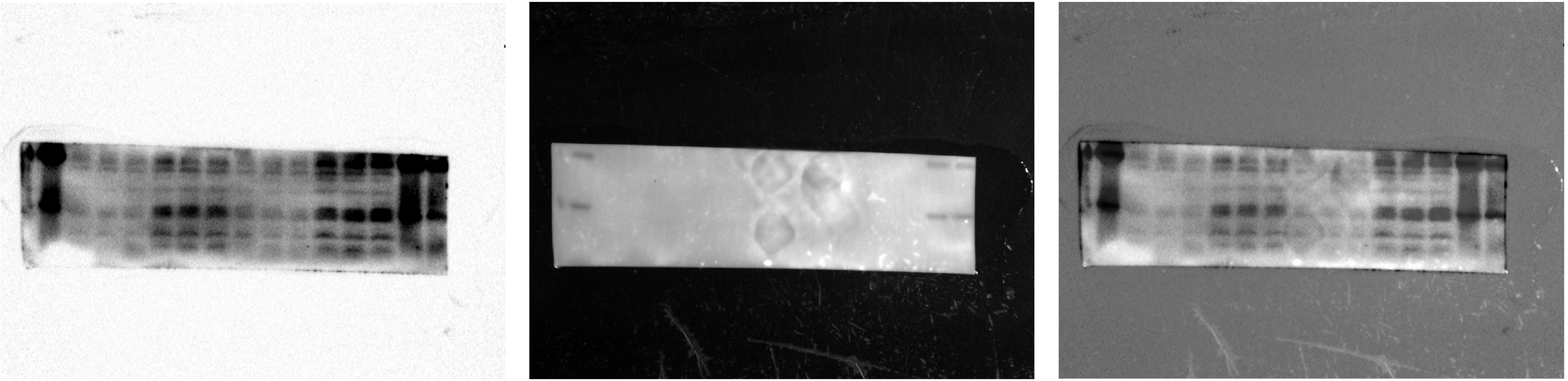

β-actin

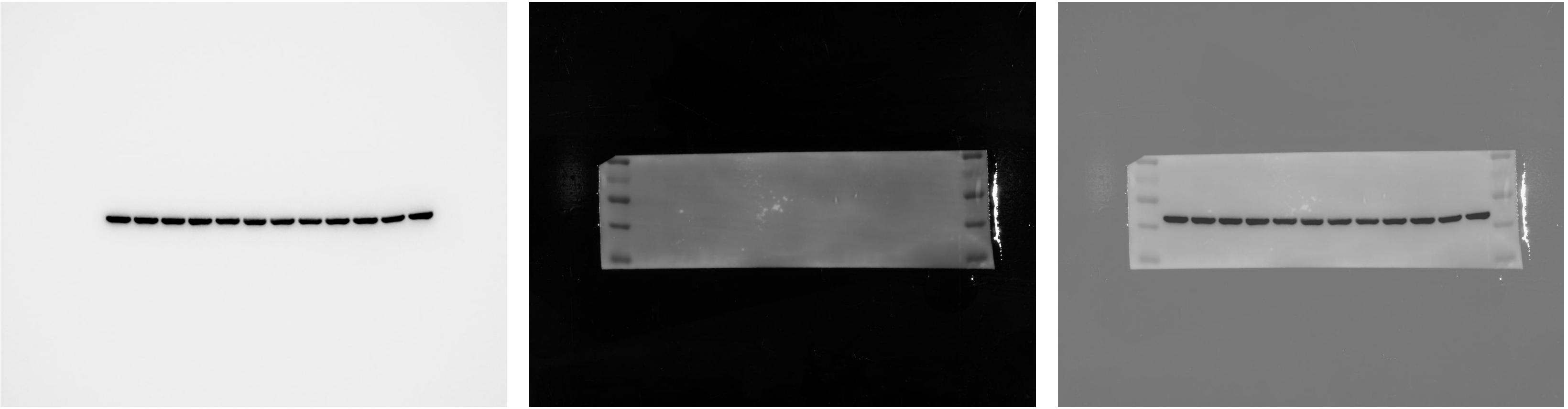

Figure 2

M

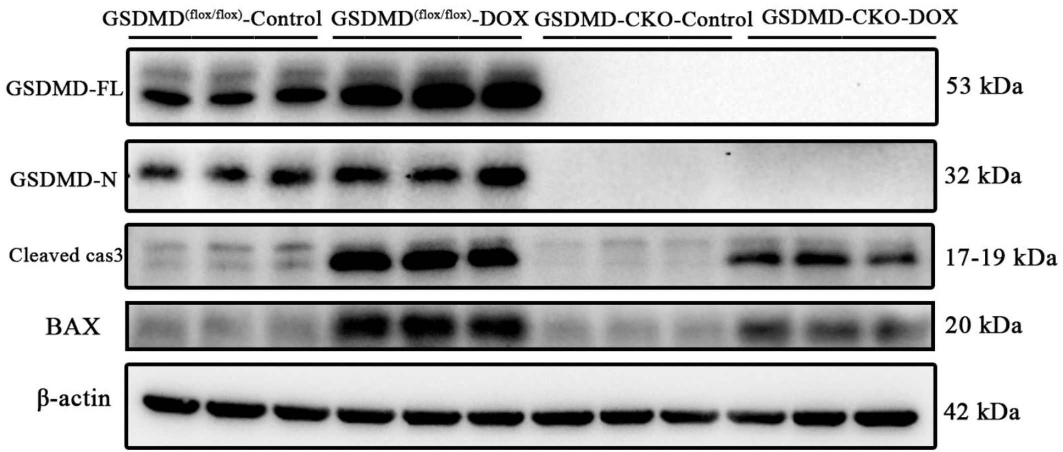
GSDMD(flox/flox)-Control
GSDMD(flox/flox)-DOX
GSDMD-CKO-Control
GSDMD-CKO-DOX
GSDMD-FL
GSDMD-N
Cleaved cas3
BAX
β-actin
53 kDa
32 kDa
17-19 kDa
20 kDa
42 kDa

N

GSDMD-FL

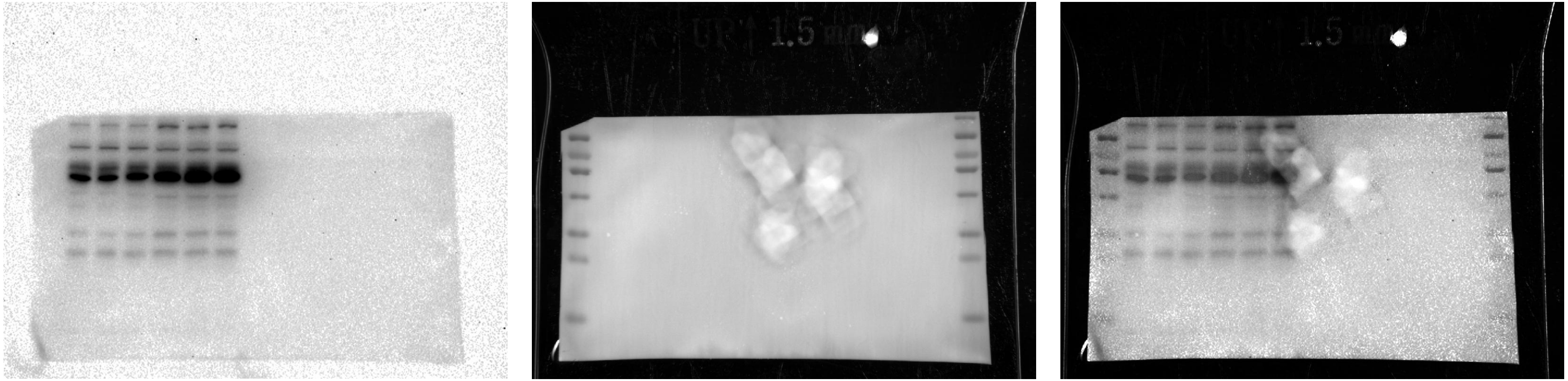

GSDMD-N

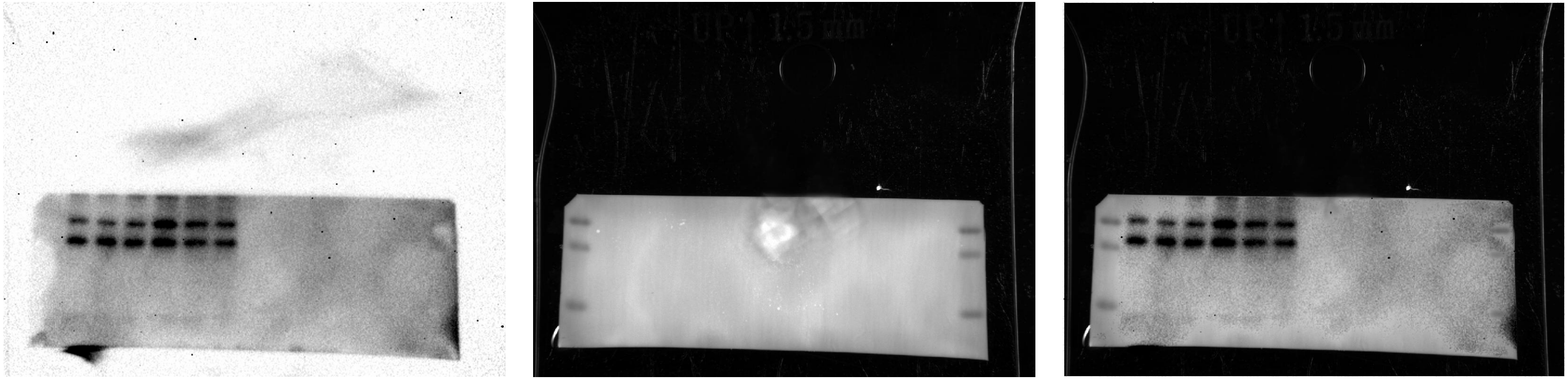

Cleaved cas3

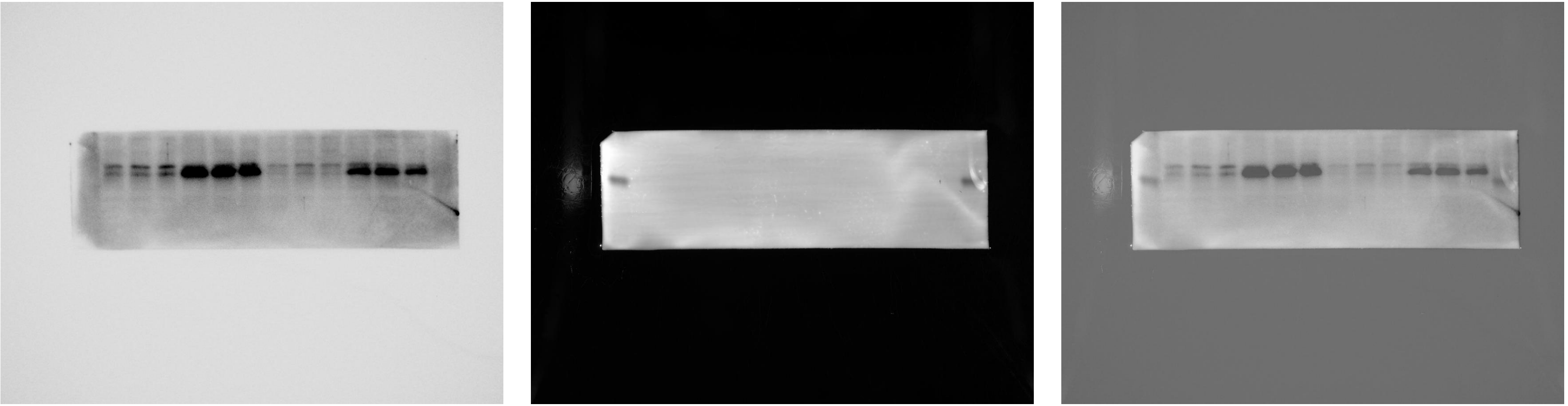

BAX

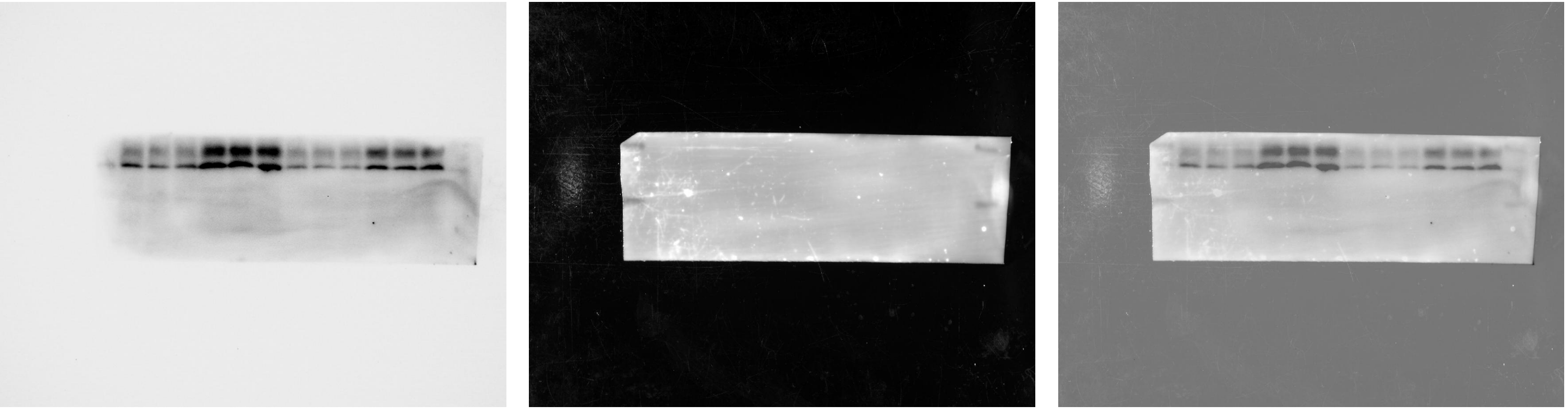

β-actin

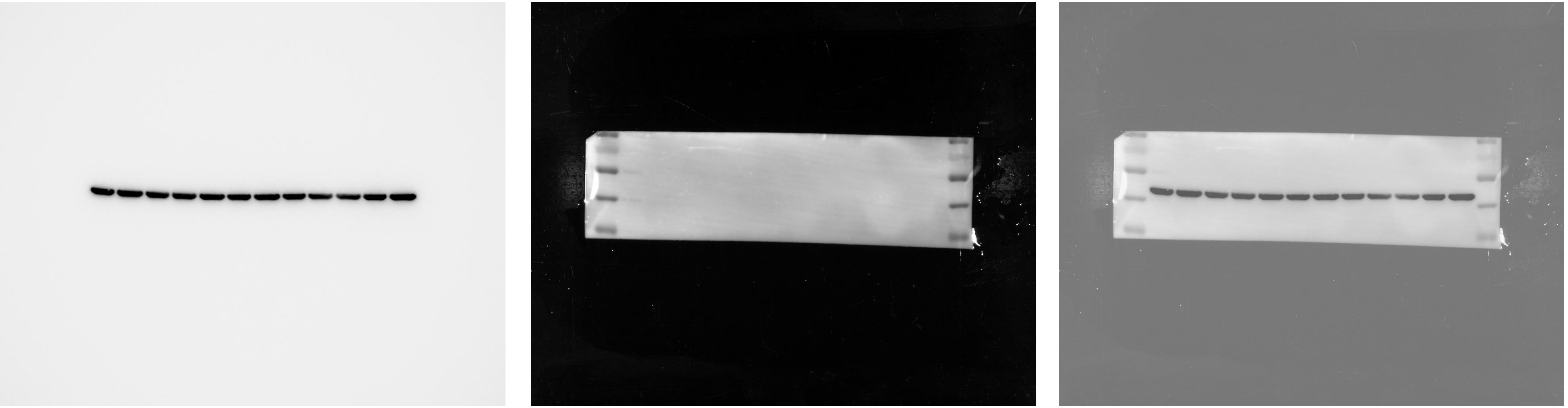

Figure 3

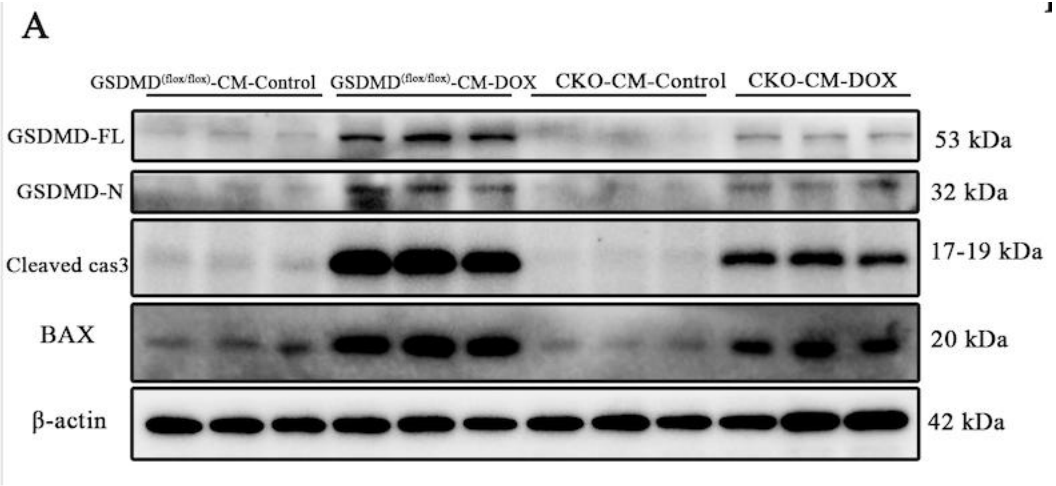
A
GSDMD(flox/flox)-CM-Control
GSDMD(flox/flox)-CM-DOX
CKO-CM-Control
CKO-CM-DOX
GSDMD-FL
GSDMD-N
Cleaved cas3
BAX
β-actin
53 kDa
32 kDa
17-19 kDa
20 kDa
42 kDa

GSDMD-FL

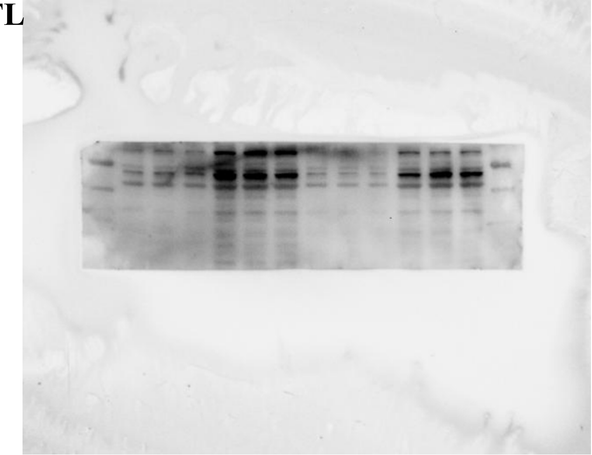

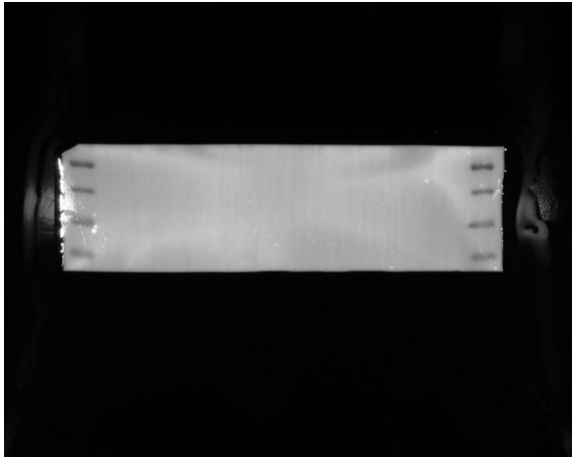

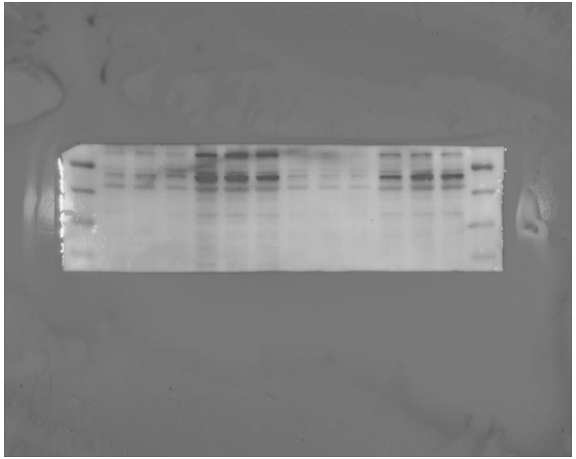

GSDMD-N

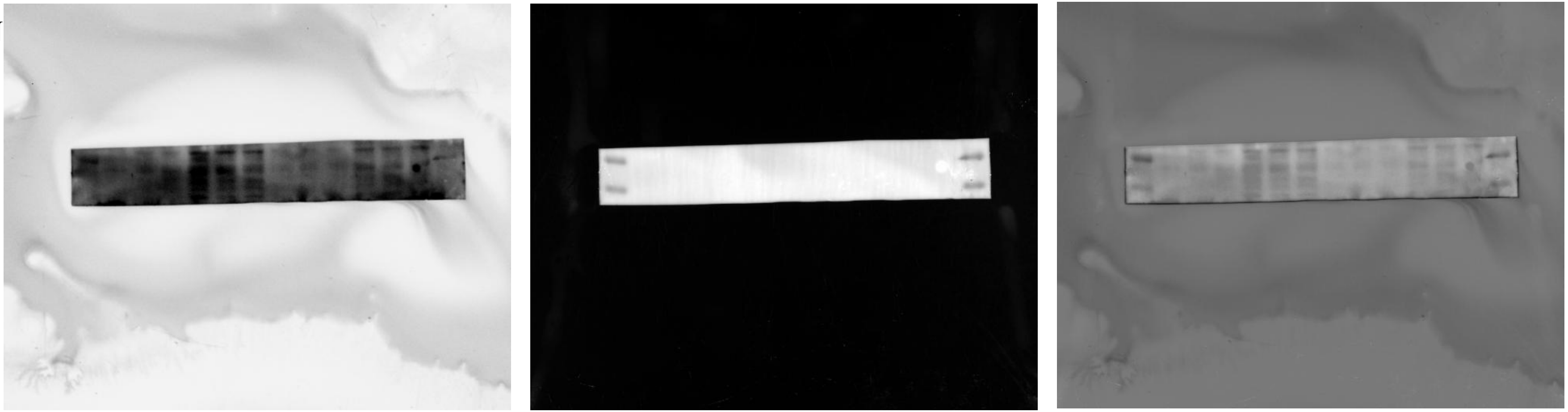

CC3

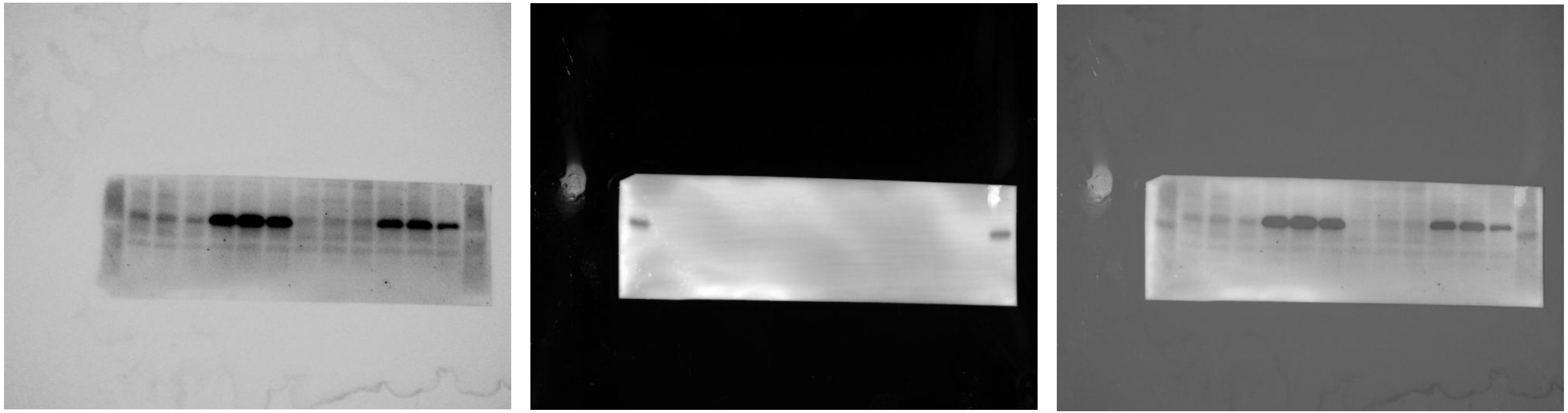

BAX

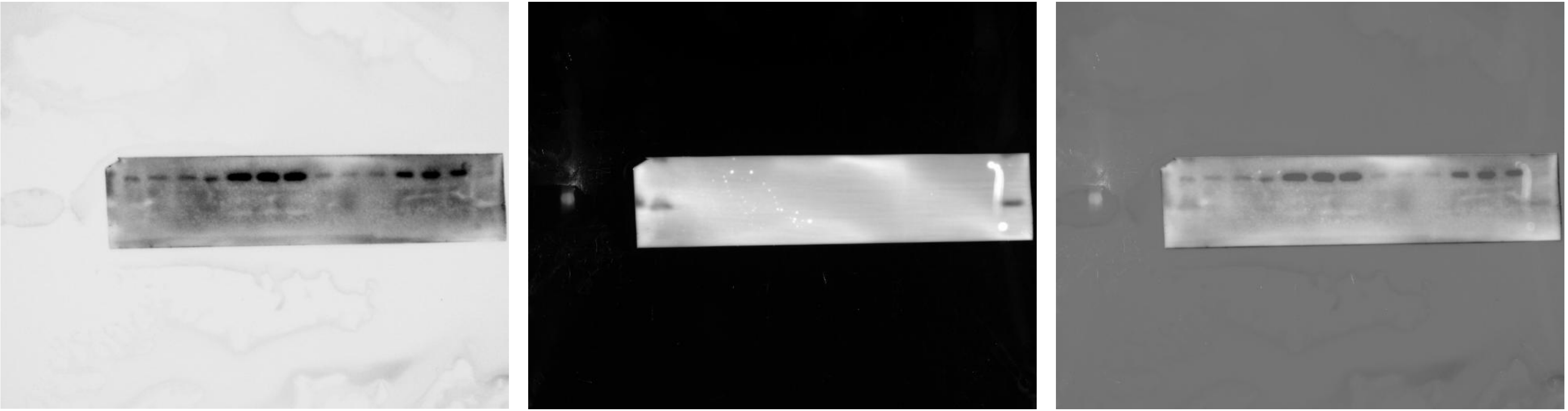

β-actin

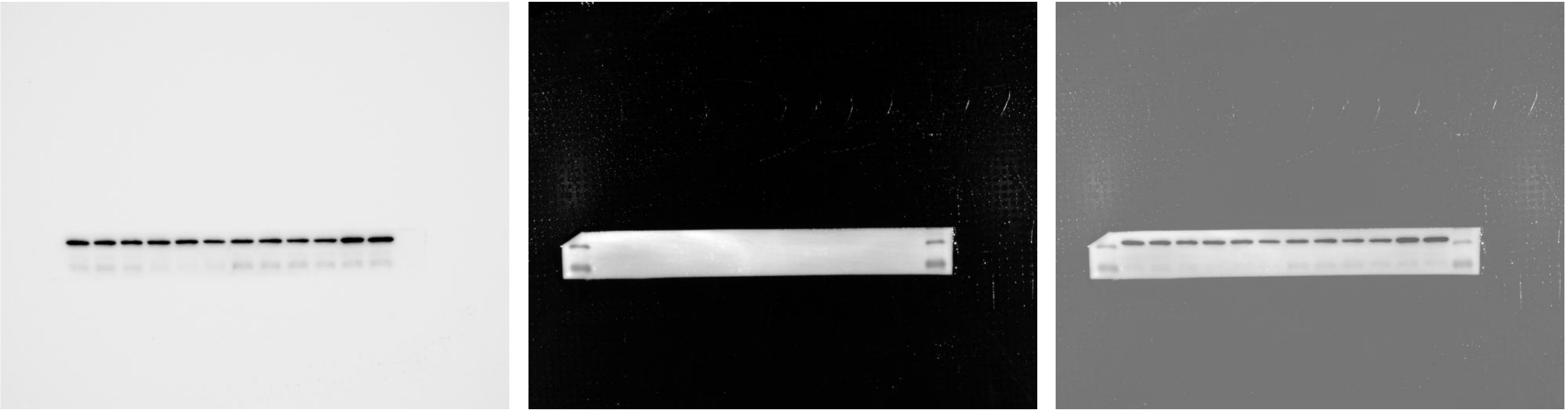

E

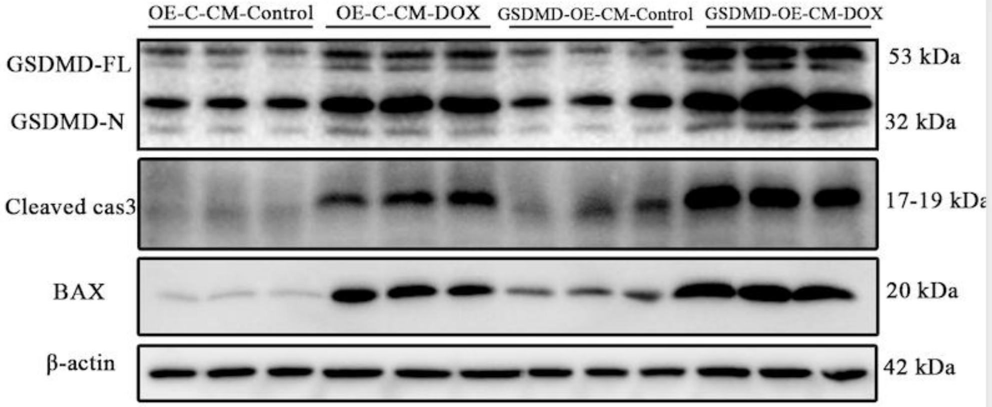
OE-C-CM-Control
OE-C-CM-DOX
GSDMD-OE-CM-Control
GSDMD-OE-CM-DOX
GSDMD-FL
GSDMD-N
Cleaved cas3
BAX
β-actin
53 kDa
32 kDa
17-19 kDa
20 kDa
42 kDa

GSDMD

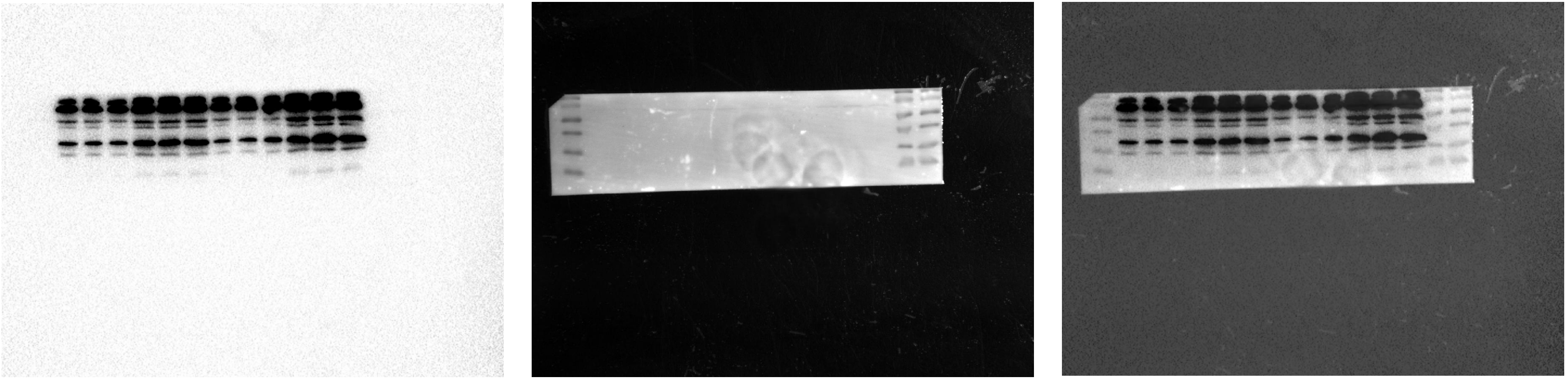

CC3

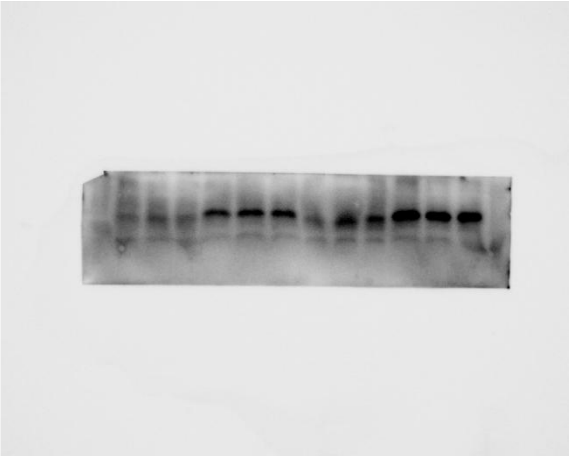

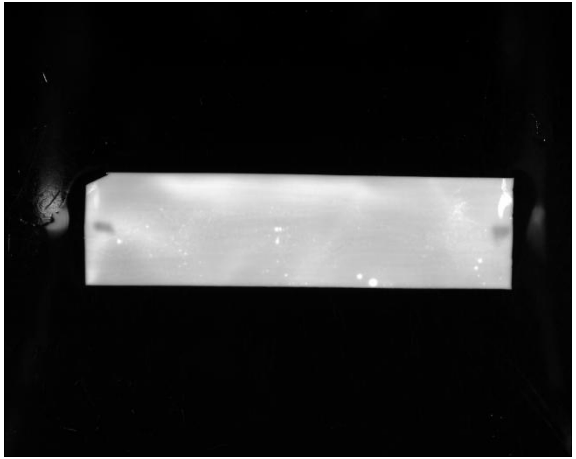

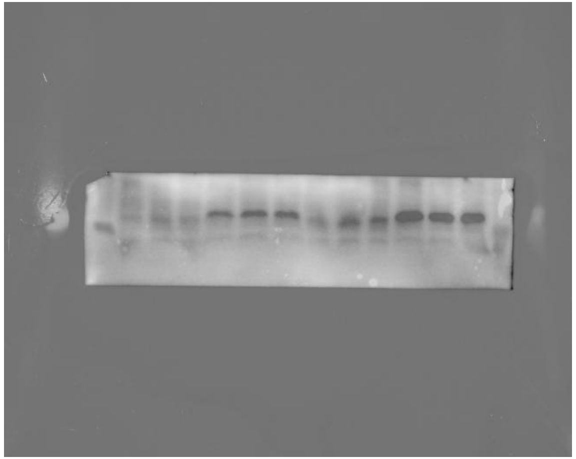

BAX

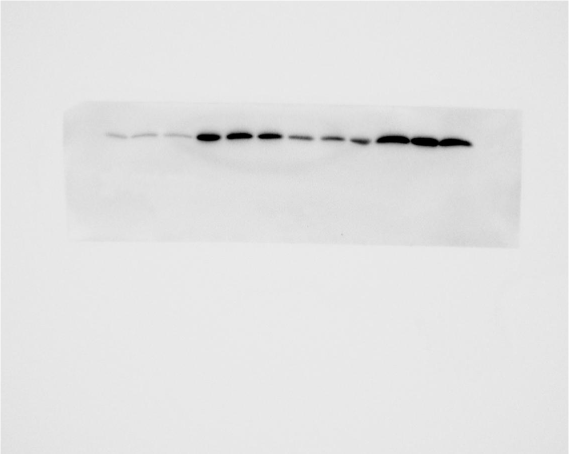

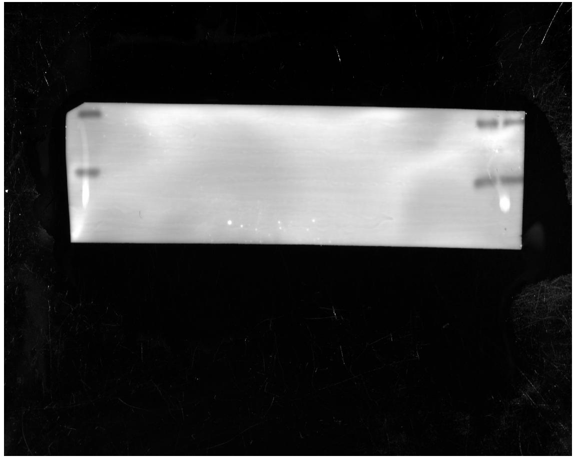

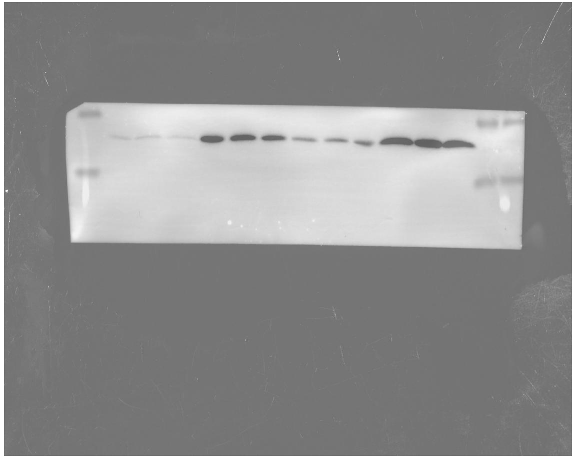

β-actin

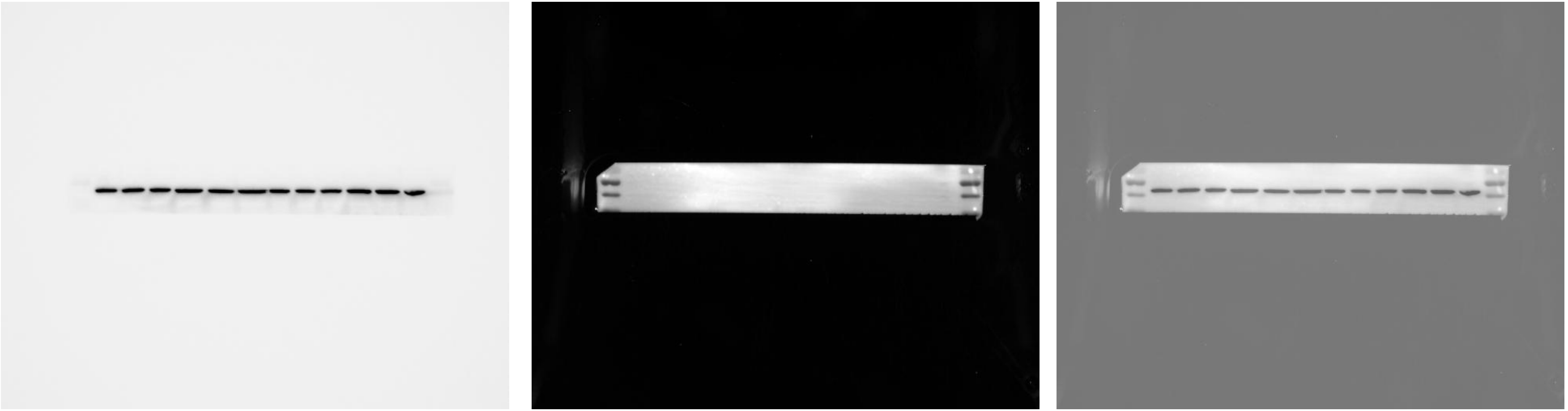

Figure 4

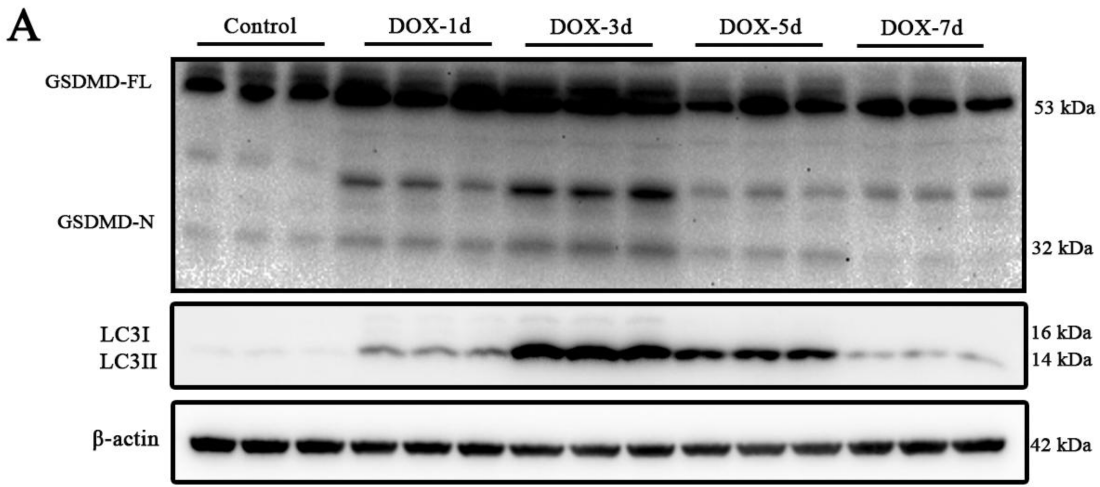
A
Control
DOX-1d
DOX-3d
DOX-5d
DOX-7d
GSDMD-FL
GSDMD-N
53 kDa
32 kDa
LC3I
LC3II
16 kDa
14 kDa
β-actin
42 kDa

GSDMD

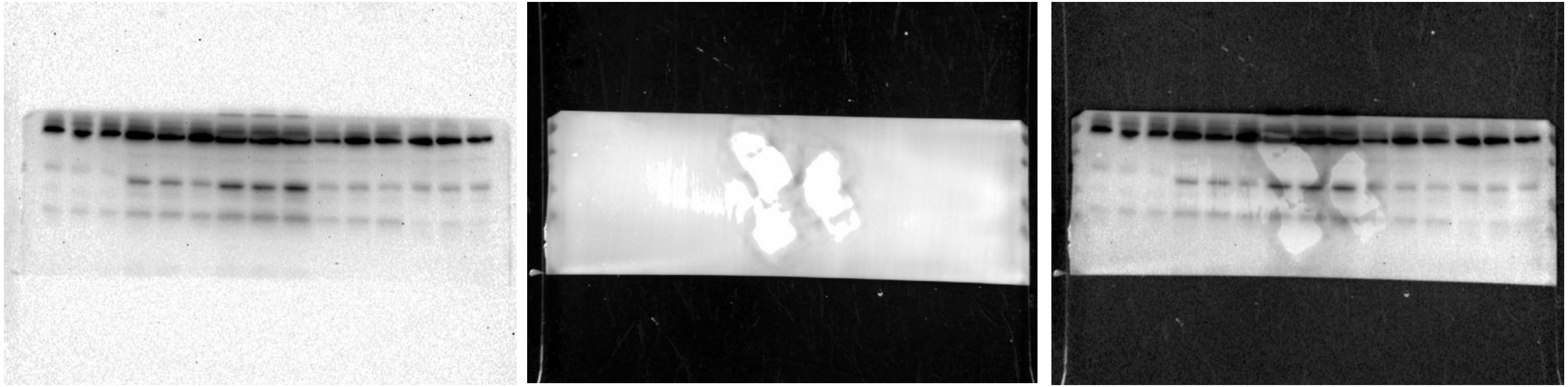

LC3

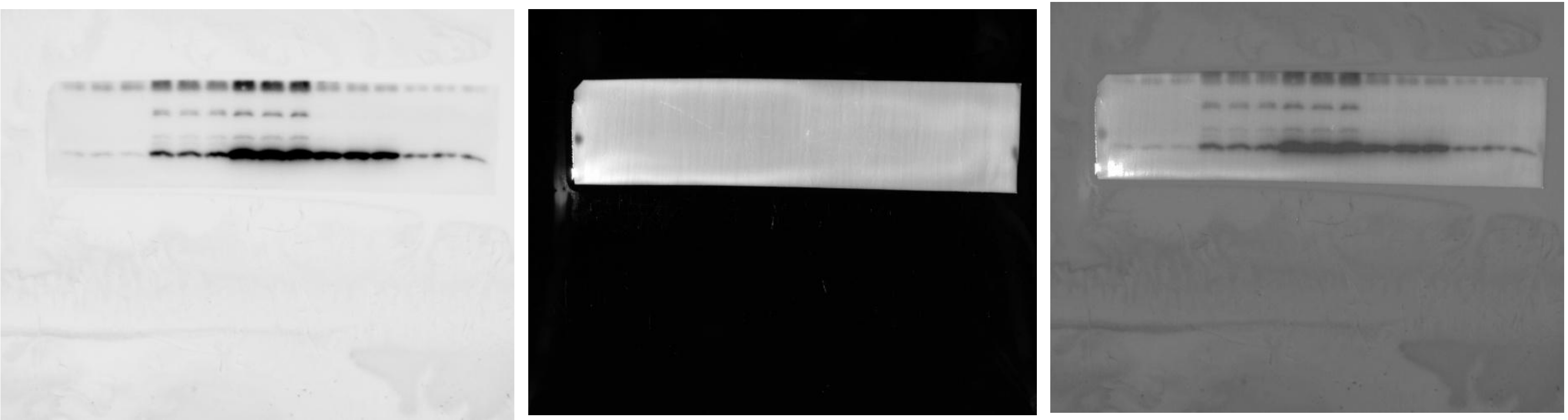

β-actin

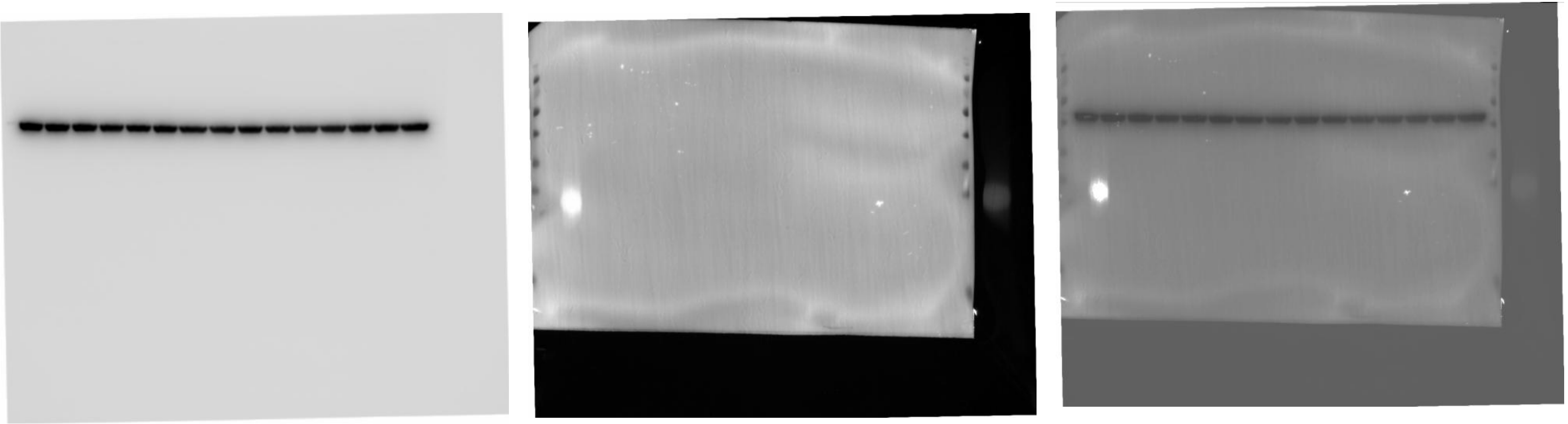

D

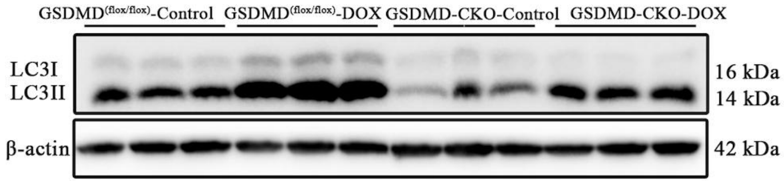
GSDMD(flox/flox)-Control
GSDMD(flox/flox)-DOX
GSDMD-CKO-Control
GSDMD-CKO-DOX
LC3I
LC3II
β-actin
16 kDa
14 kDa
42 kDa

LC3

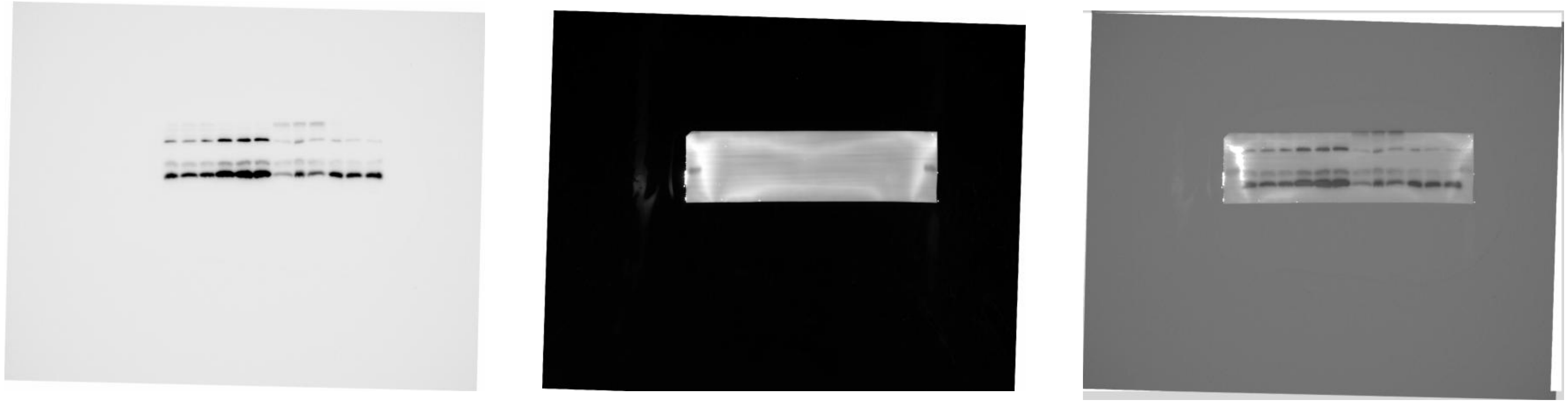

β-actin

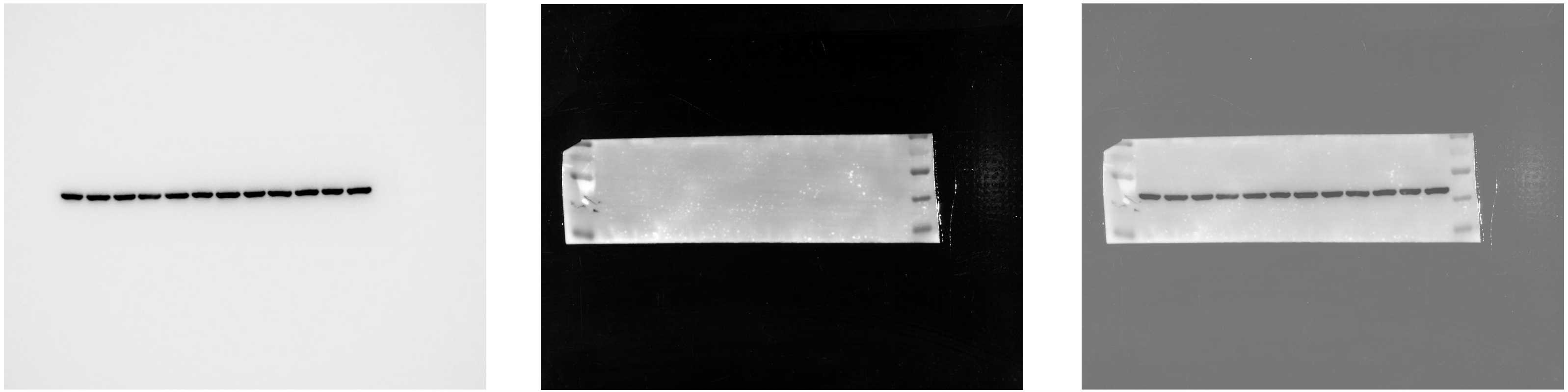

E

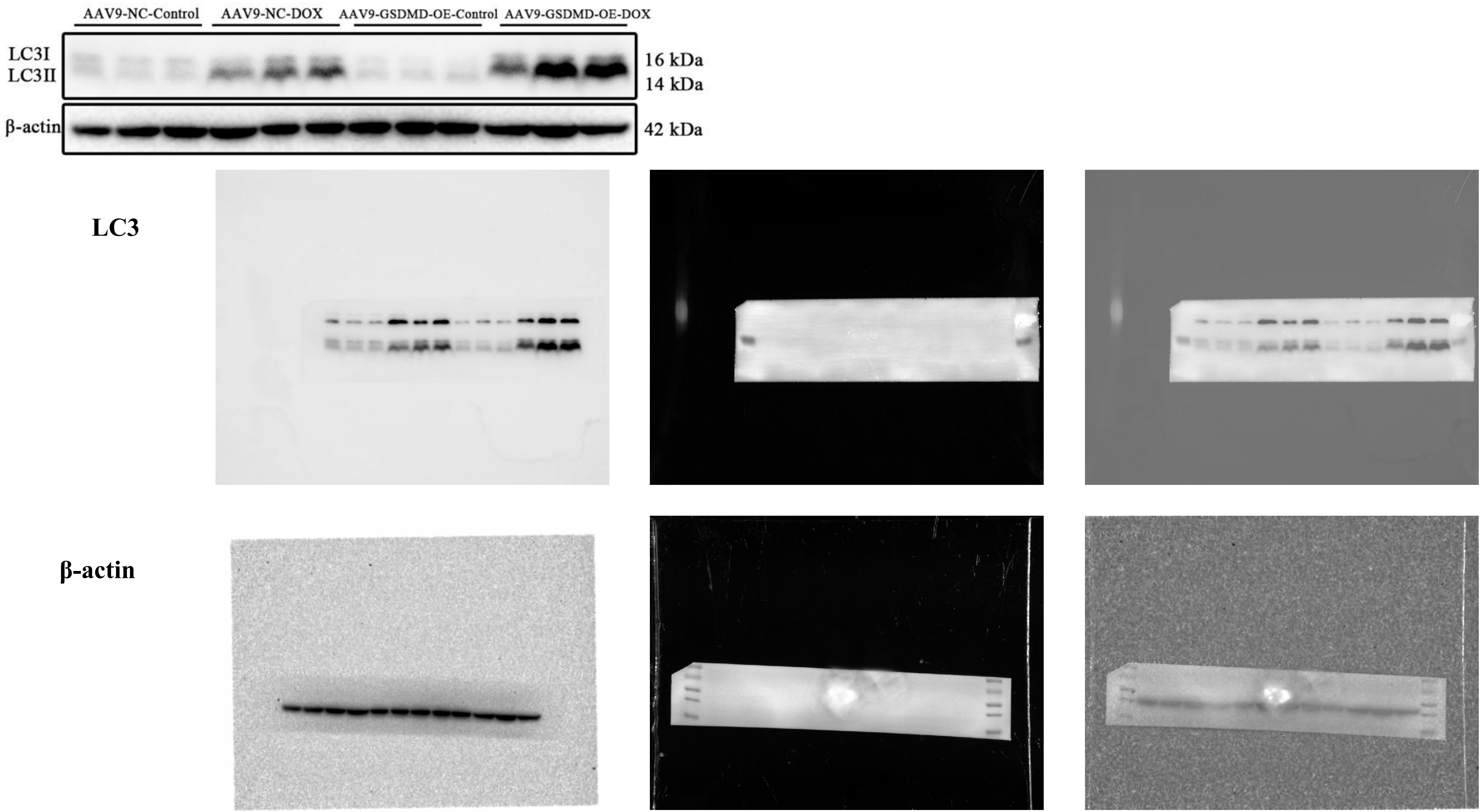
AAV9-NC-Control
AAV9-NC-DOX
AAV9-GSDMD-OE-Control
AAV9-GSDMD-OE-DOX
LC3I
LC3II
16 kDa
14 kDa
β-actin
42 kDa
LC3
β-actin

F

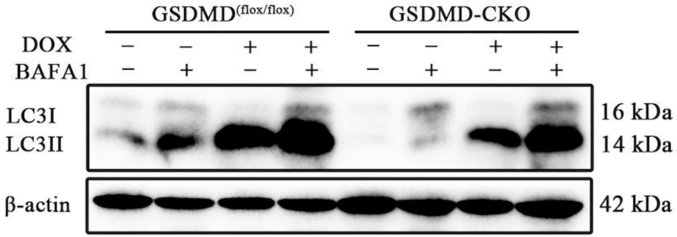

GSDMD(flox/flox)
GSDMD-CKO
DOX − − + + − − + +
BAFA1 − + − + − + − +
LC3I
LC3II
16 kDa
14 kDa
β-actin
42 kDa

LC3

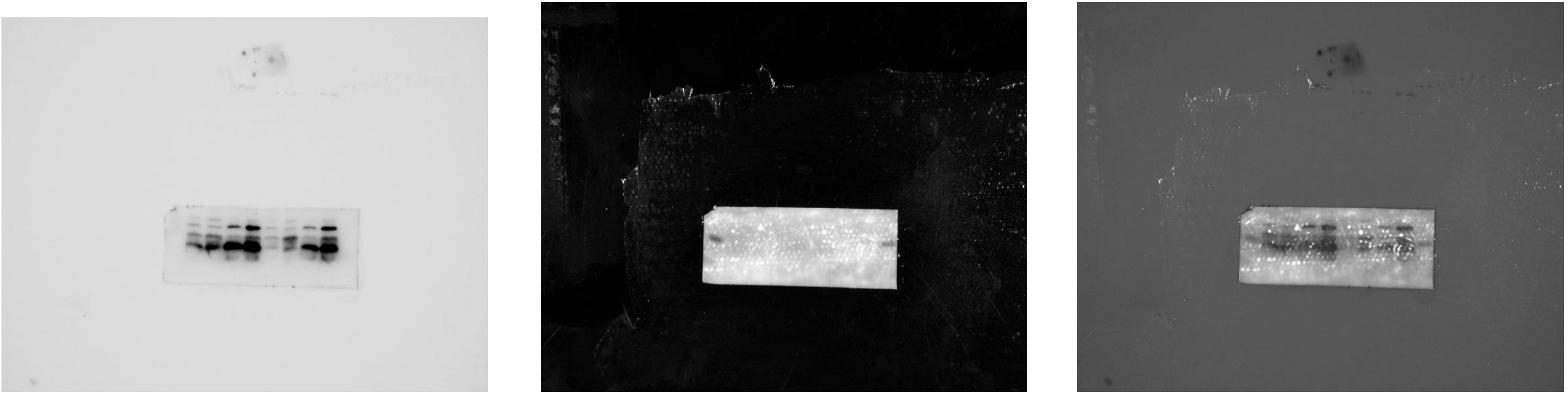

β-actin

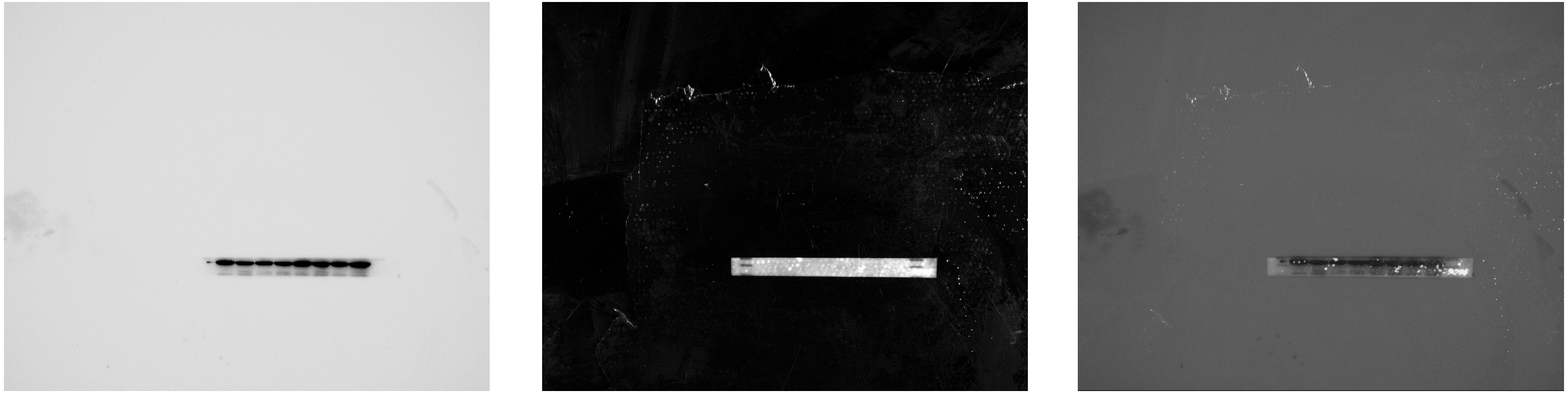

G

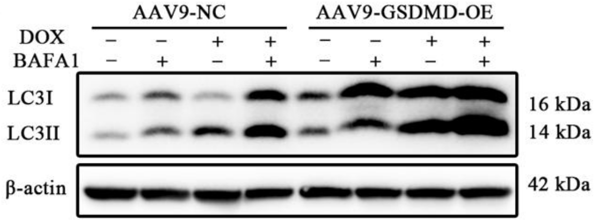
AAV9-NC
AAV9-GSDMD-OE
DOX − − + + − − + +
BAFA1 − + − + − + − +
LC3I
LC3II
16 kDa
14 kDa
β-actin
42 kDa

LC3

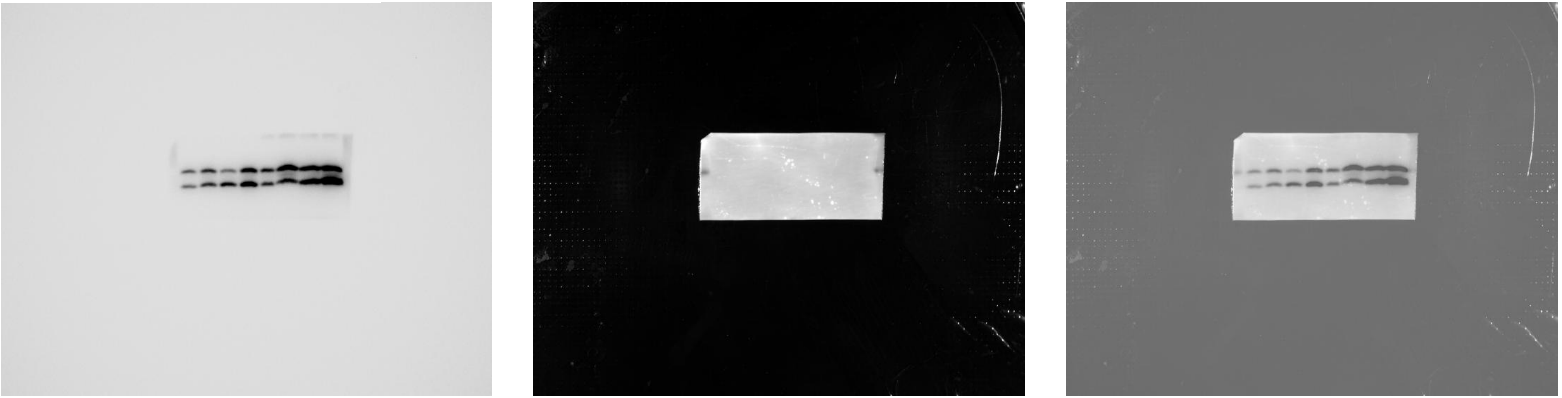

β-actin

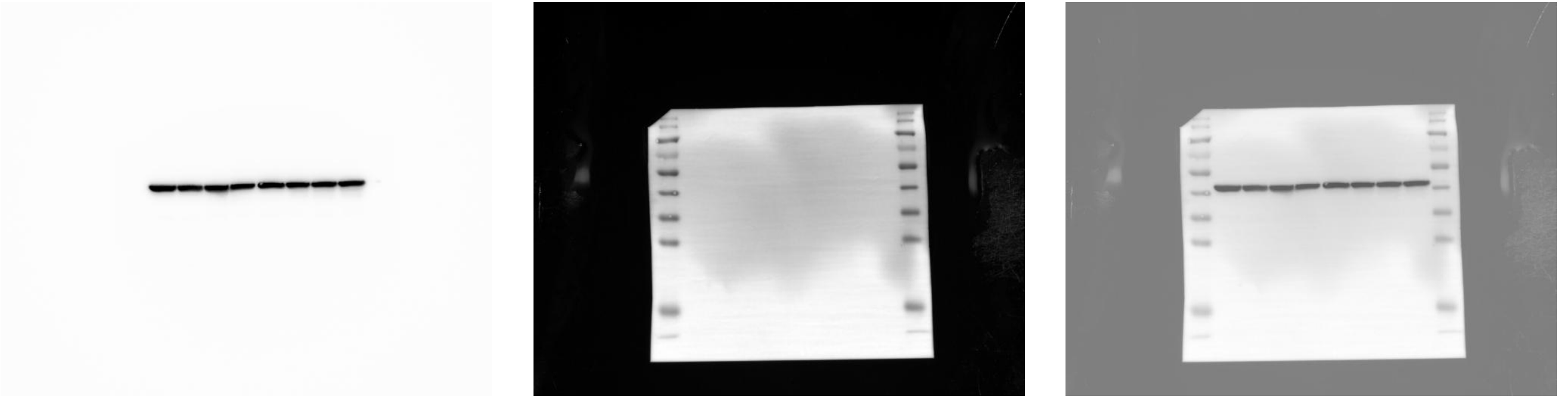

Figure 5

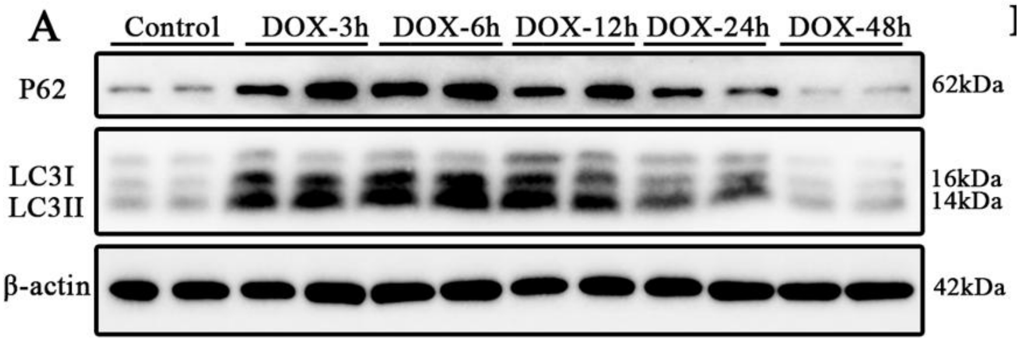
A
Control
DOX-3h
DOX-6h
DOX-12h
DOX-24h
DOX-48h
P62
62kDa
LC3I
LC3II
16kDa
14kDa
β-actin
42kDa

P62

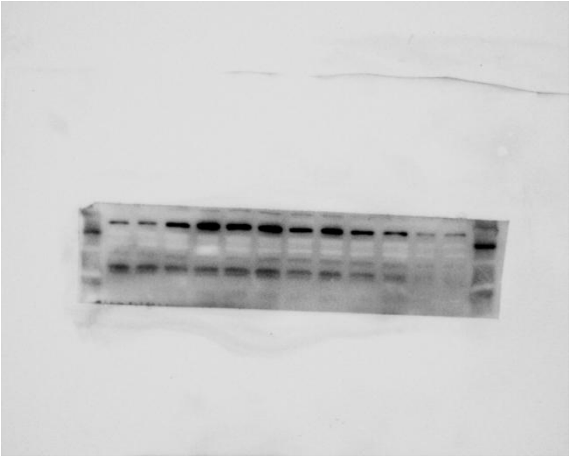

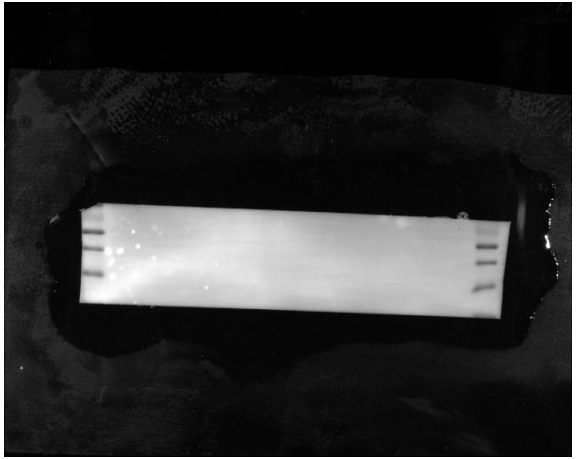

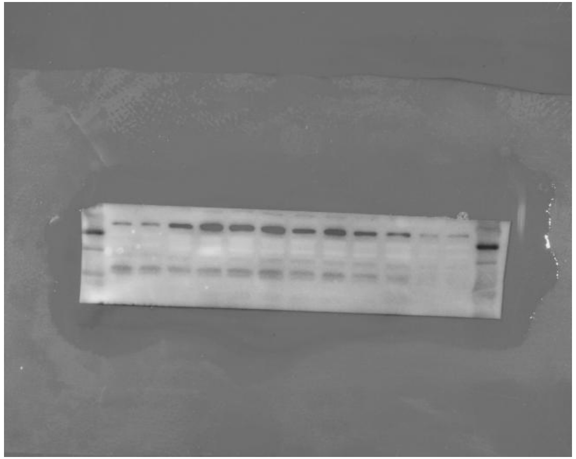

LC3

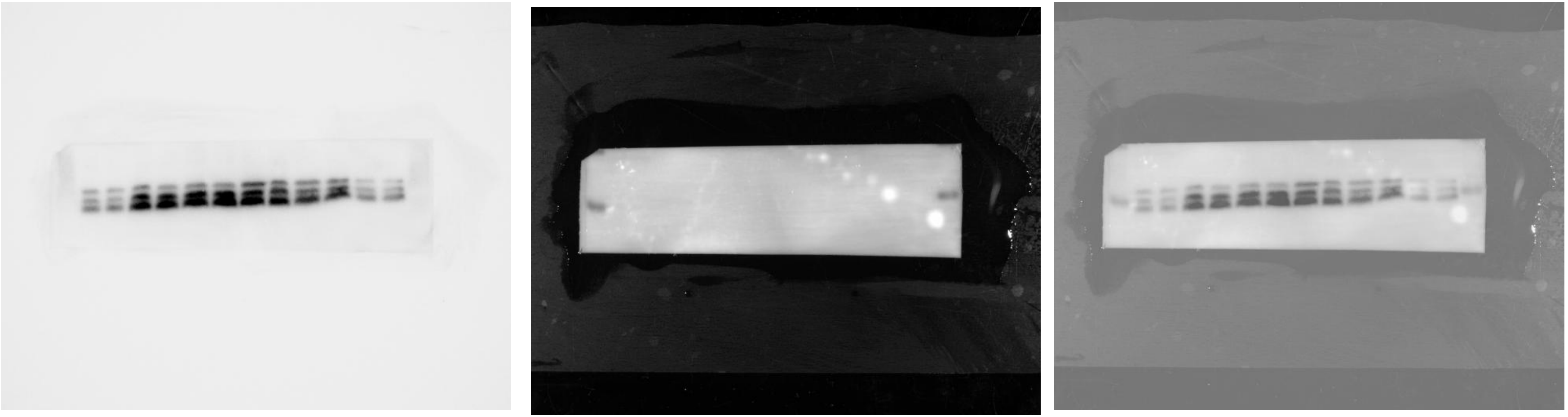

β-actin

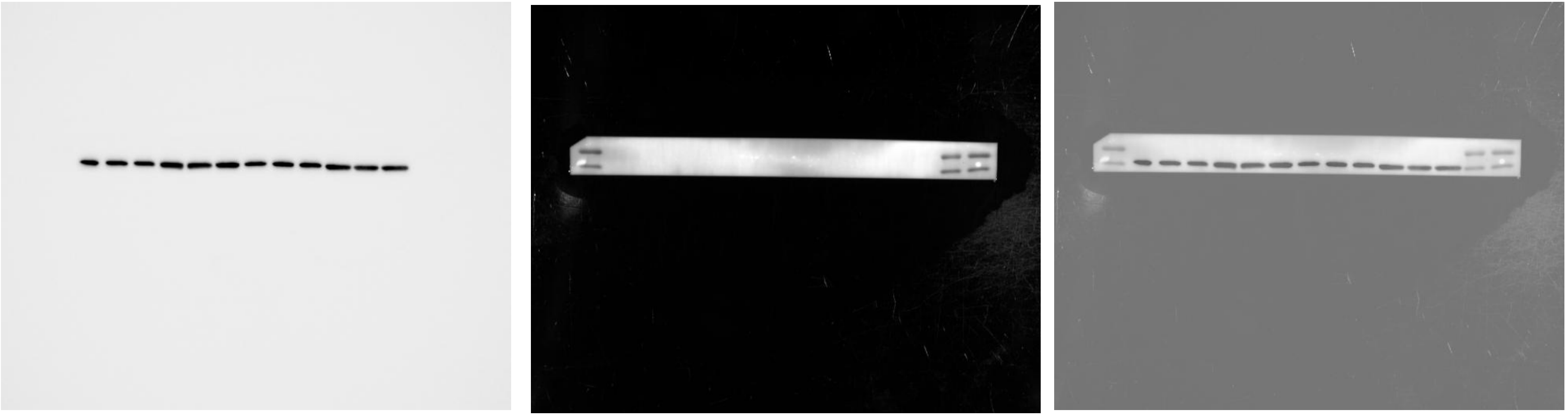

D

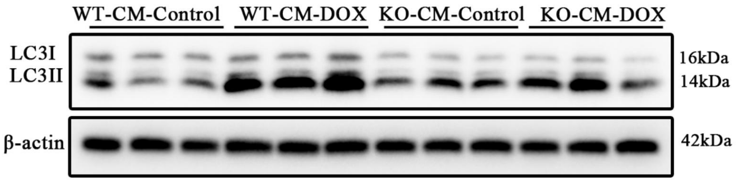
WT-CM-Control
WT-CM-DOX
KO-CM-Control
KO-CM-DOX
LC3I
LC3II
16kDa
14kDa
β-actin
42kDa

LC3

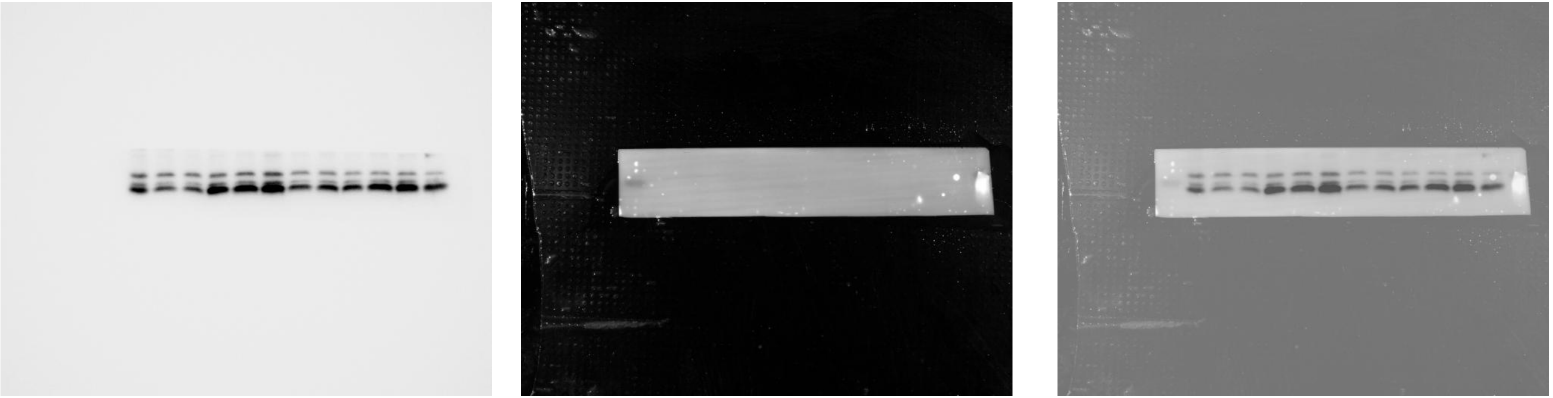

β-actin

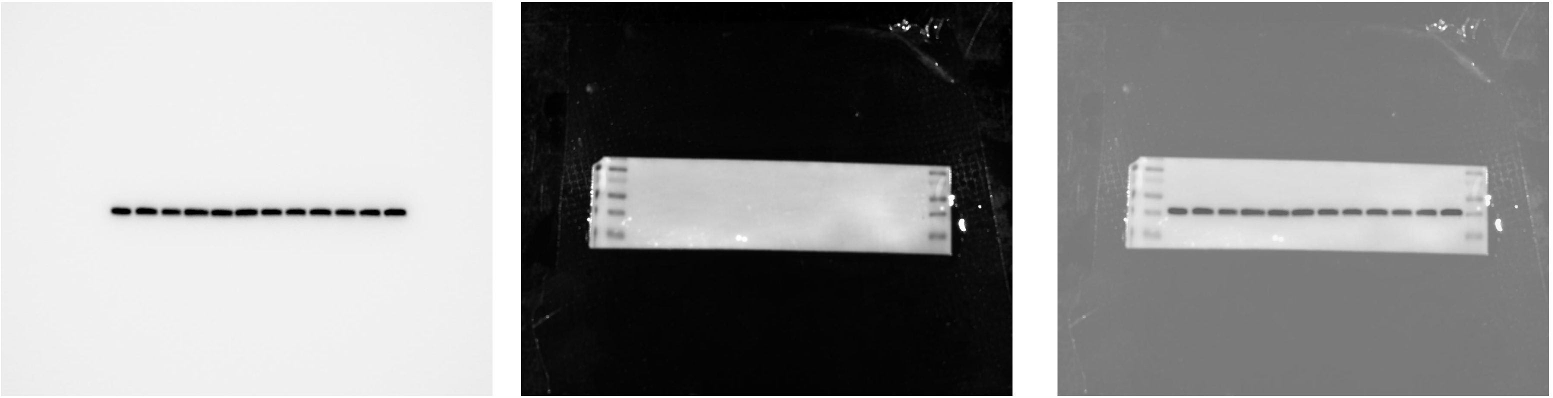

E

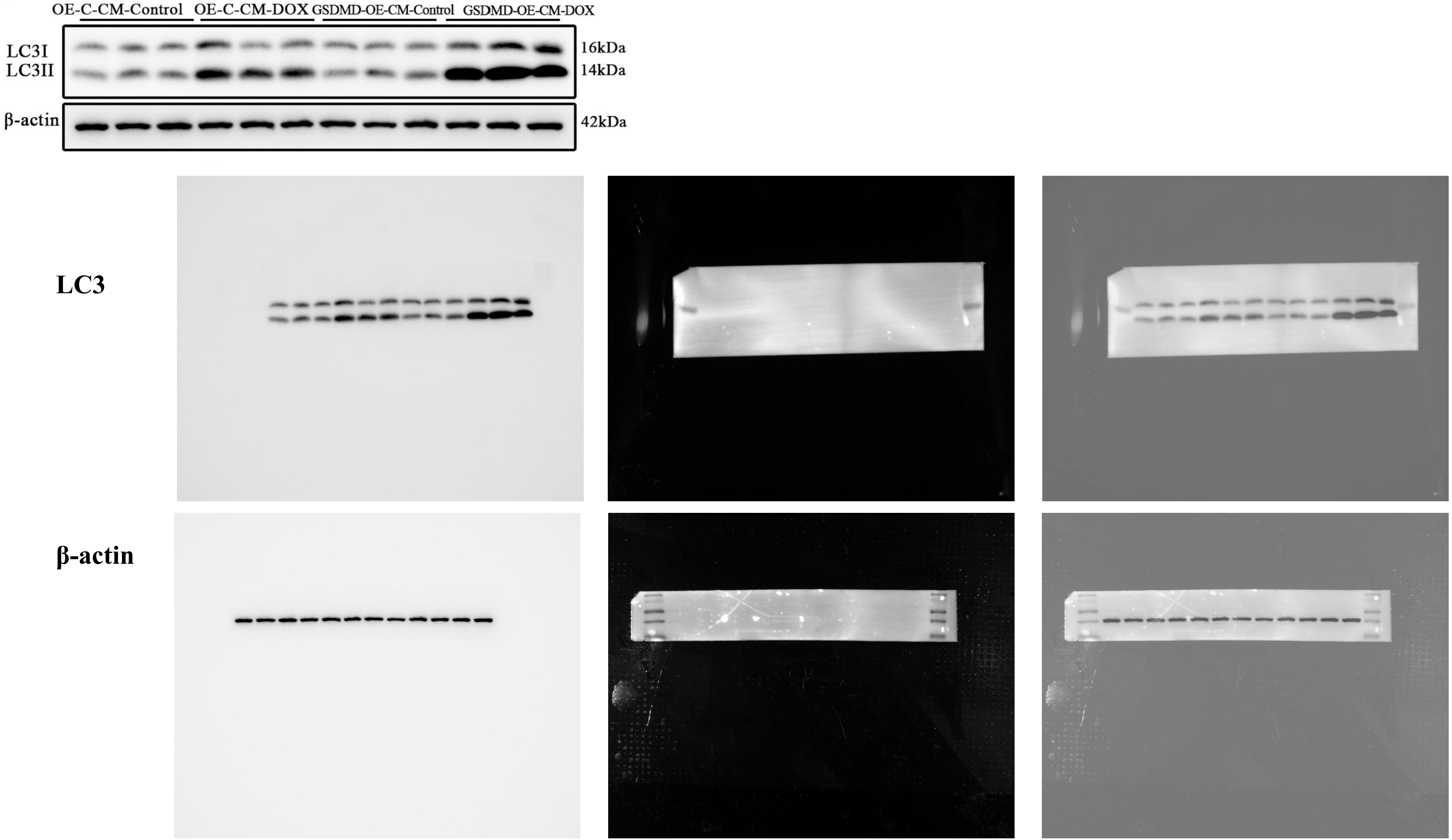
OE-C-CM-Control
OE-C-CM-DOX
GSDMD-OE-CM-Control
GSDMD-OE-CM-DOX
LC3I
LC3II
16kDa
14kDa
β-actin
42kDa
LC3
β-actin

I

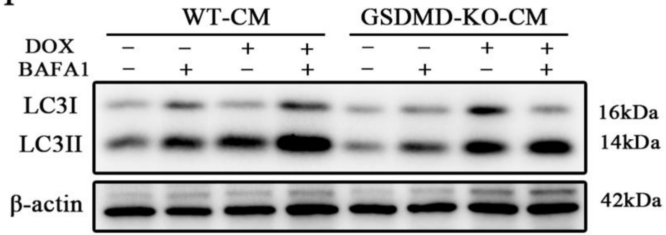
WT-CM
GSDMD-KO-CM
DOX − − + + − − + +
BAFA1 − + − + − + − +
LC3I
LC3II
16kDa
14kDa
β-actin
42kDa

LC3

β-actin

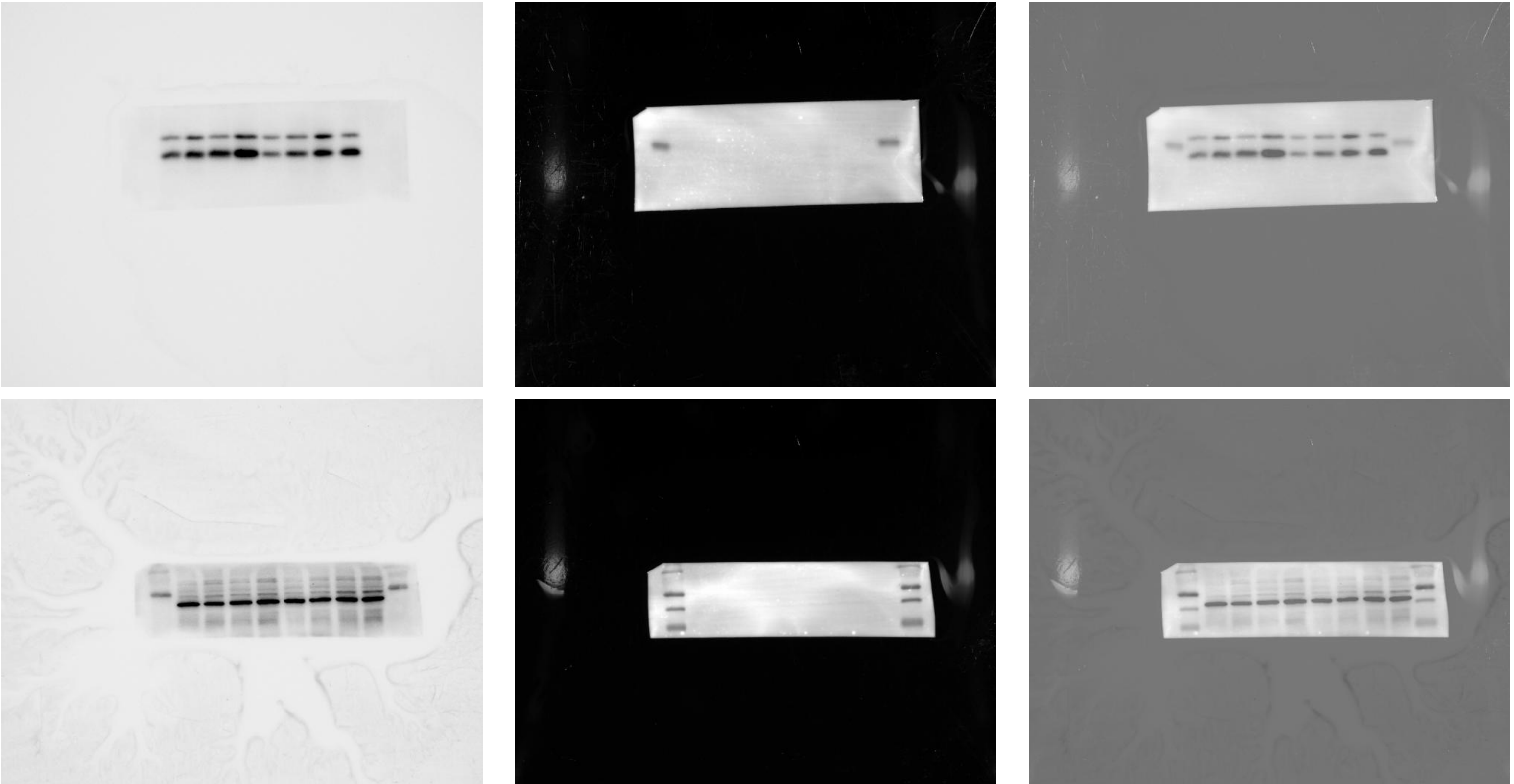

J

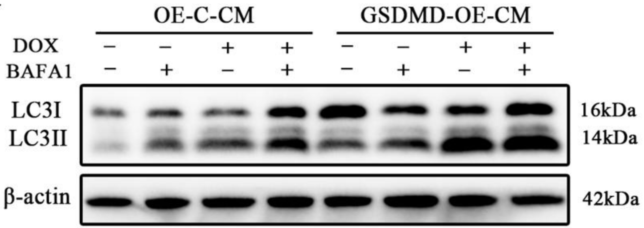
OE-C-CM
GSDMD-OE-CM
DOX
BAFA1
LC3I
LC3II
16kDa
14kDa
β-actin
42kDa

LC3

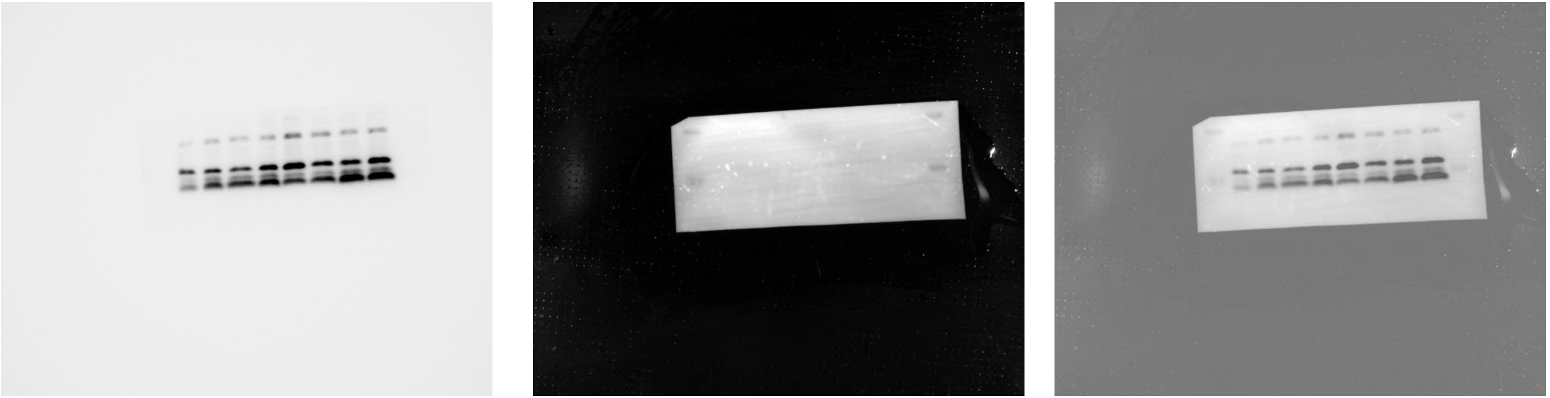

β-actin

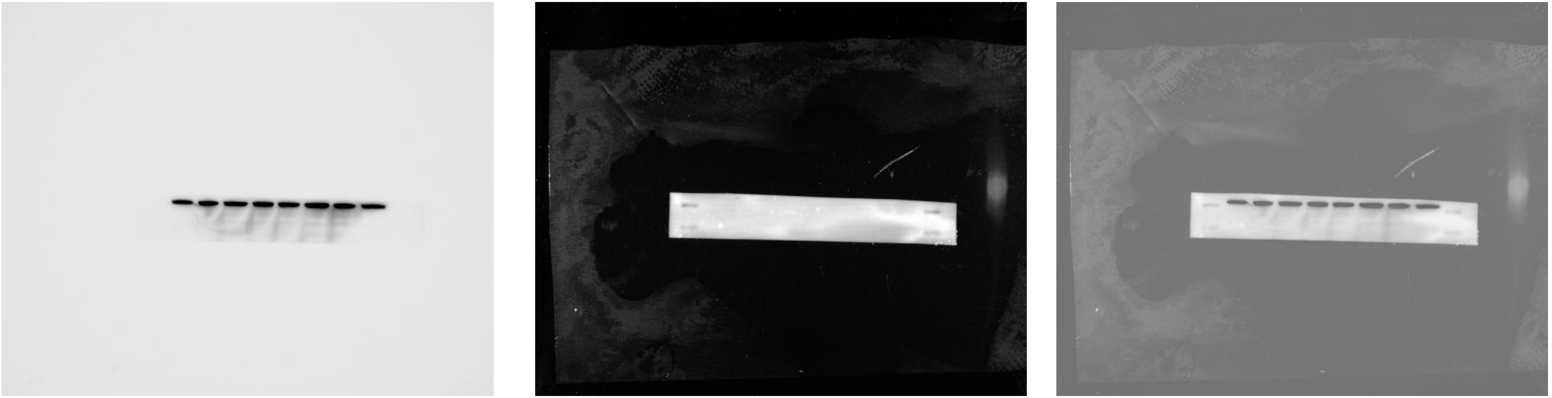

Figure 6

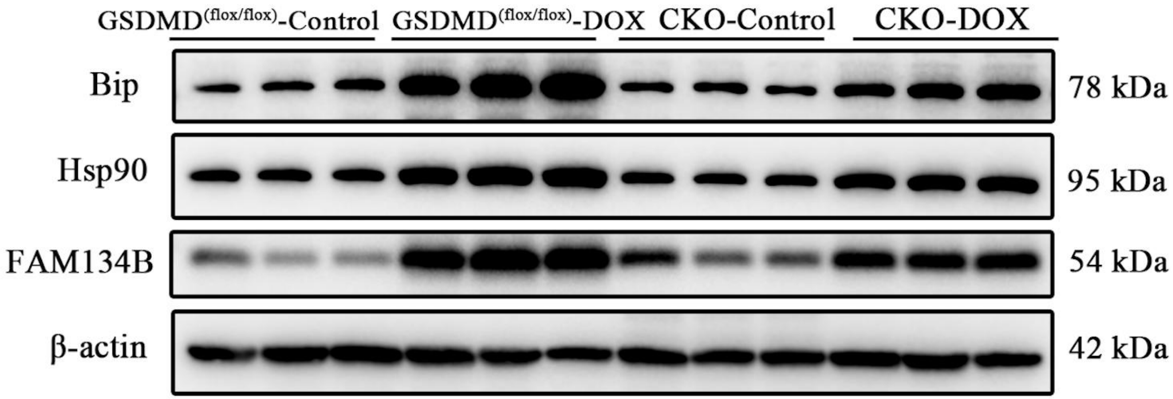
GSDMD(flox/flox)-Control
GSDMD(flox/flox)-DOX
CKO-Control
CKO-DOX
Bip
Hsp90
FAM134B
β-actin
78 kDa
95 kDa
54 kDa
42 kDa

Bip

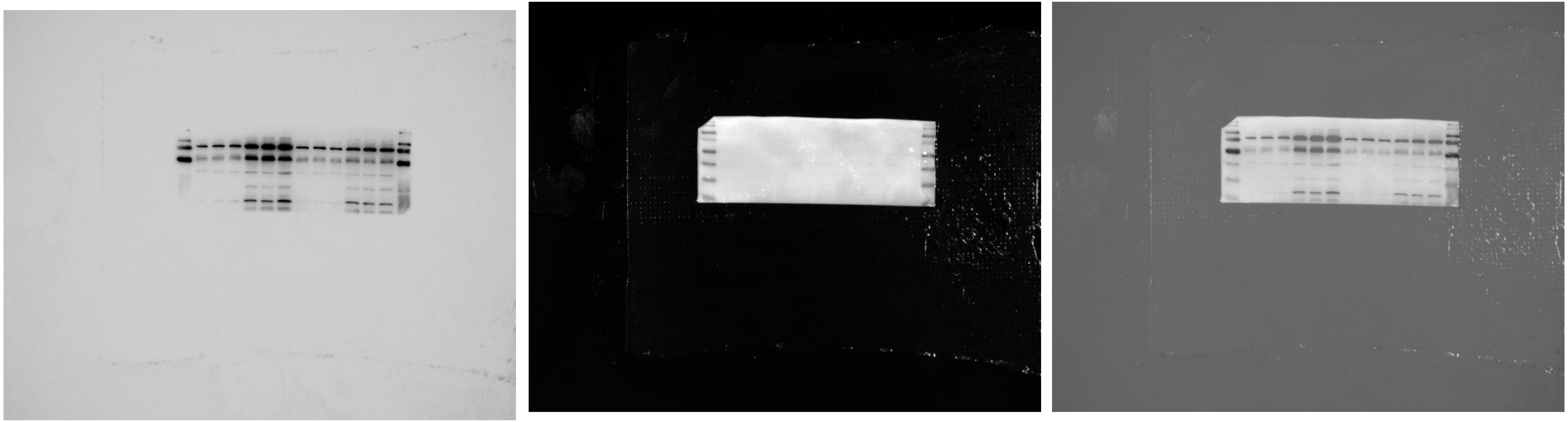

Hsp90

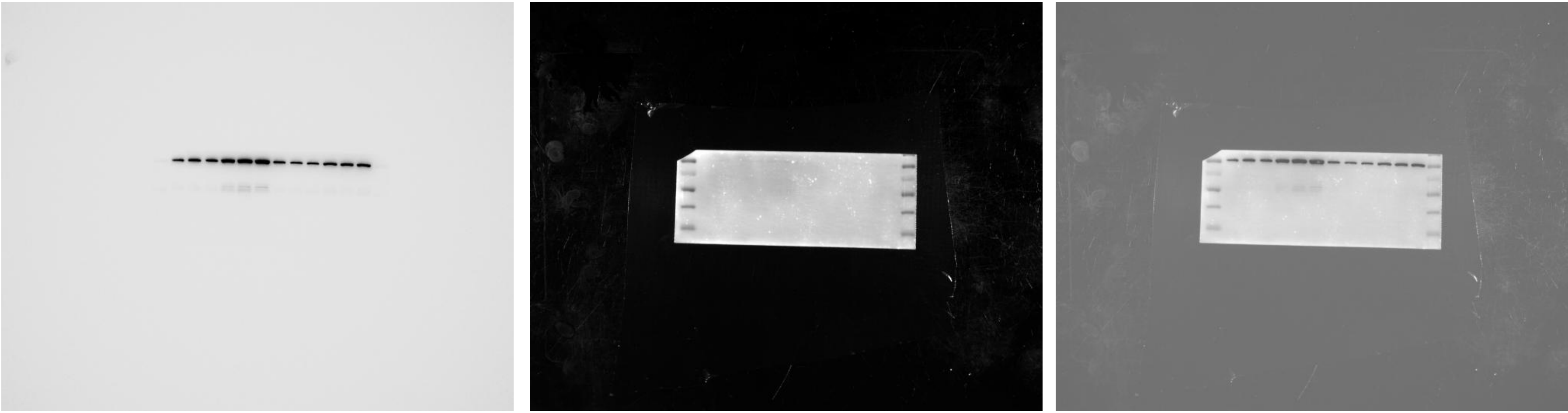

FAM134B

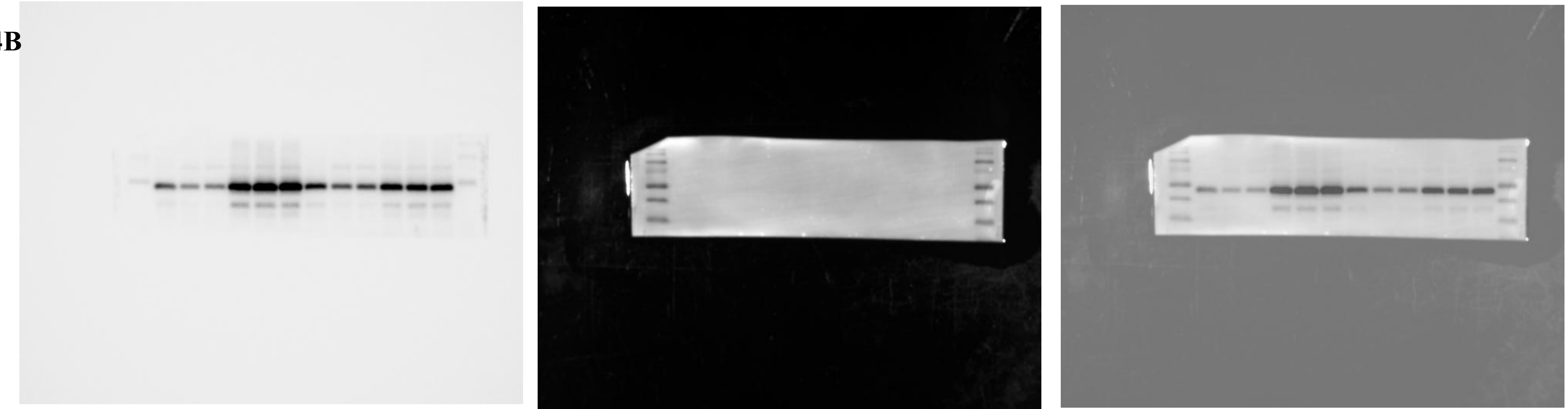

β-actin

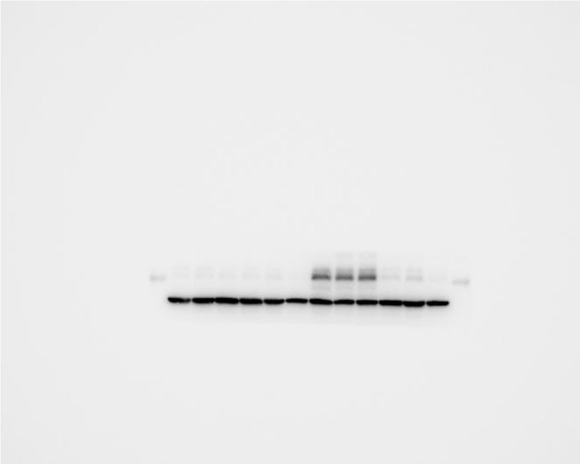

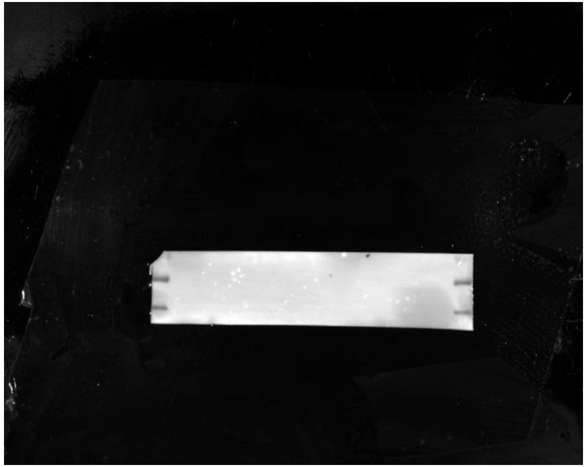

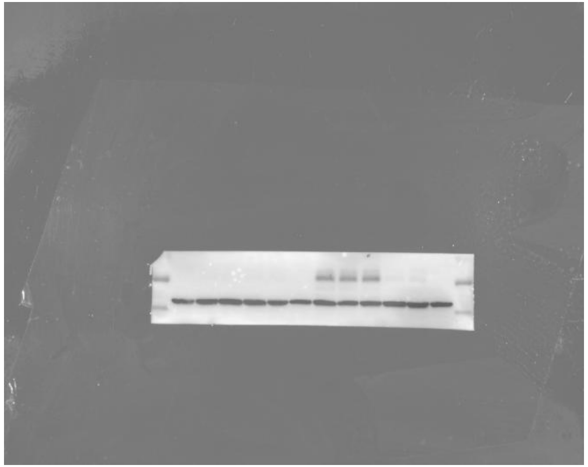

Figure 7

D

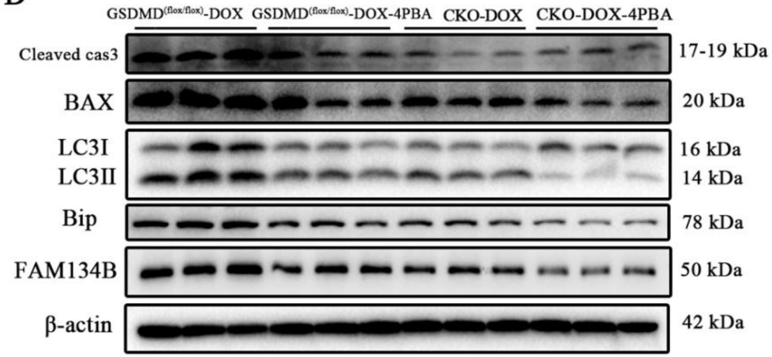
GSDMD(flox/flox)-DOX
GSDMD(flox/flox)-DOX-4PBA
CKO-DOX
CKO-DOX-4PBA
Cleaved cas3
BAX
LC3I
LC3II
Bip
FAM134B
β-actin
17-19 kDa
20 kDa
16 kDa
14 kDa
78 kDa
50 kDa
42 kDa

CC3

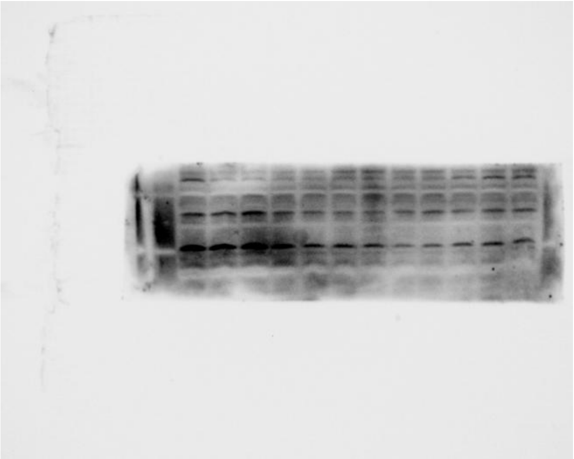

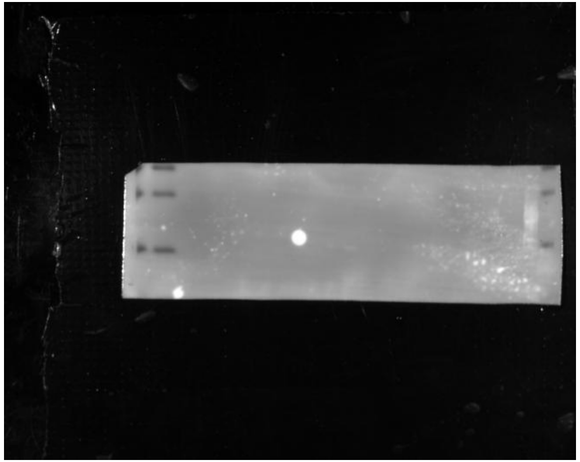

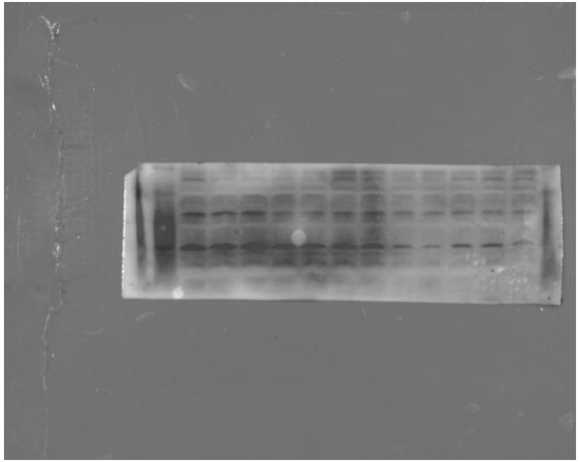

BAX

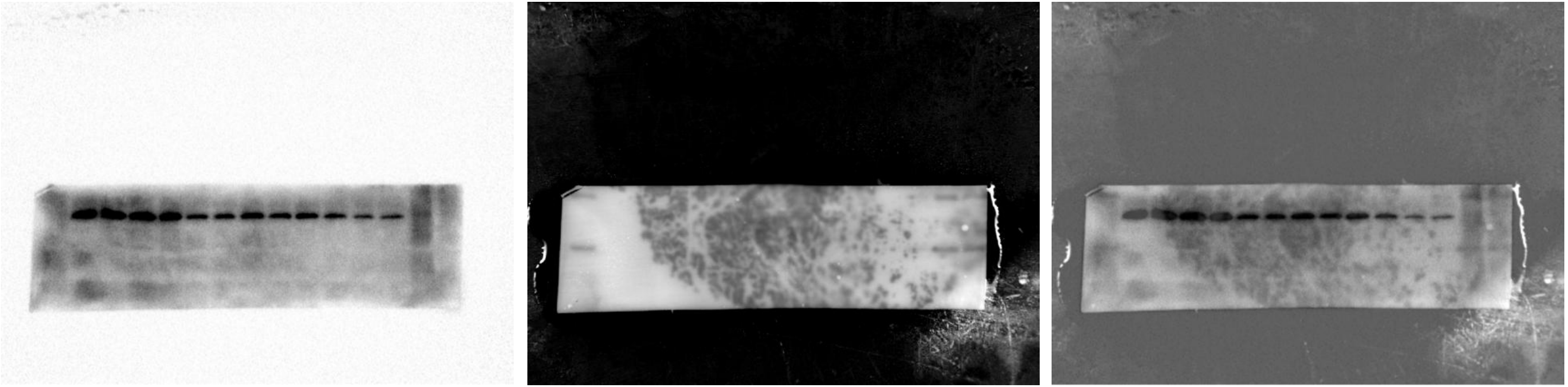

LC3

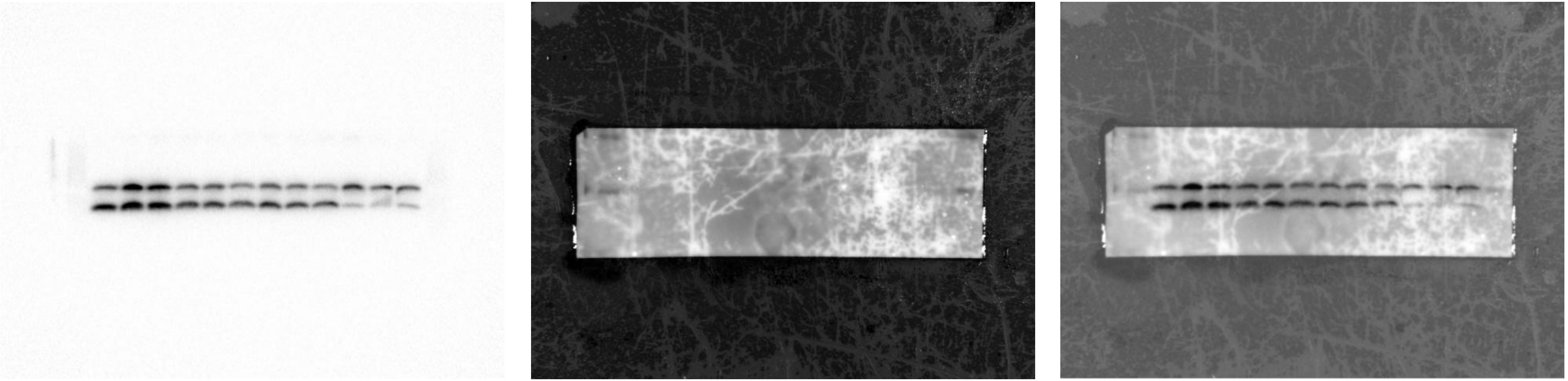

BIP

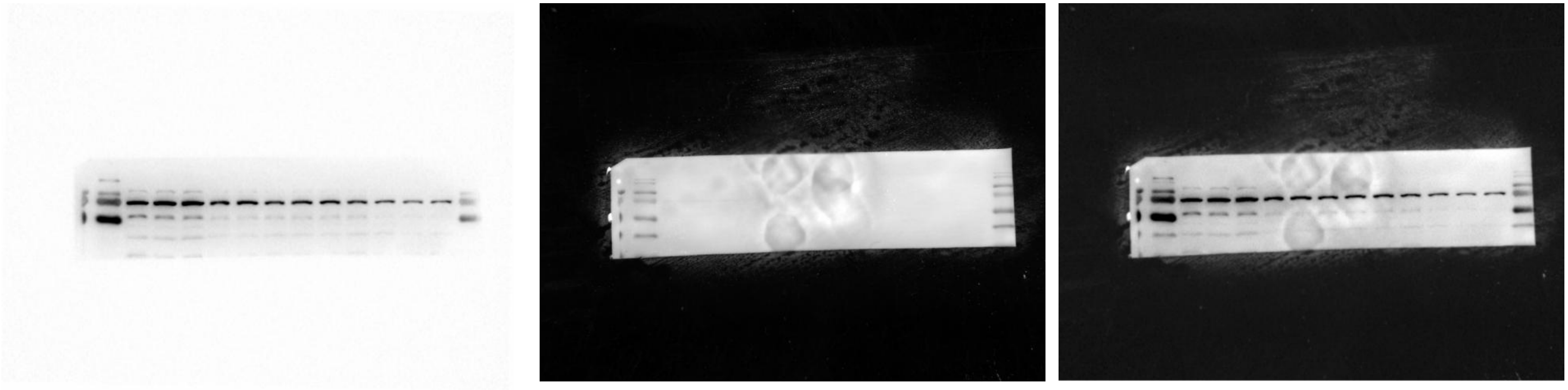

FAM134B

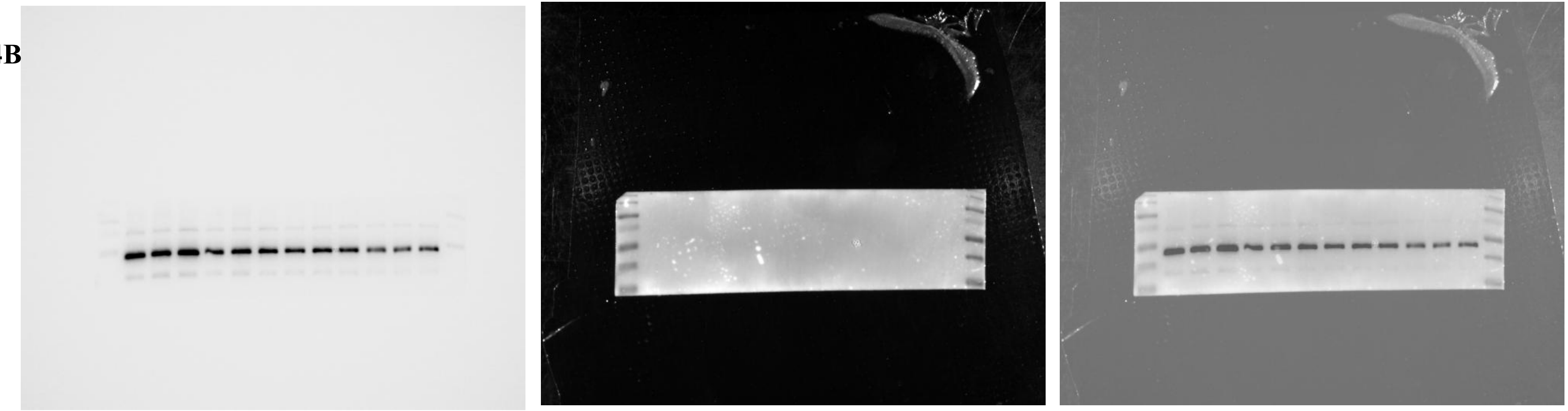

β-actin

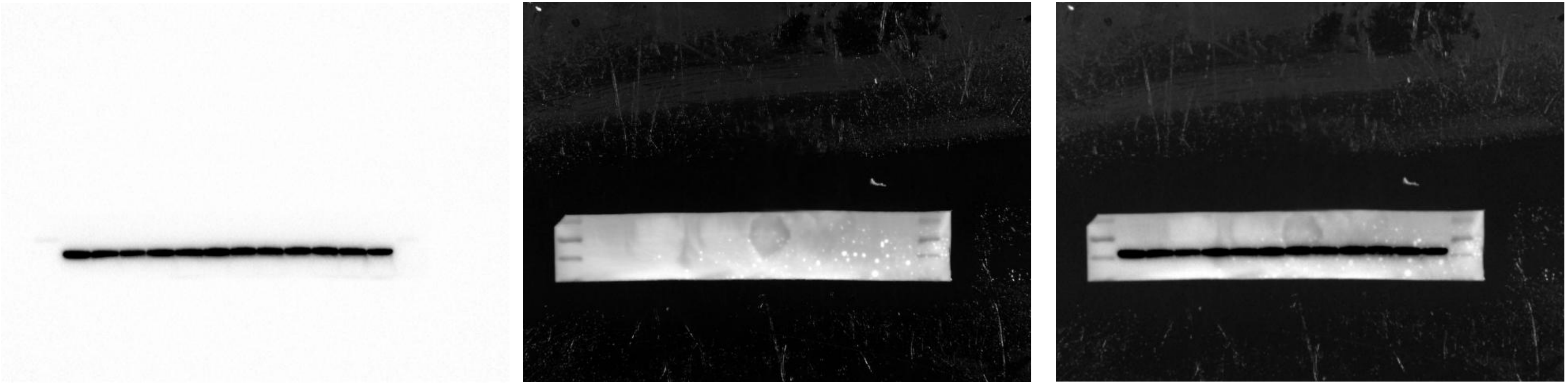

F

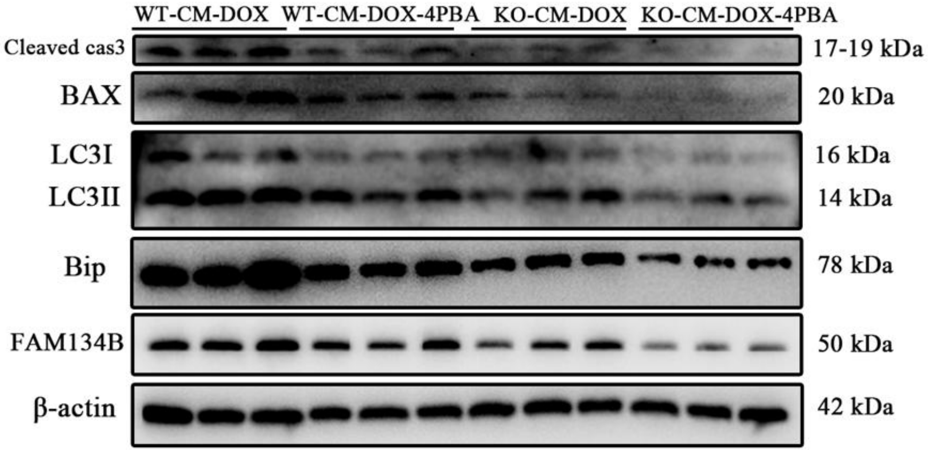
WT-CM-DOX
WT-CM-DOX-4PBA
KO-CM-DOX
KO-CM-DOX-4PBA
Cleaved cas3
17-19 kDa
BAX
20 kDa
LC3I
16 kDa
LC3II
14 kDa
Bip
78 kDa
FAM134B
50 kDa
β-actin
42 kDa

CC3

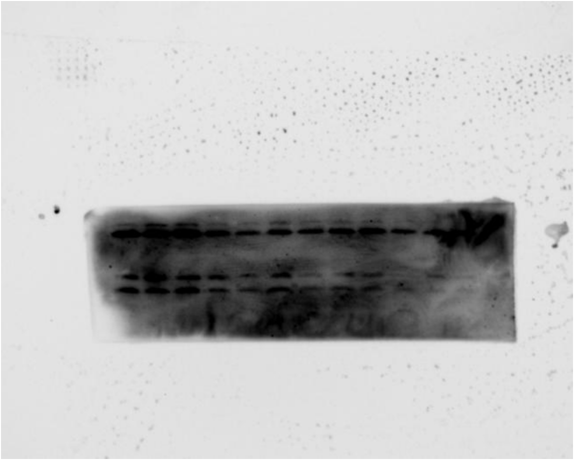

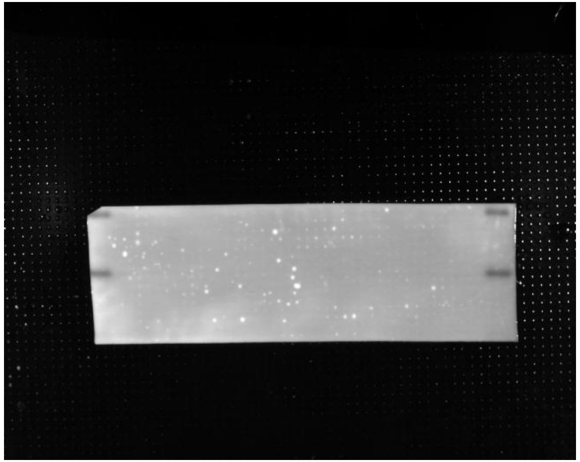

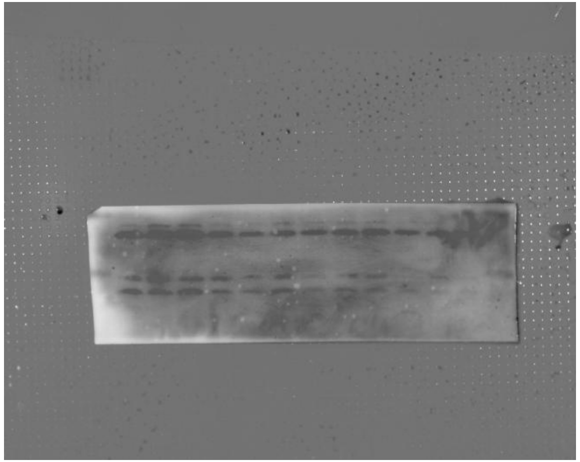

BAX

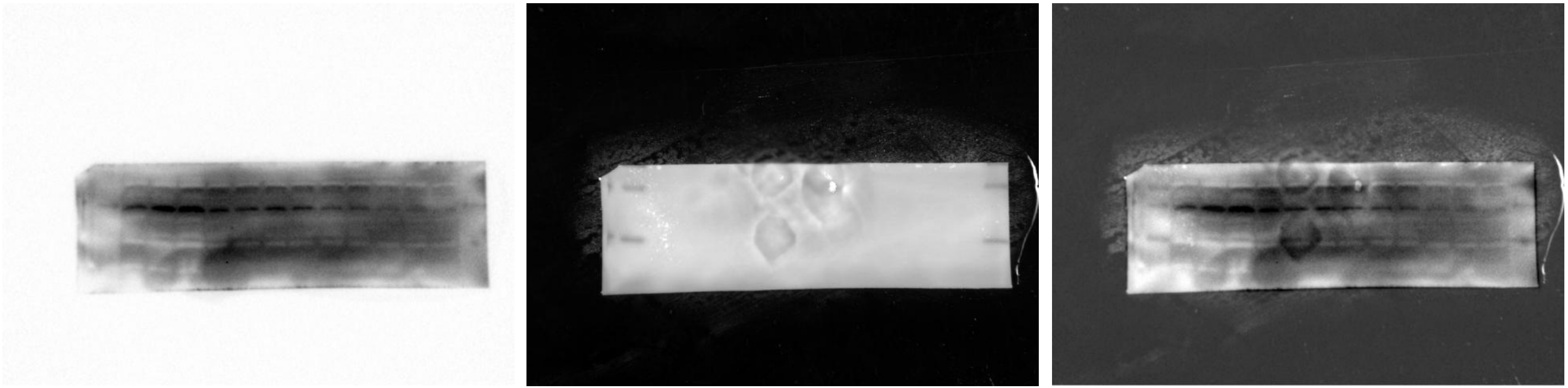

LC3

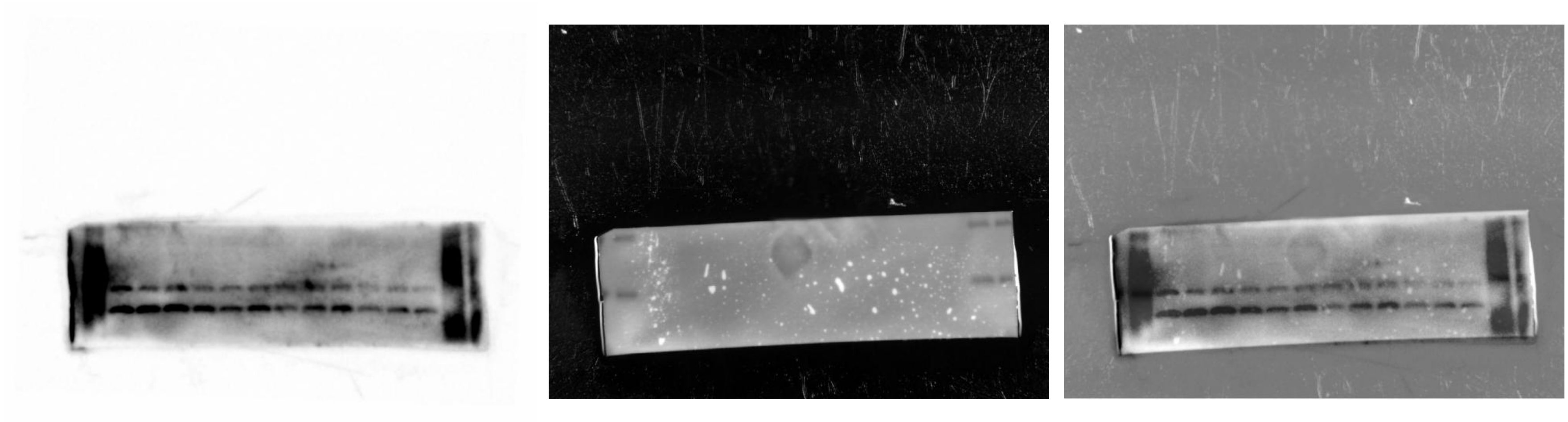

Bip

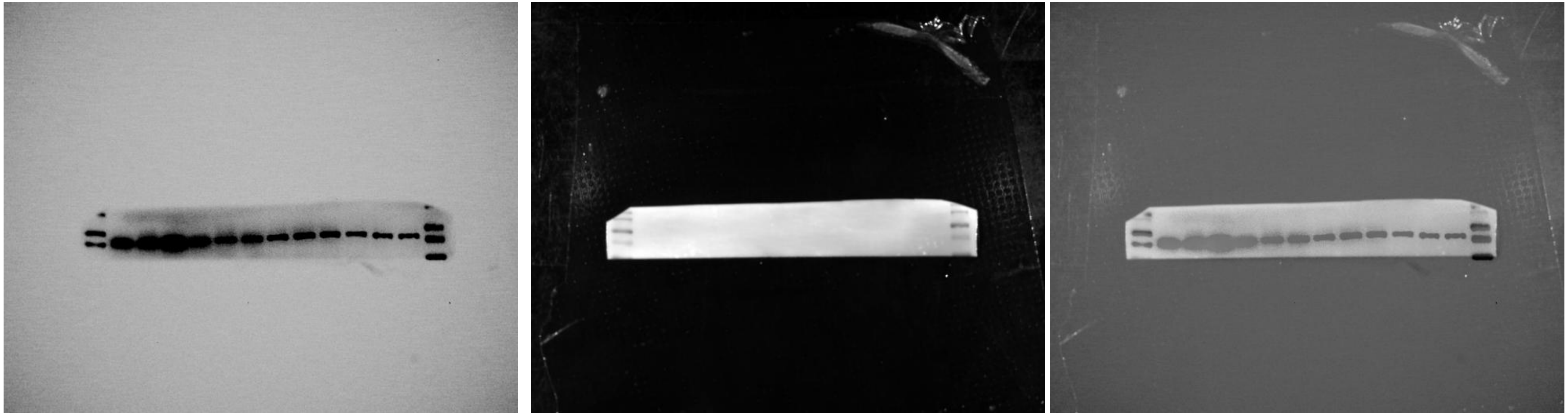

FAM134B

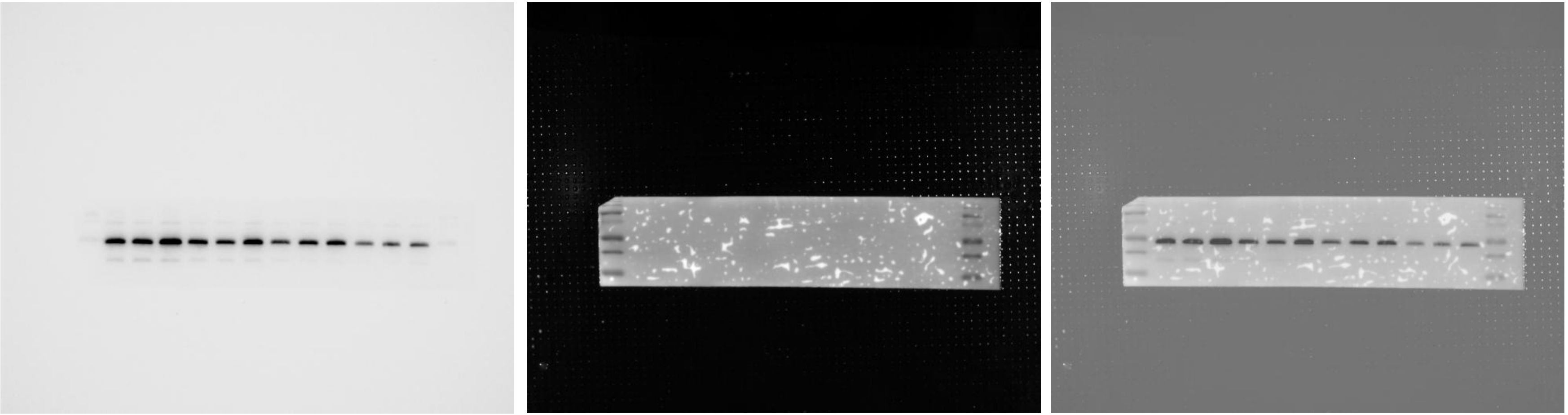

β-actin

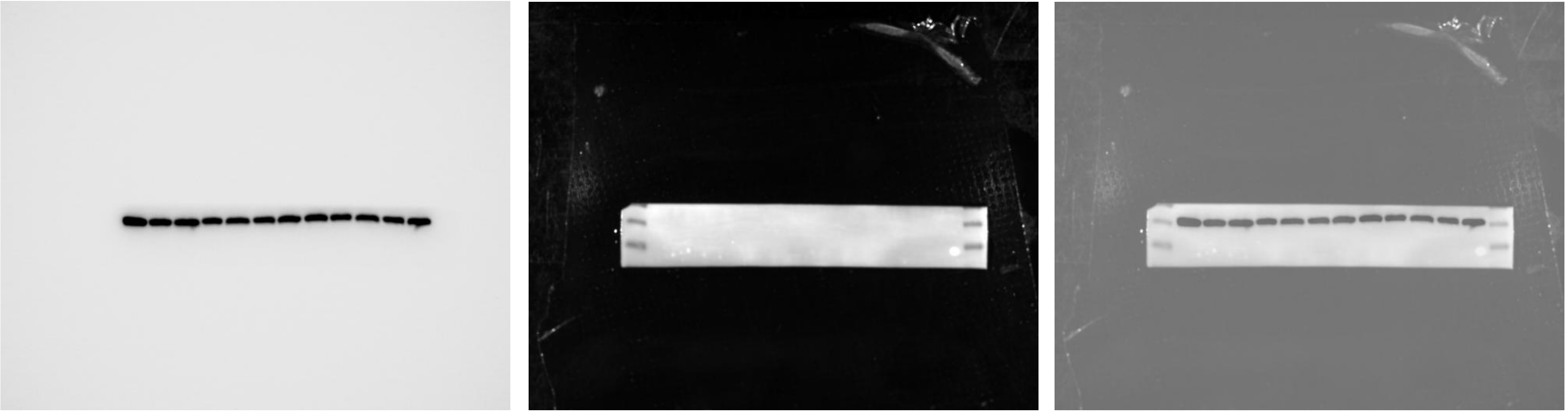

Supplementary Figure 1

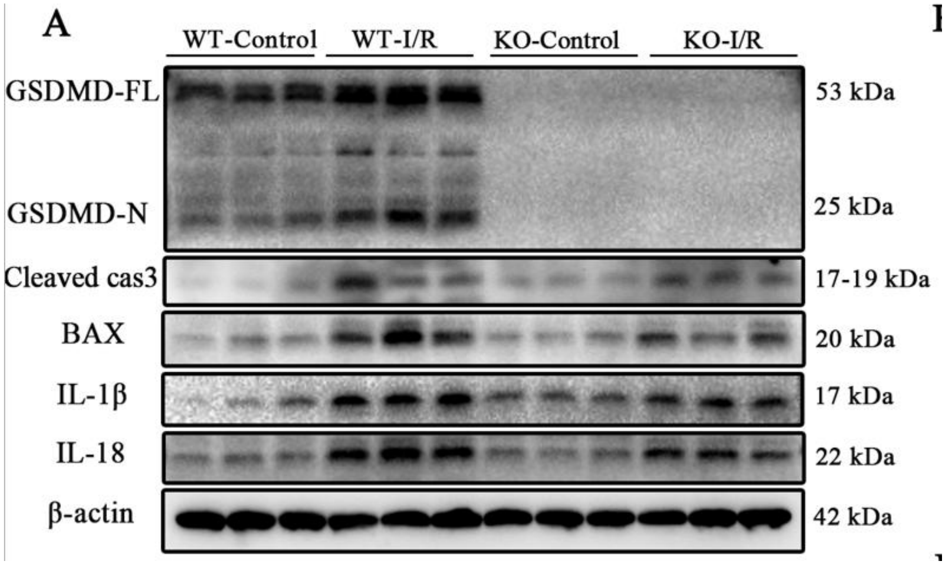
A
WT-Control
WT-I/R
KO-Control
KO-I/R
GSDMD-FL
GSDMD-N
Cleaved cas3
BAX
IL-1β
IL-18
β-actin
53 kDa
25 kDa
17-19 kDa
20 kDa
17 kDa
22 kDa
42 kDa

GSDMD

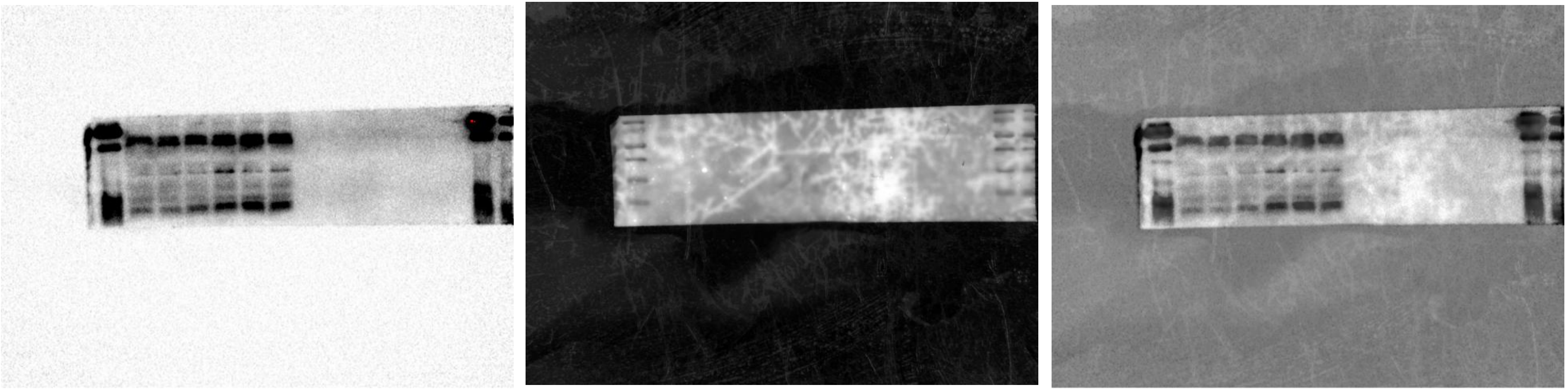

CC3

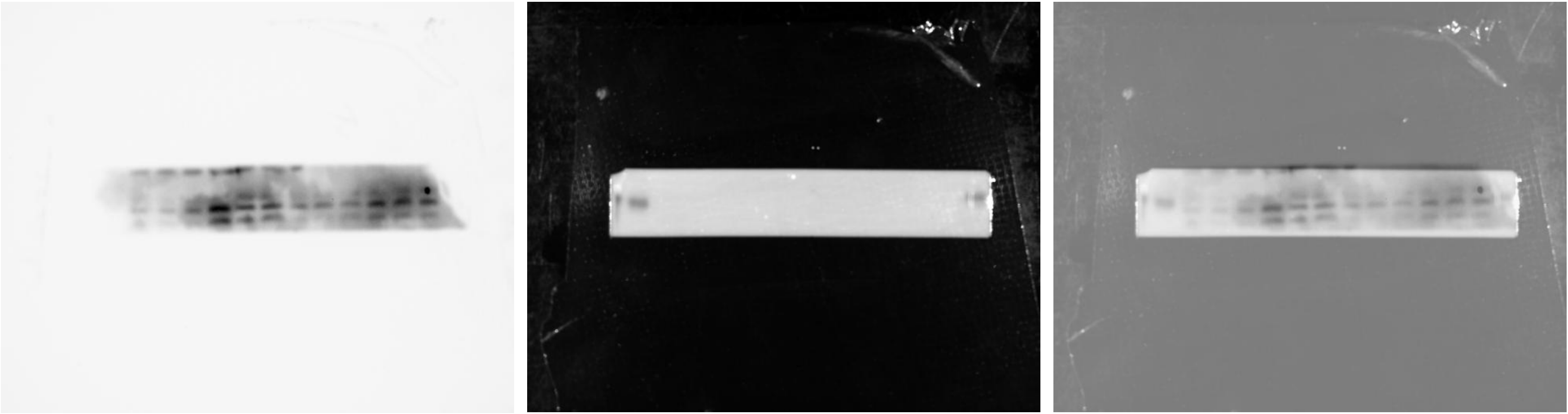

BAX

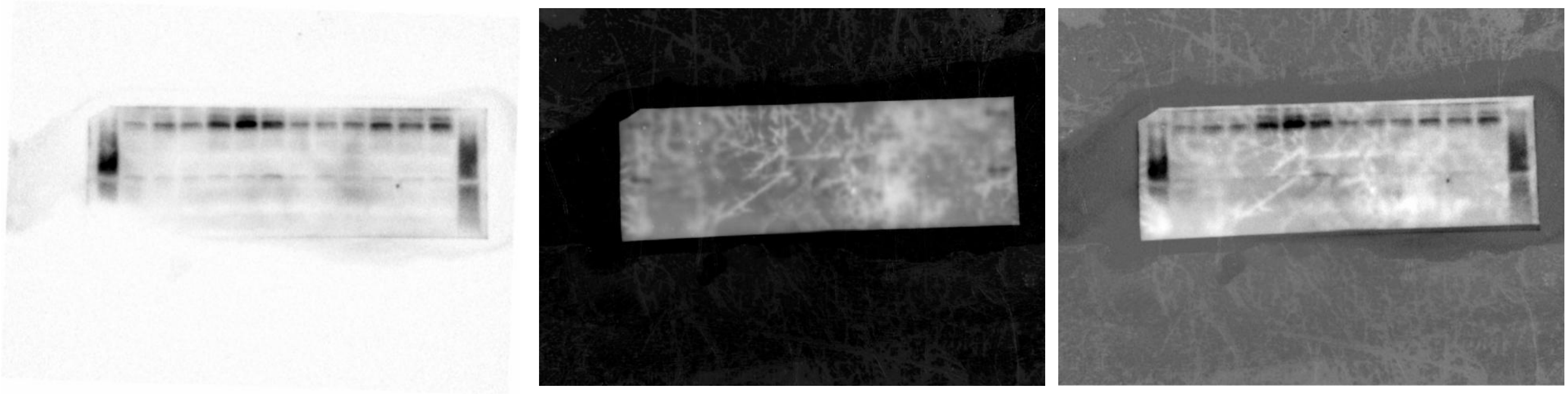

IL-1β

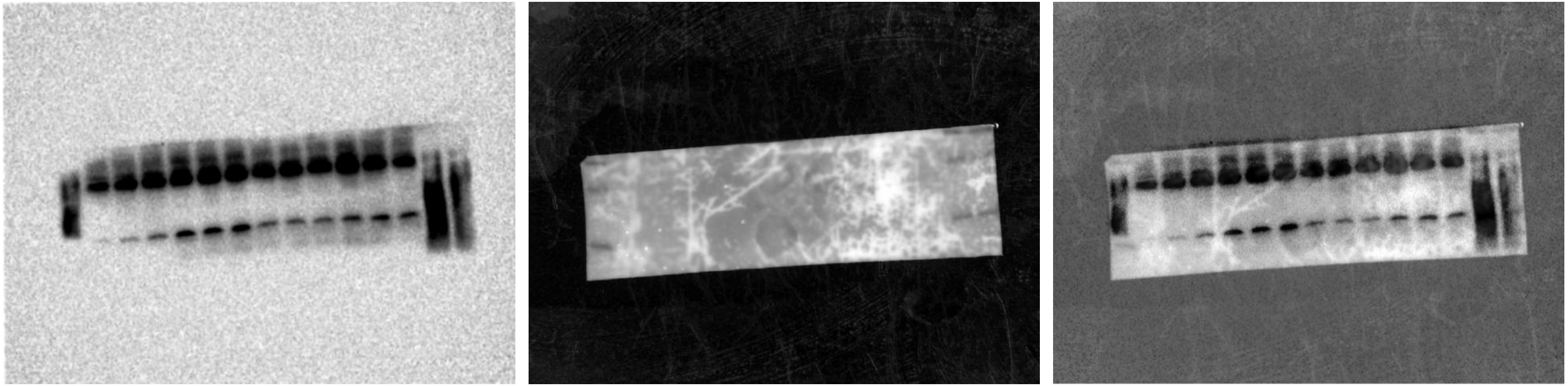

IL-18

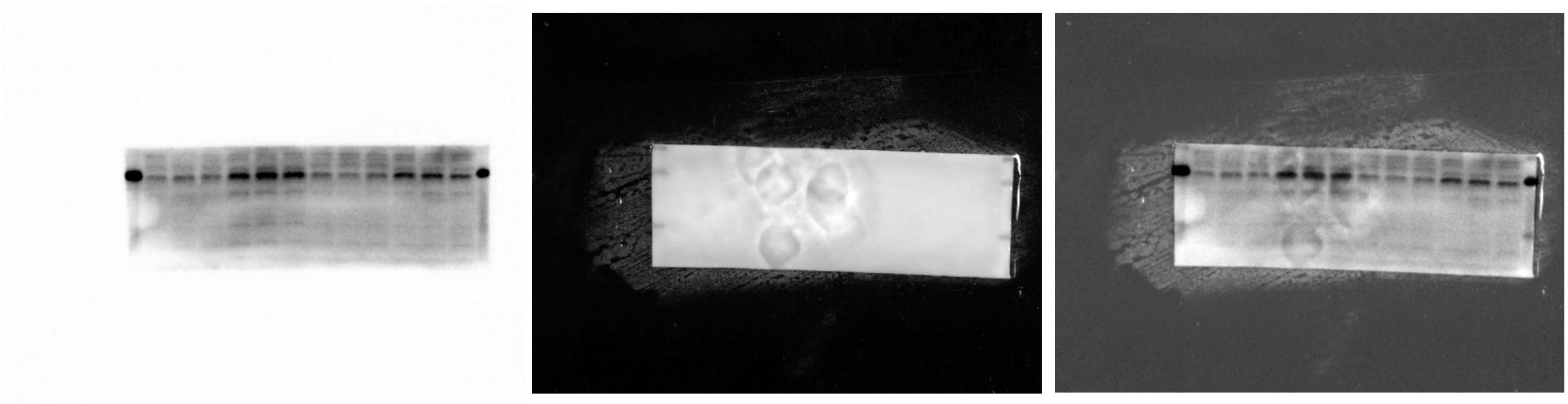

β-actin

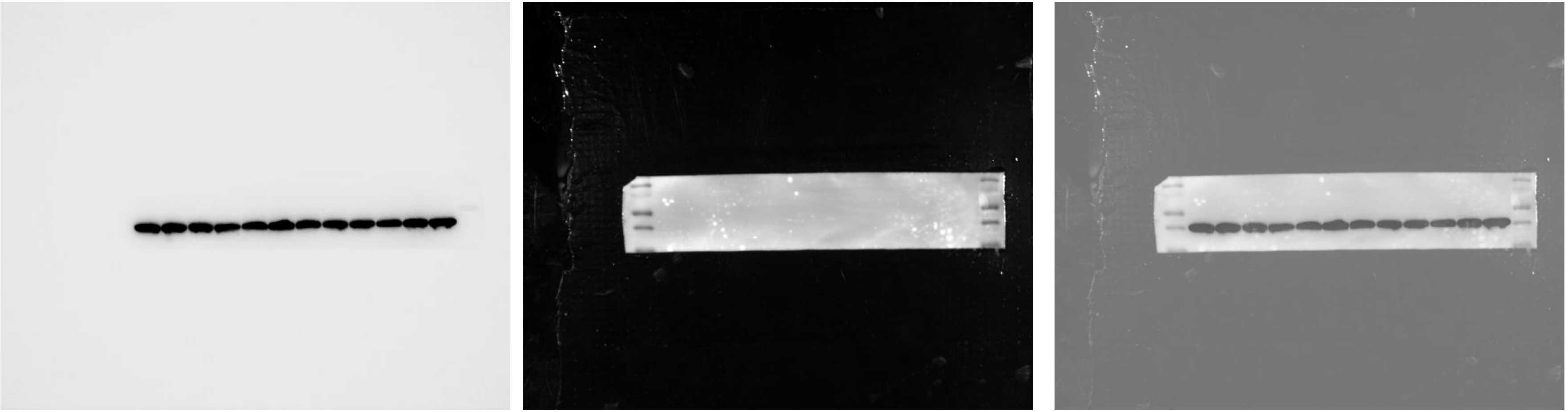

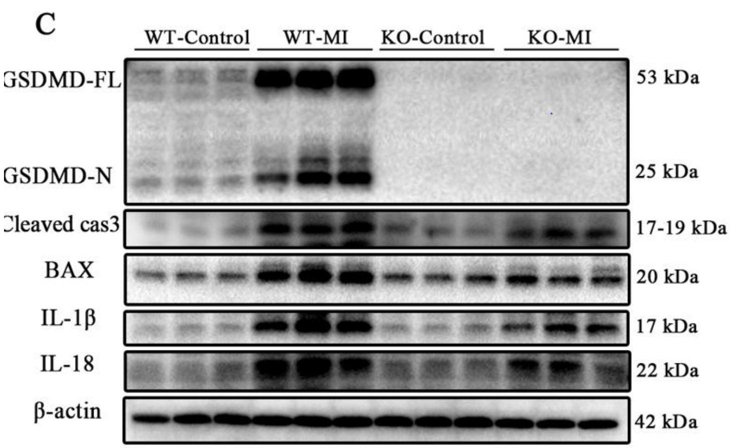
C
WT-Control
WT-MI
KO-Control
KO-MI
GSDMD-FL
GSDMD-N
Cleaved cas3
BAX
IL-1β
IL-18
β-actin
53 kDa
25 kDa
17-19 kDa
20 kDa
17 kDa
22 kDa
42 kDa

GSDMD

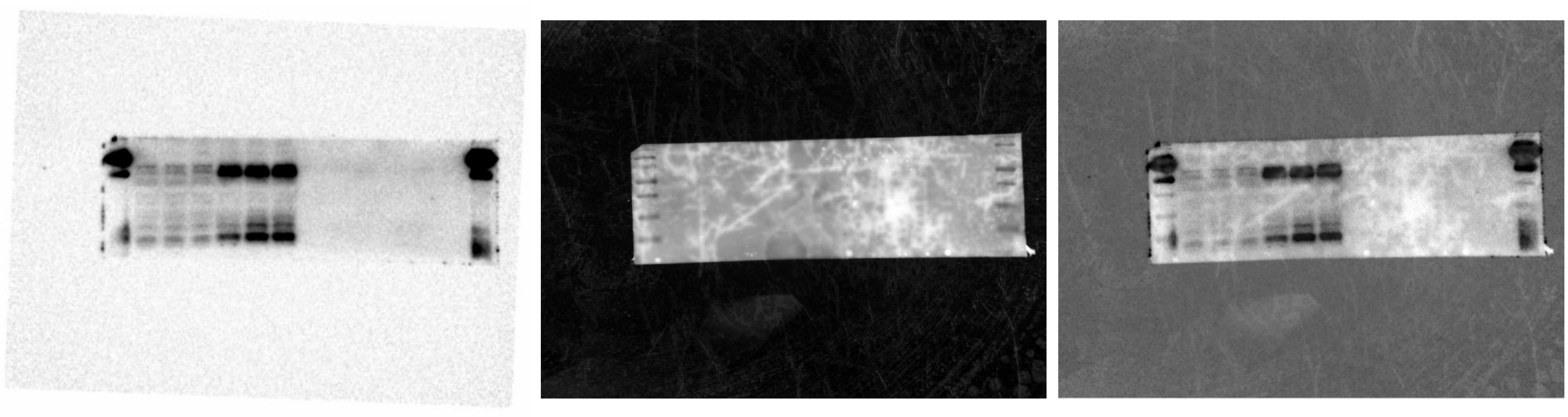

CC3

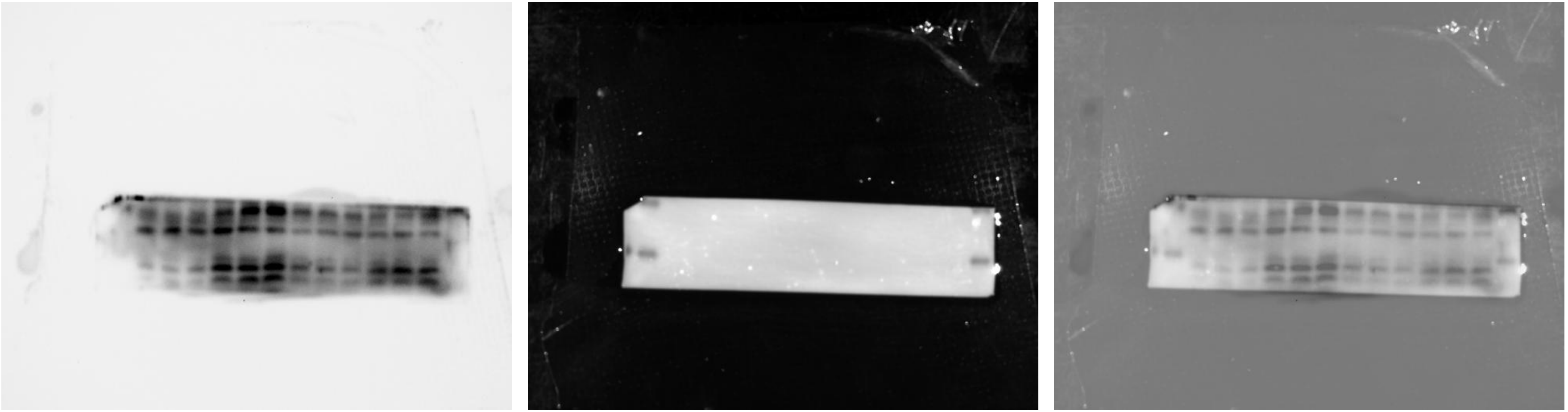

BAX

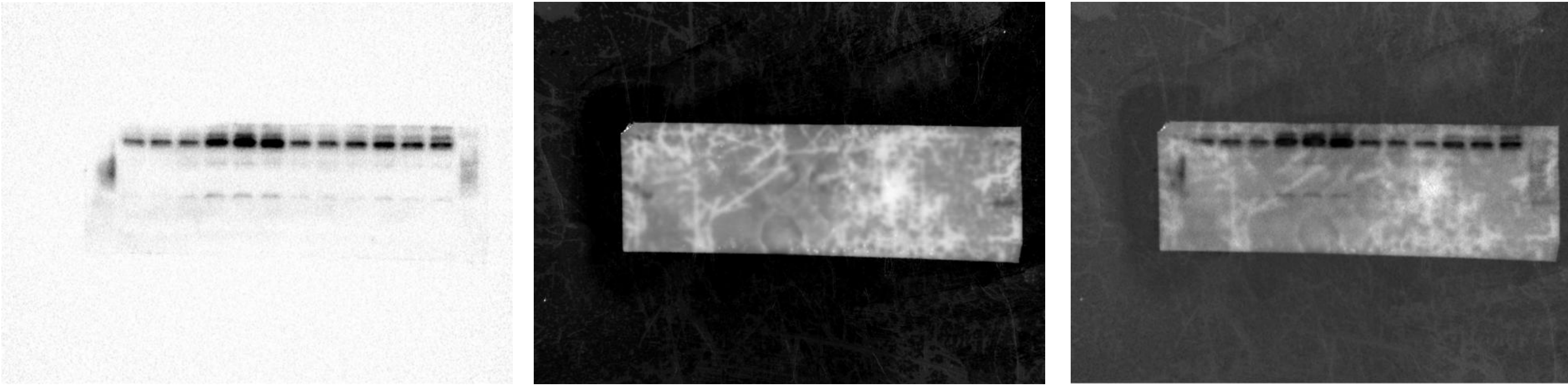

IL-1β

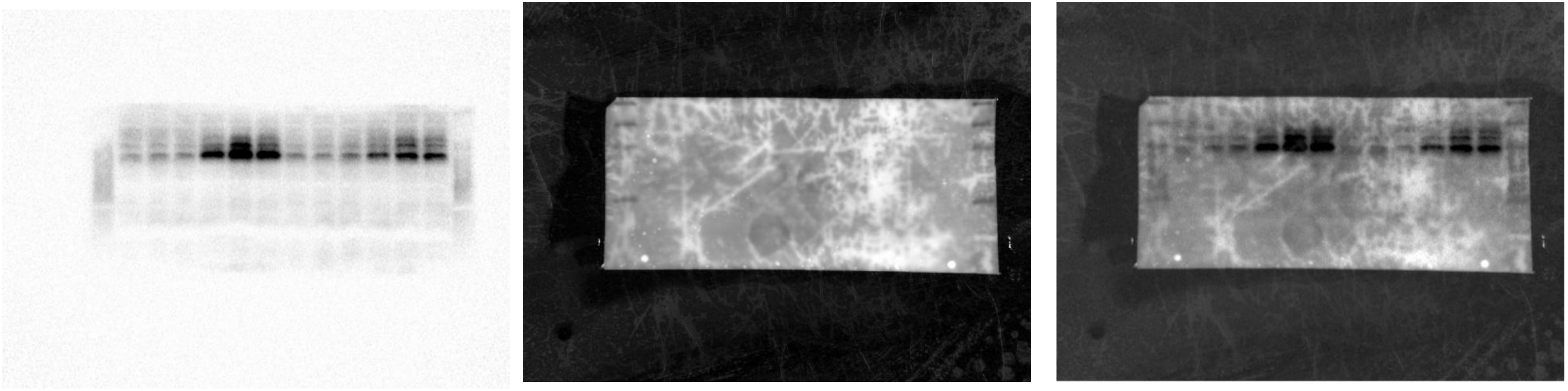

IL-18

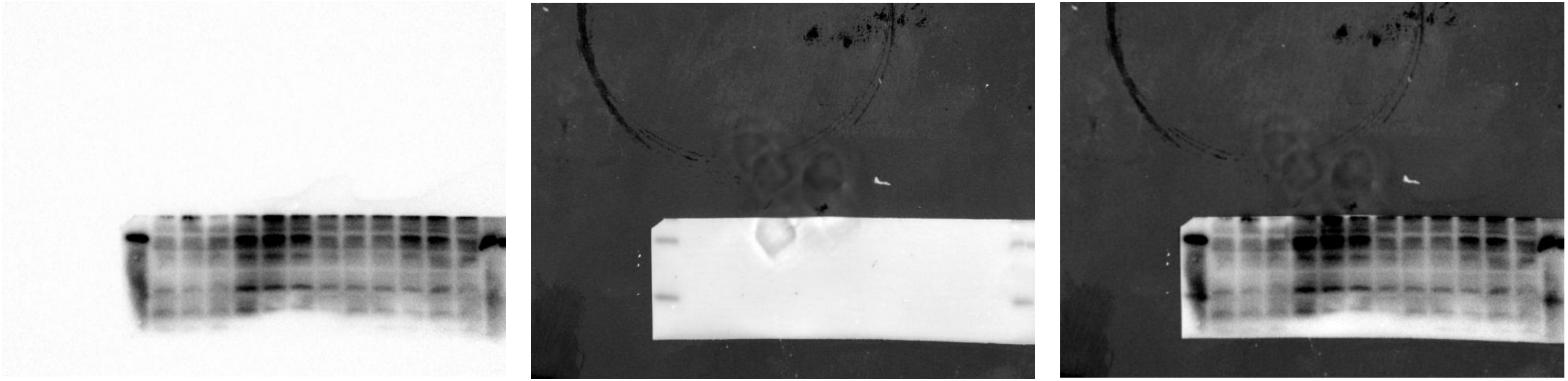

β-actin

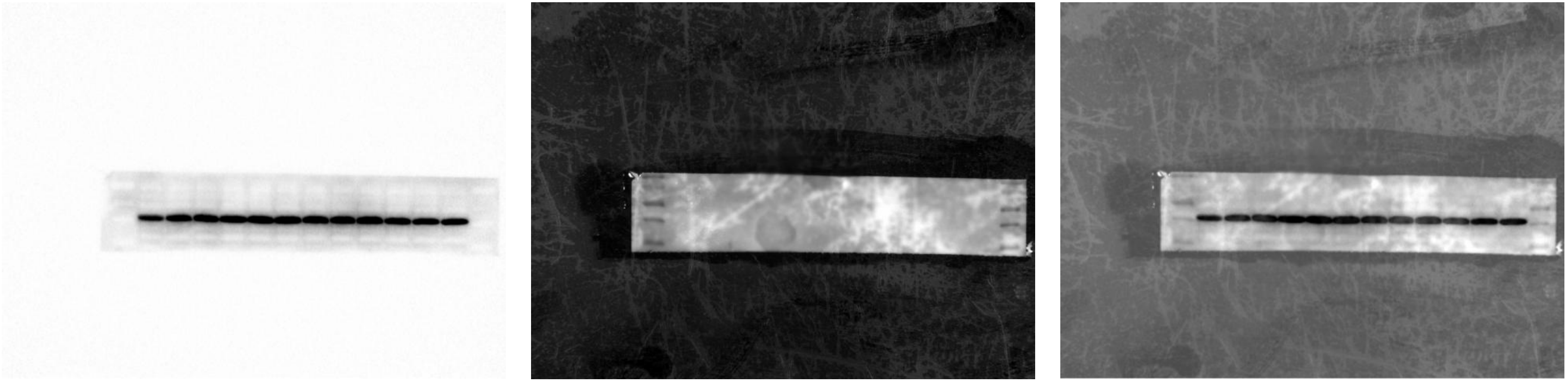

Supplementary Figure 2

H

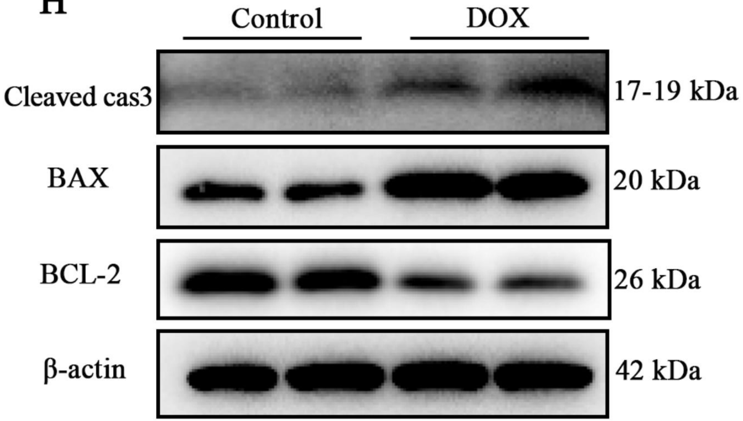
Control
DOX
Cleaved cas3
17-19 kDa
BAX
20 kDa
BCL-2
26 kDa
β-actin
42 kDa

CC3

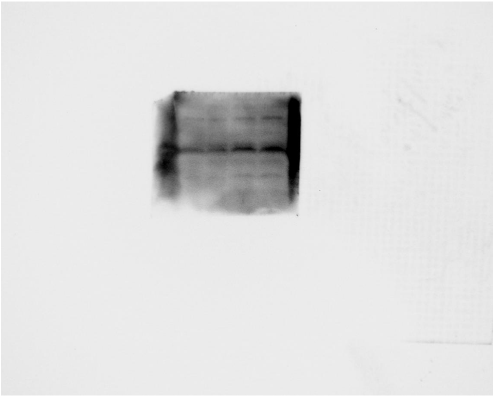

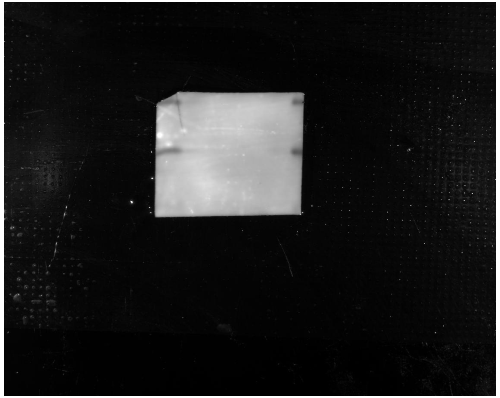

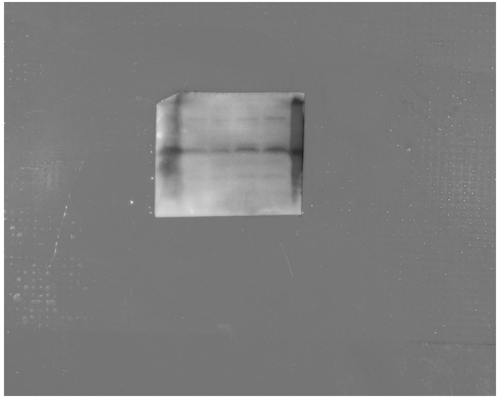

BAX

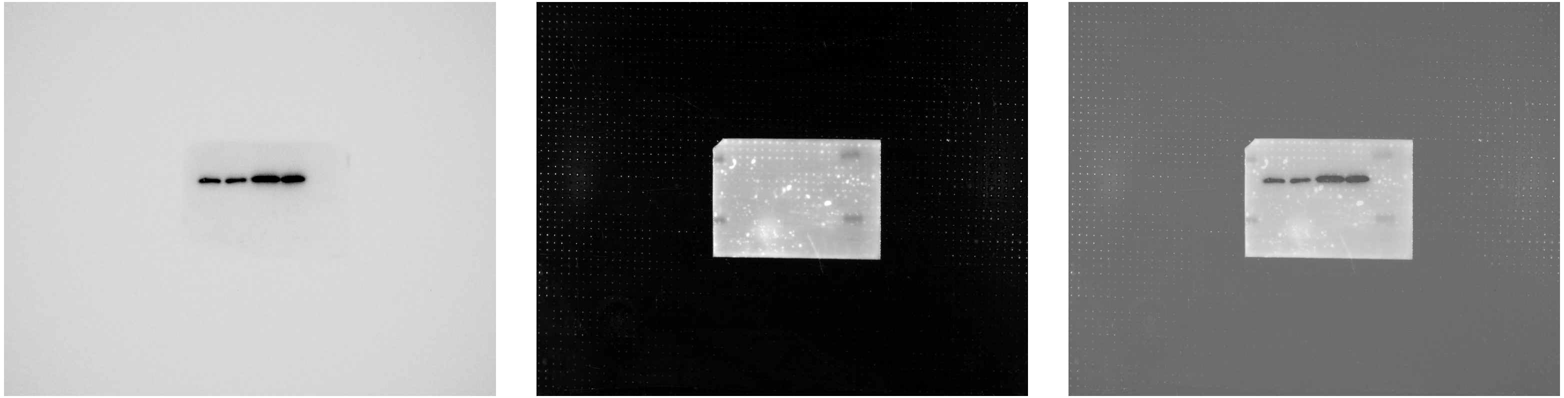

BCL-2

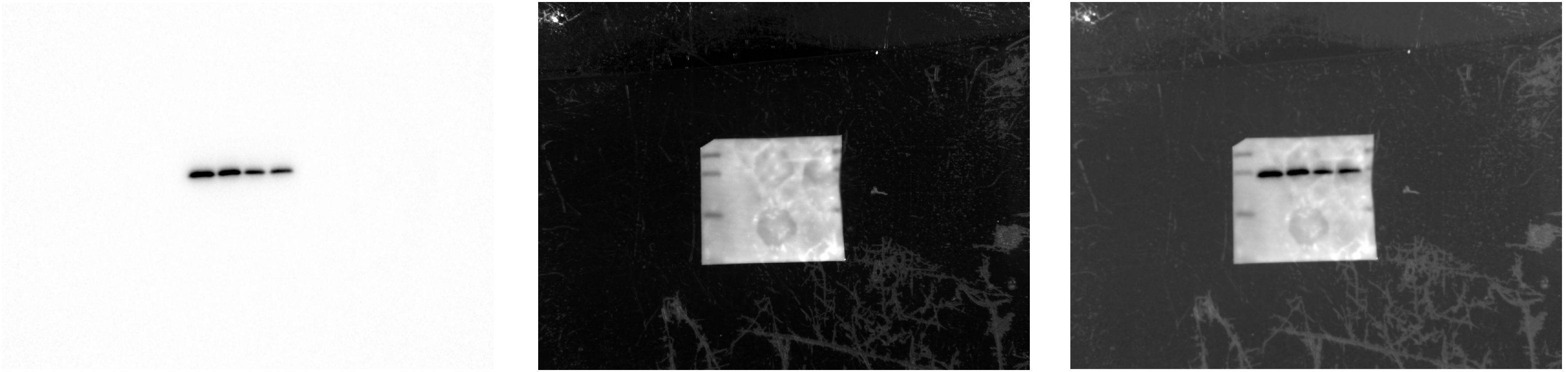

β-actin

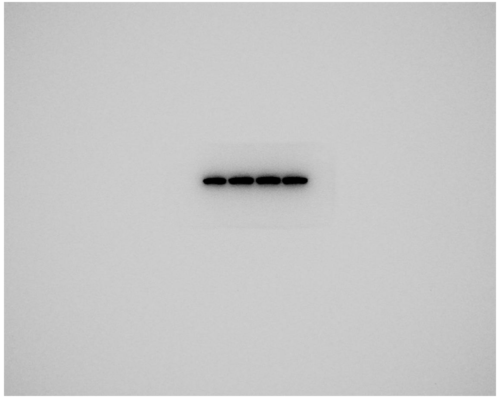

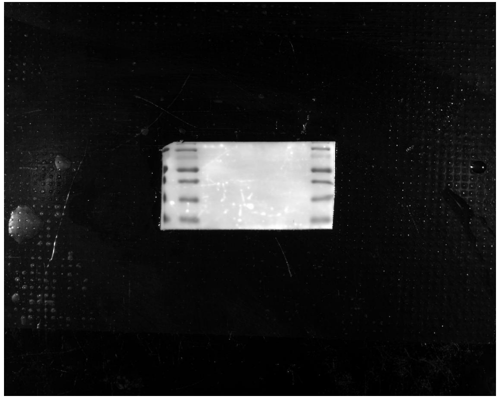

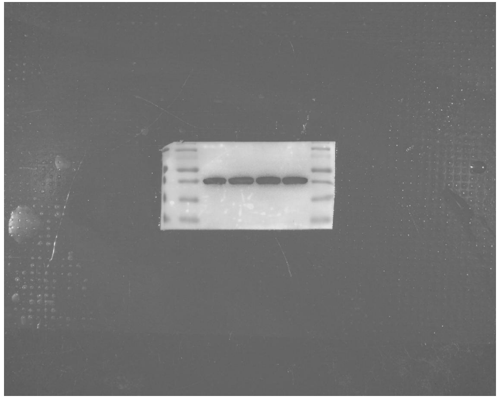

K

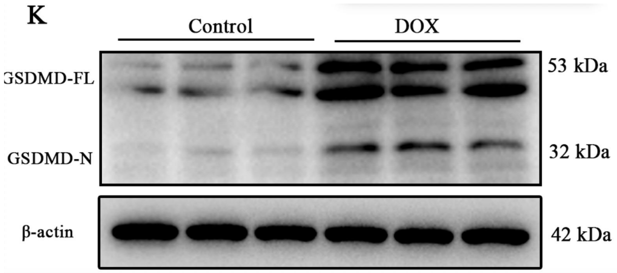

GSDMD

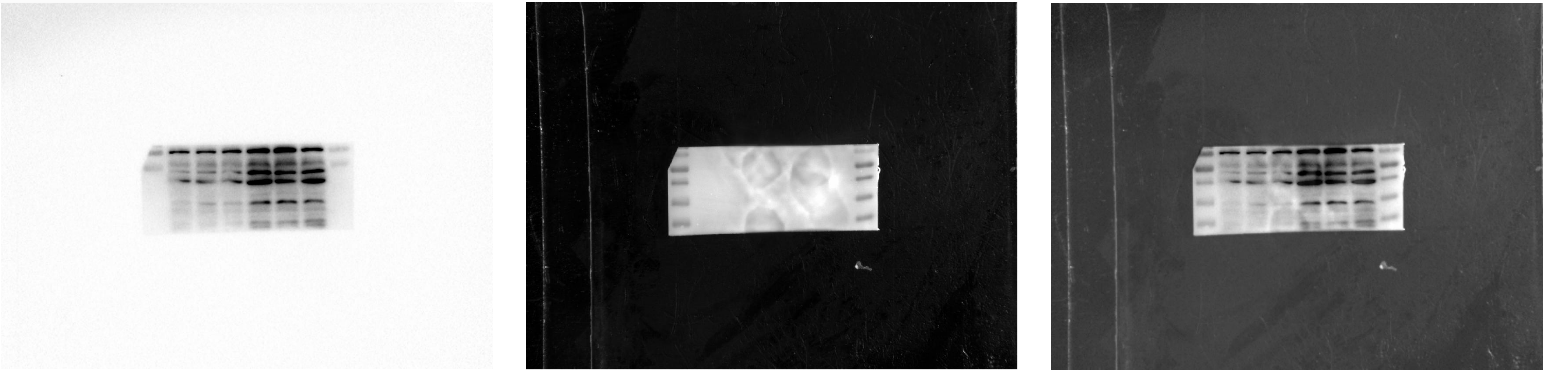

β-actin

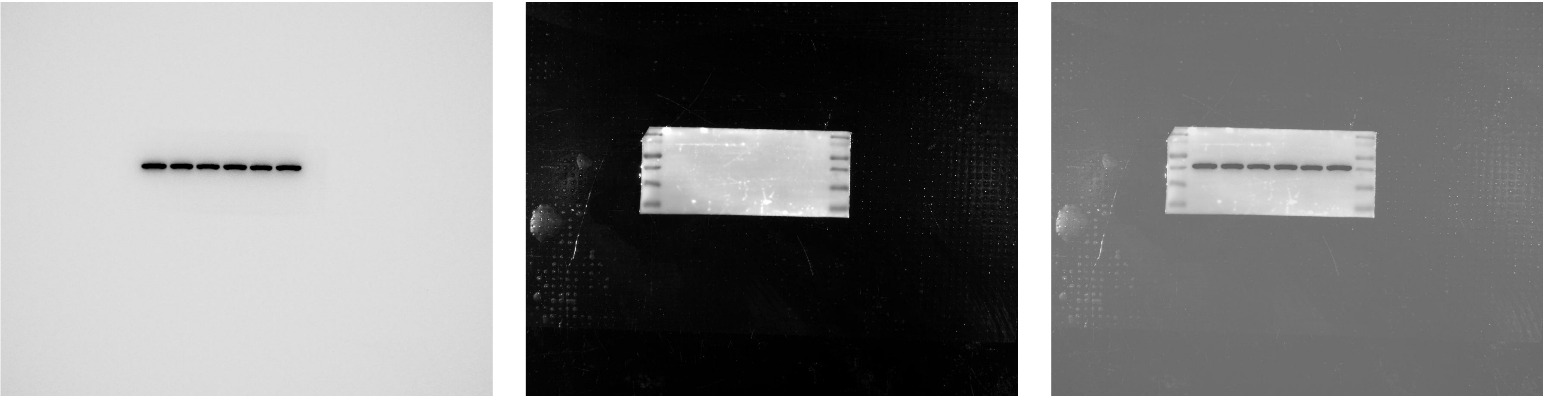

Supplementary Figure 3

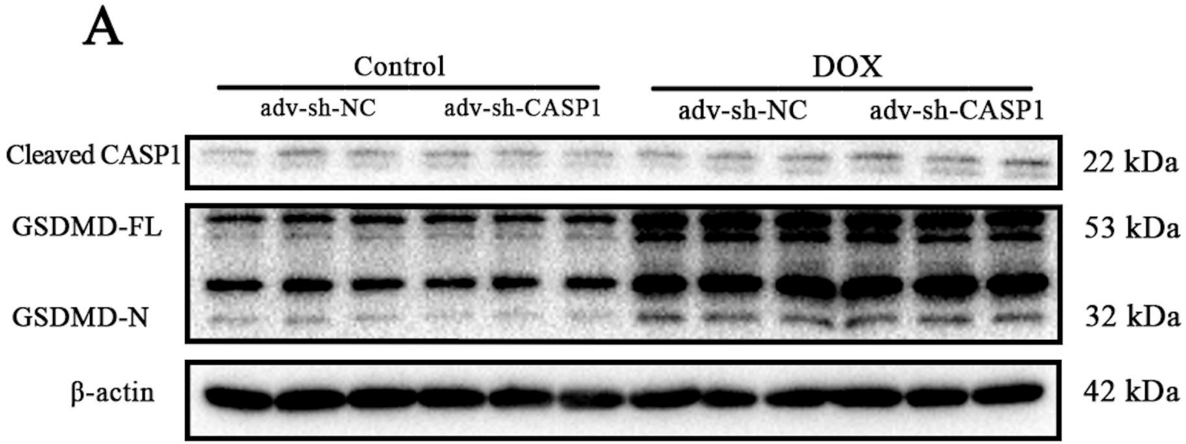

Cleaved CASP1

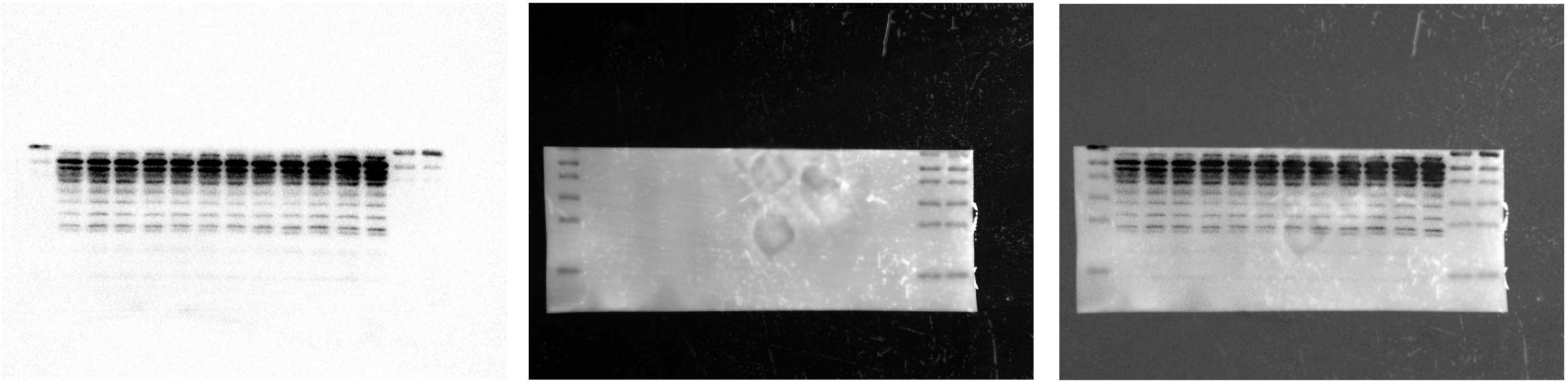

GSDMD

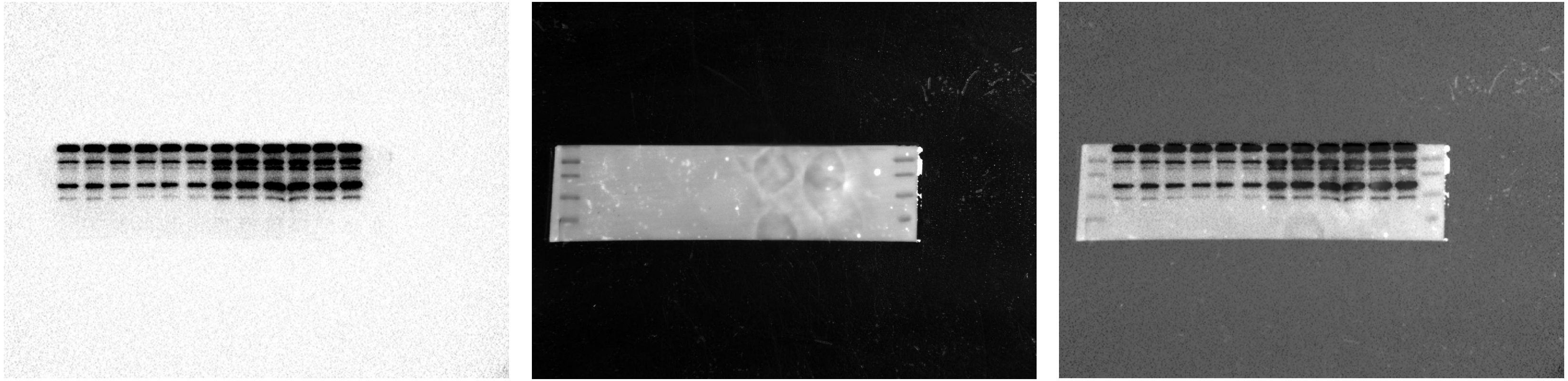

β-actin

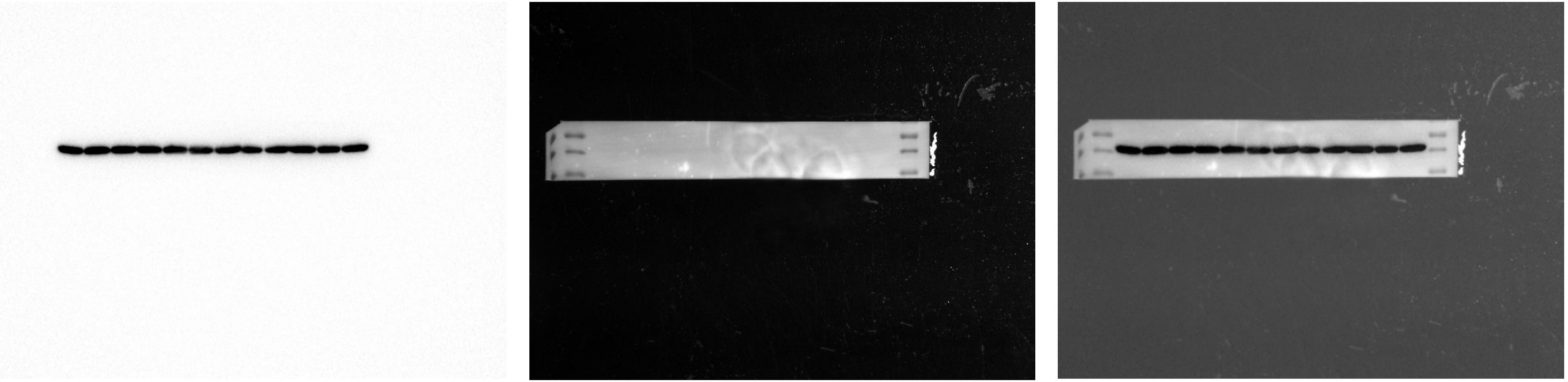

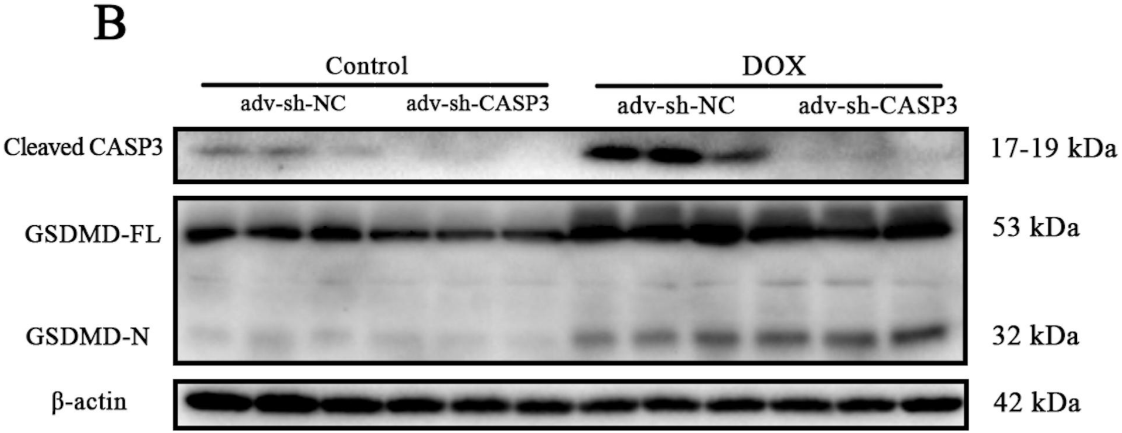

Cleaved CASP3

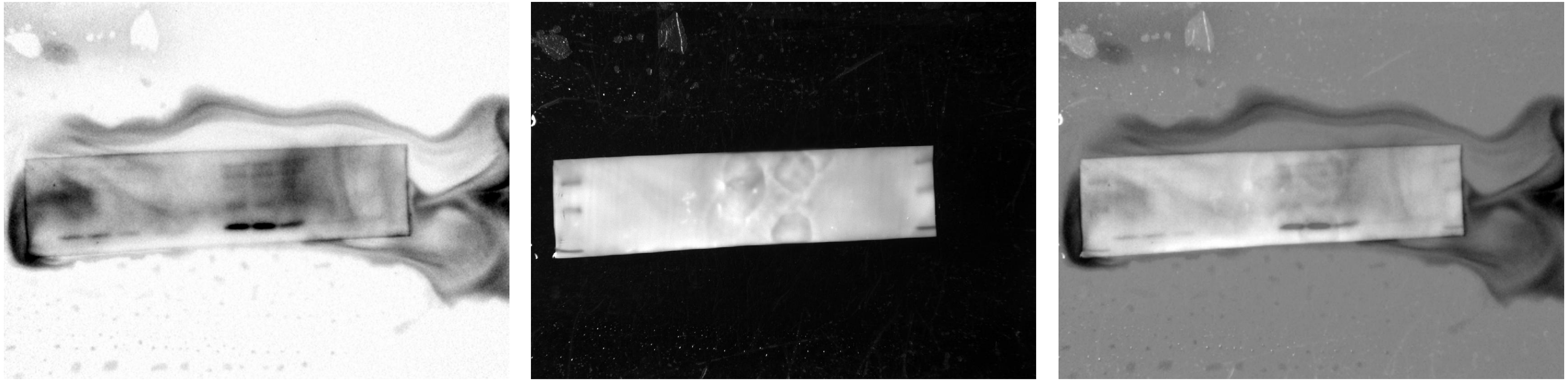

GSDMD

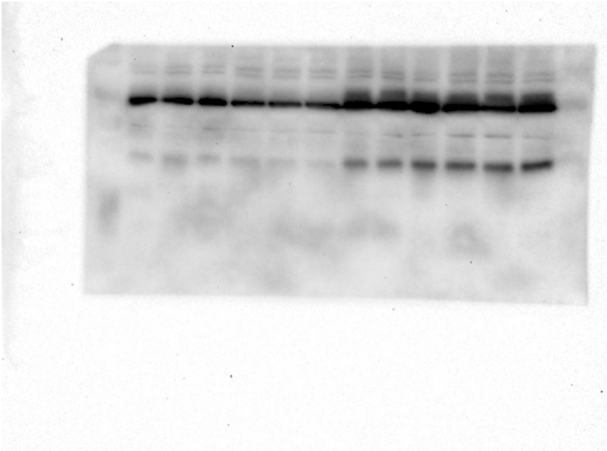

β-actin

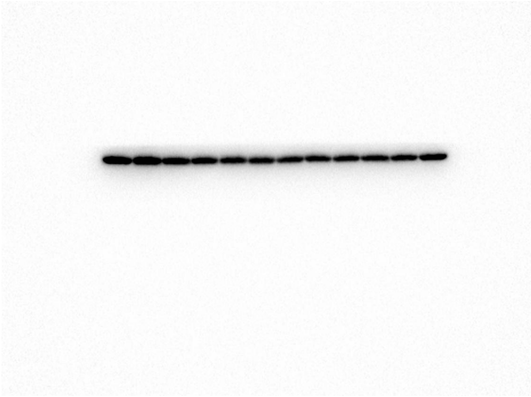

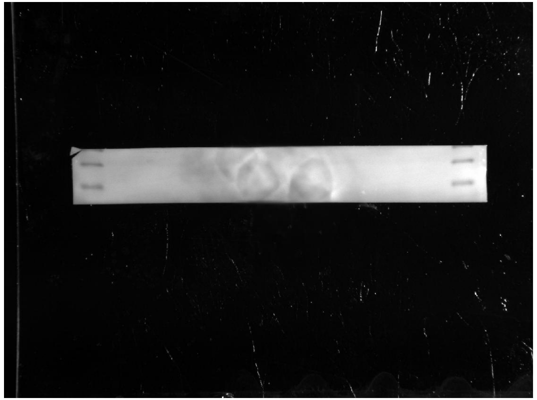

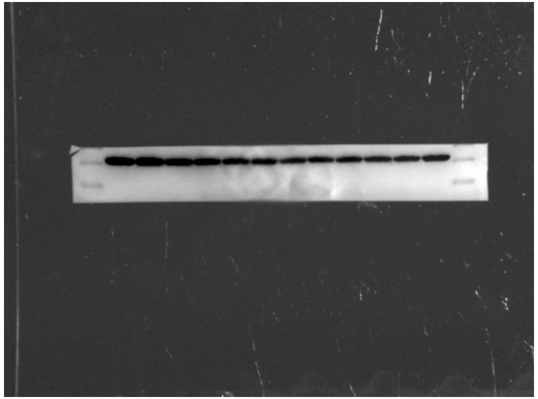

C

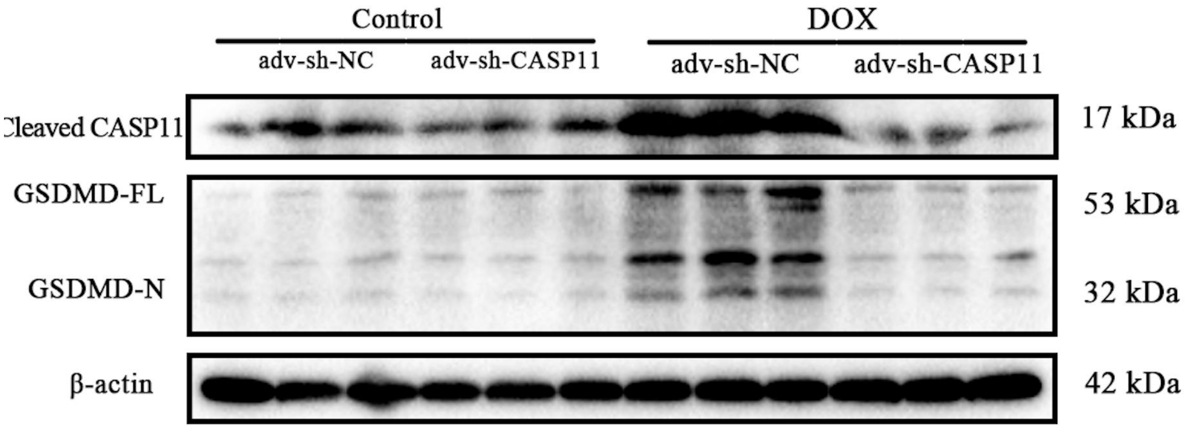

Cleaved CASP11

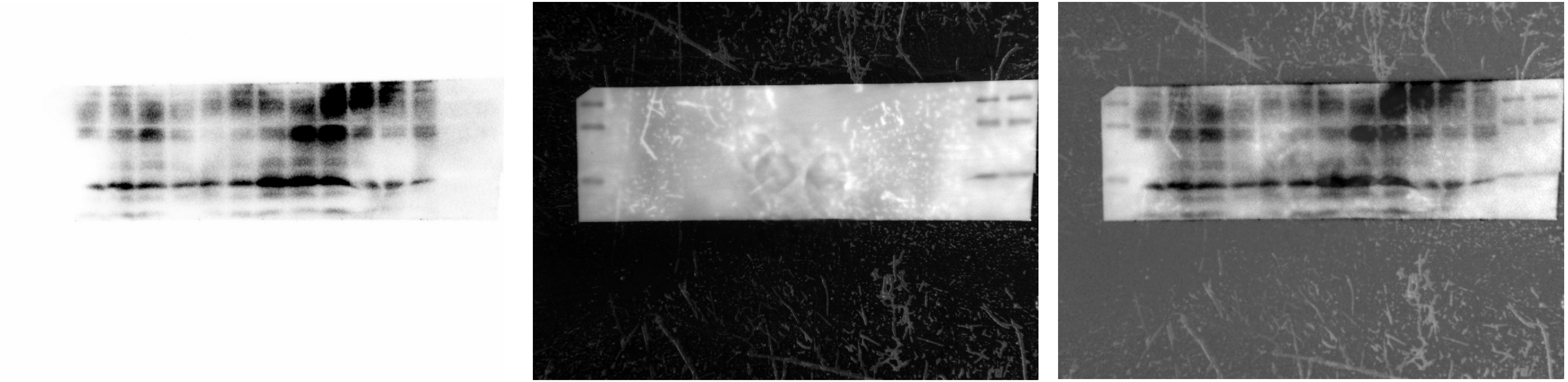

GSDMD

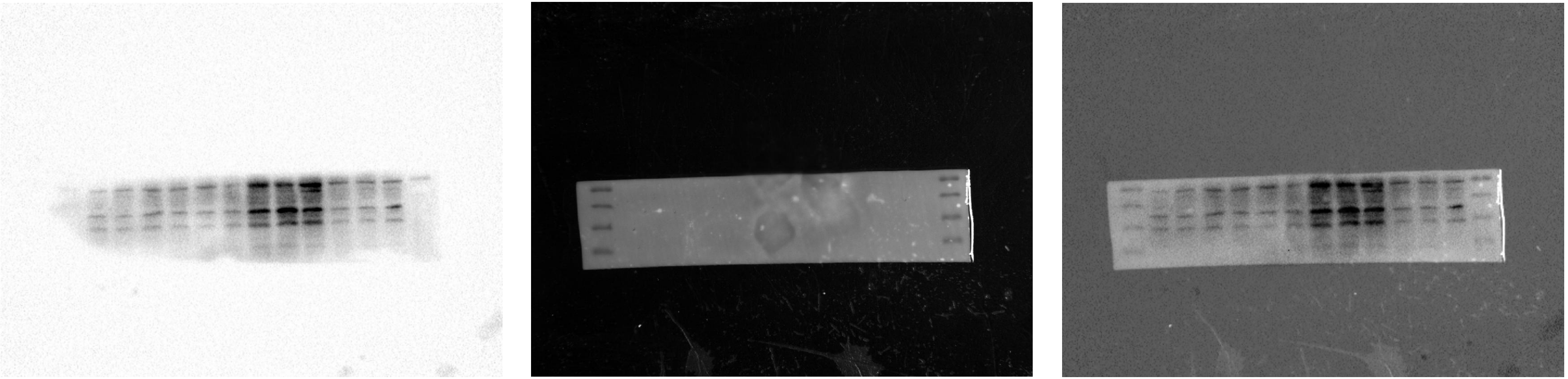

β-actin

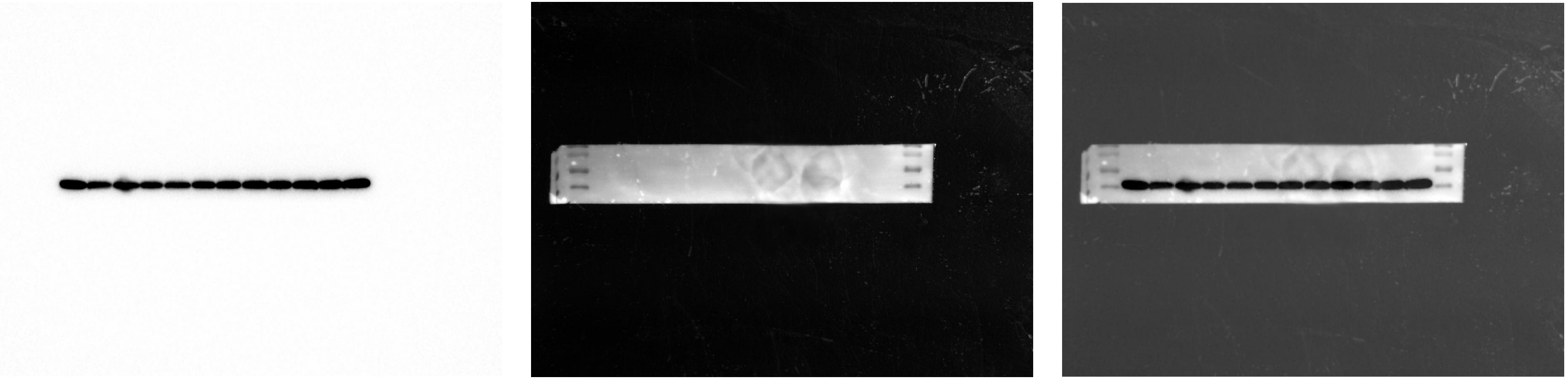

Supplementary Figure 4

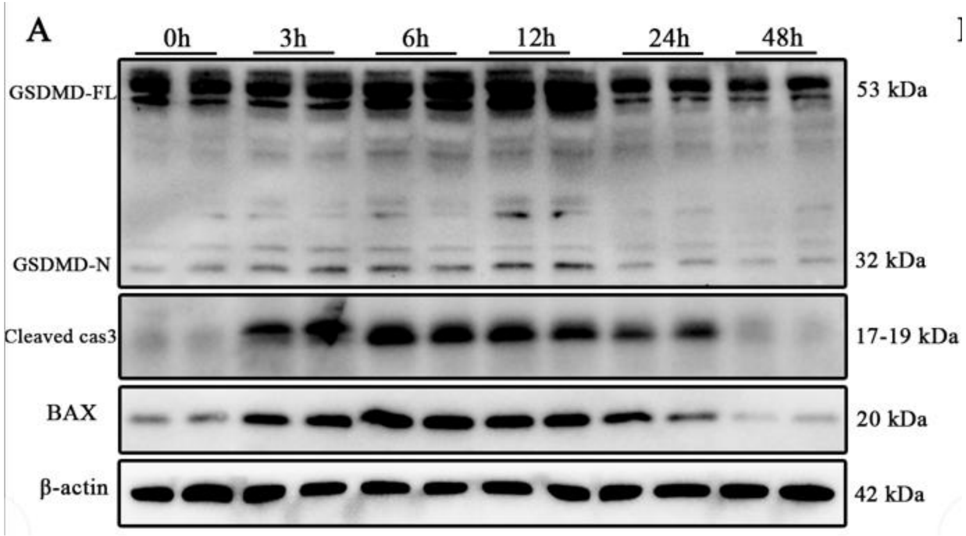
A
0h
3h
6h
12h
24h
48h
GSDMD-FL
GSDMD-N
Cleaved cas3
BAX
β-actin
53 kDa
32 kDa
17-19 kDa
20 kDa
42 kDa

GSDMD

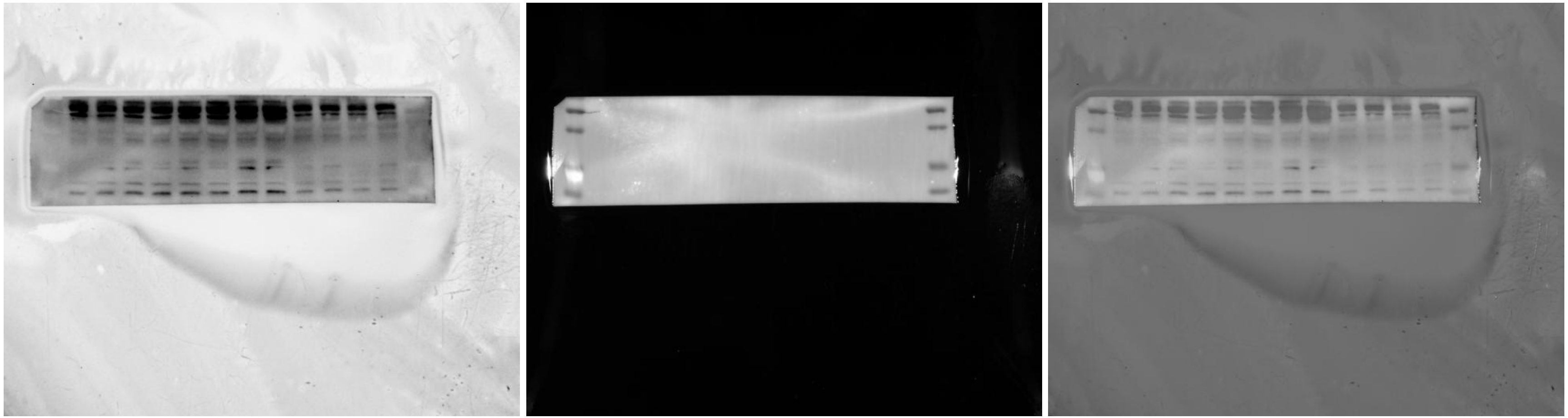

BAX

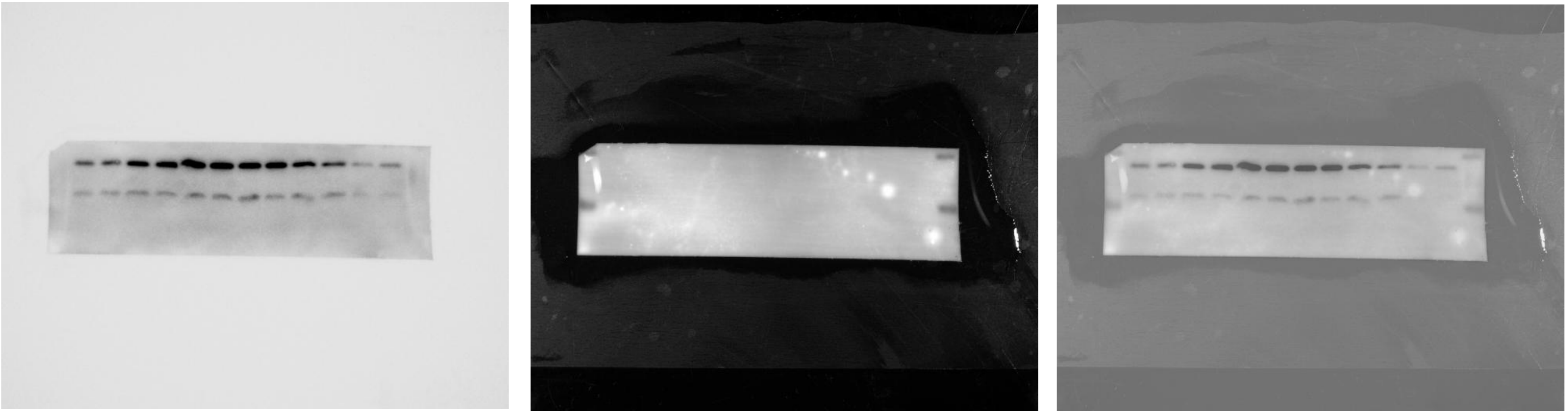

CC3

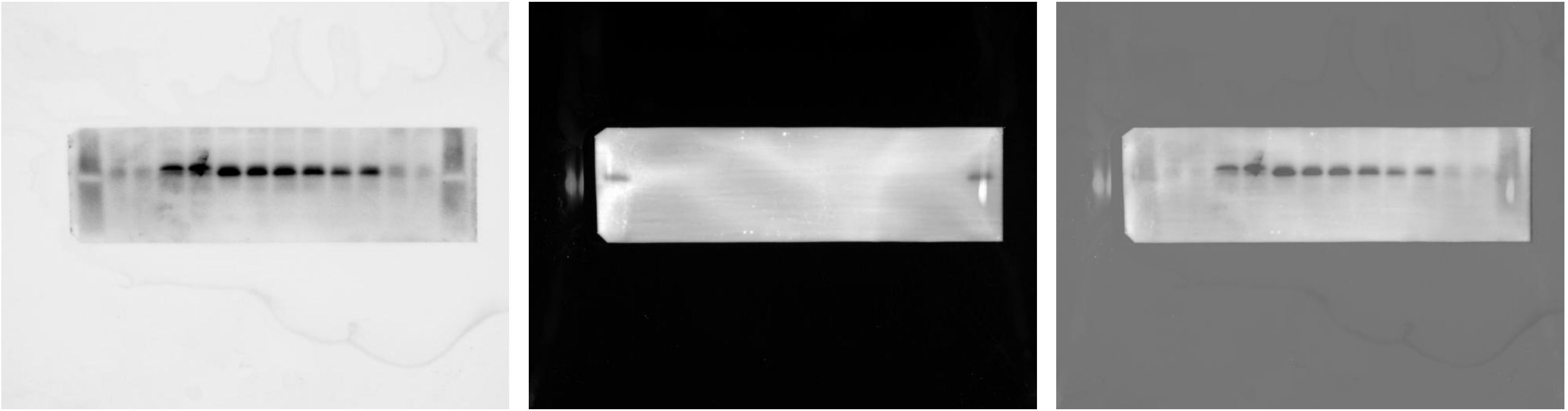

β-actin

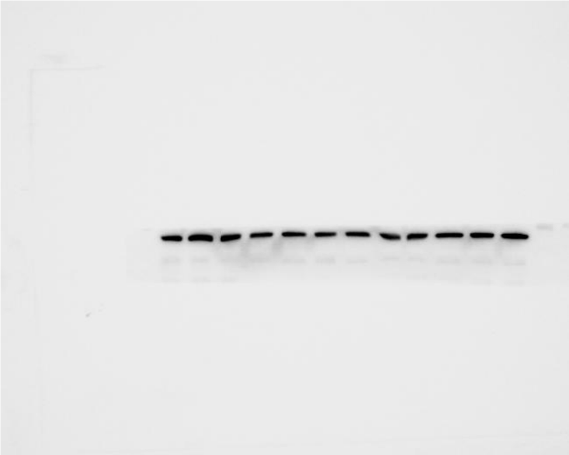

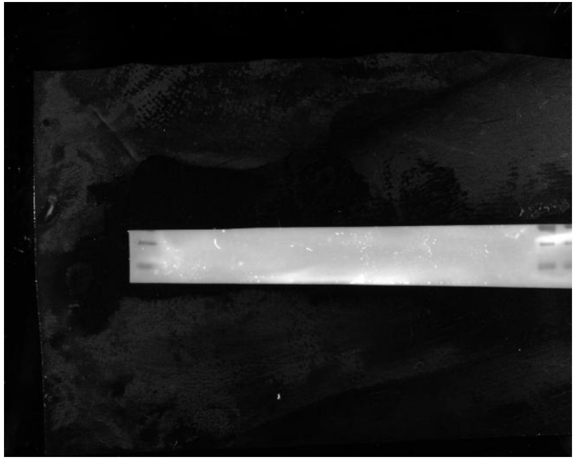

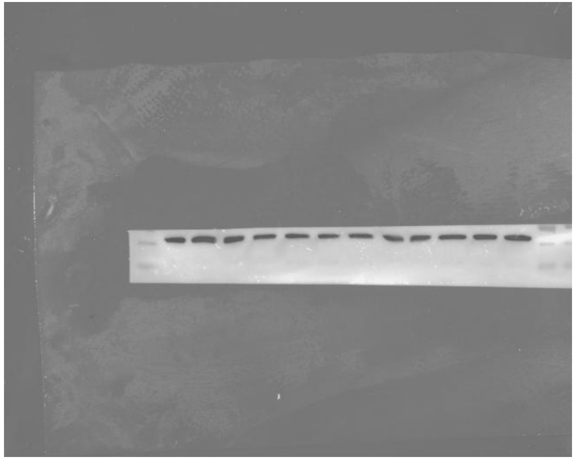

Supplementary Figure 9

H

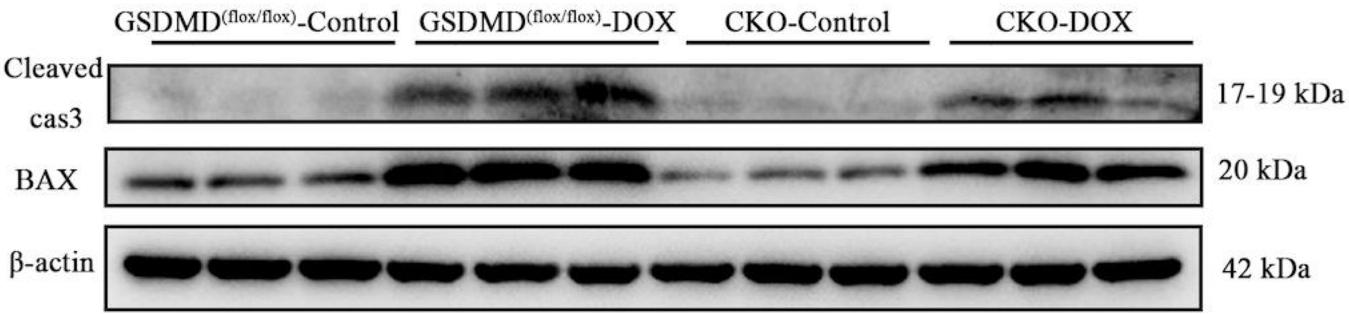
GSDMD(flox/flox)-Control
GSDMD(flox/flox)-DOX
CKO-Control
CKO-DOX
Cleaved cas3
BAX
β-actin
17-19 kDa
20 kDa
42 kDa

CC3

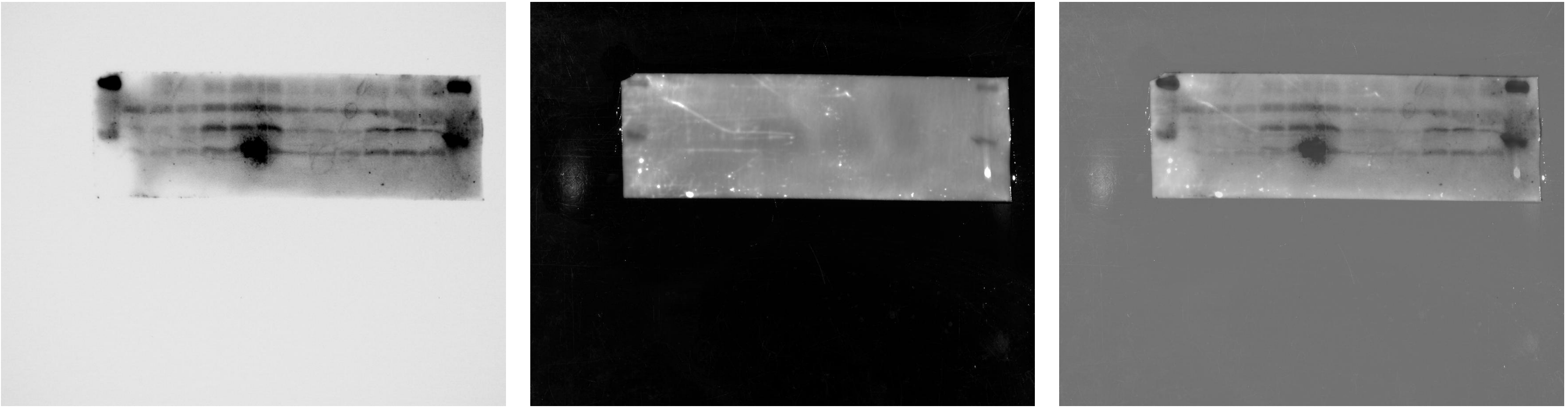

BAX

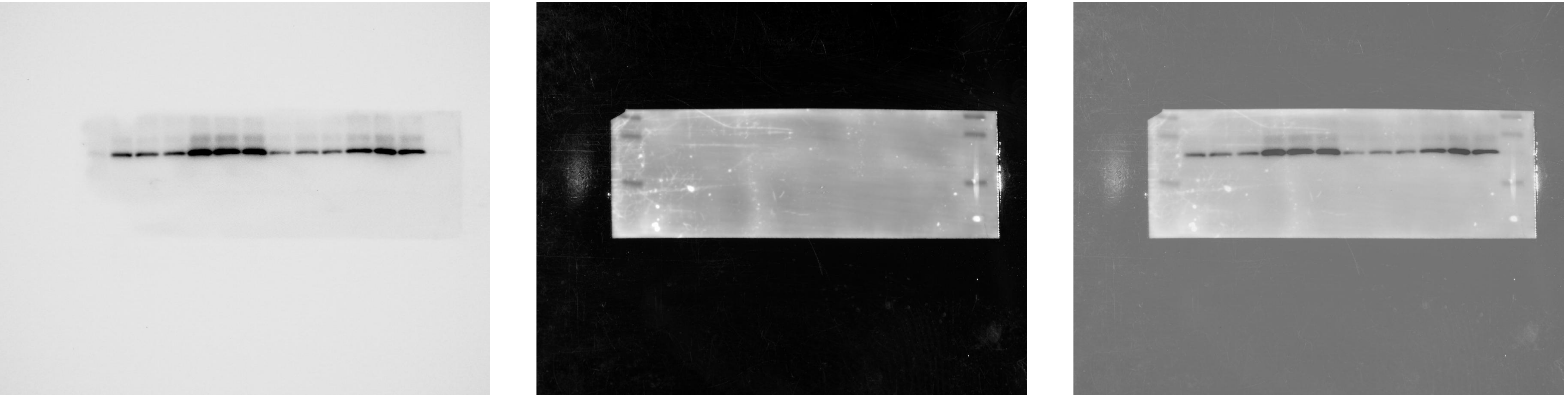

β-actin

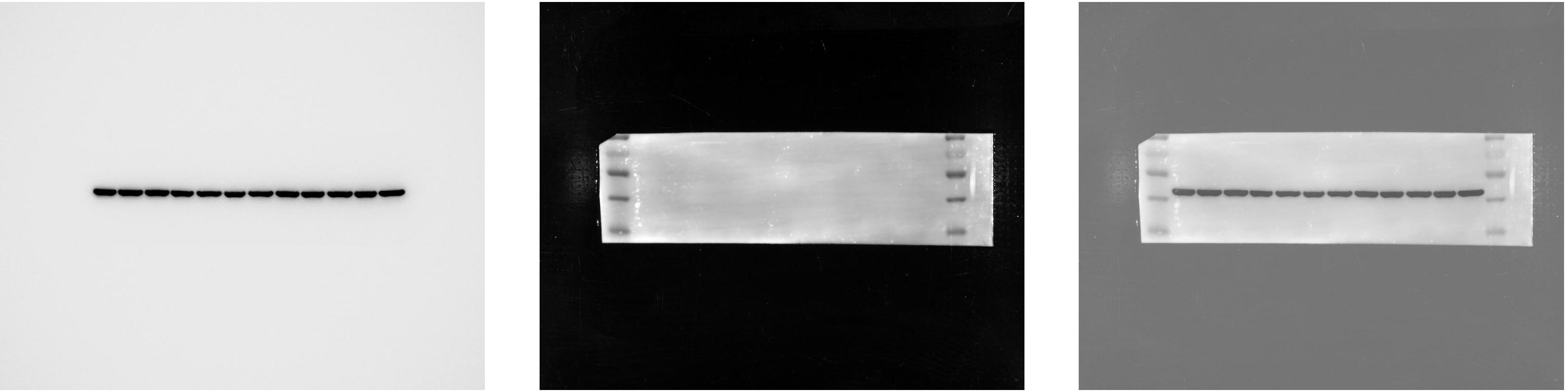

Supplementary Figure 10

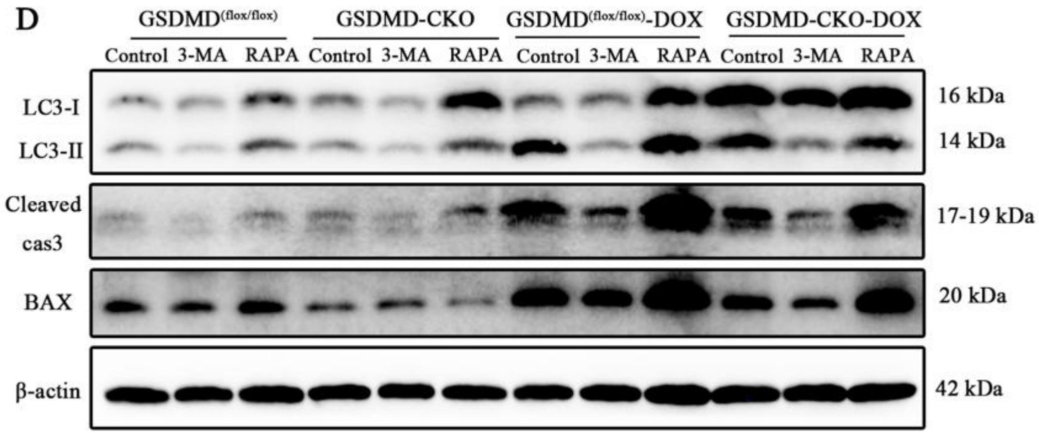
D
GSDMD(flox/flox)
GSDMD-CKO
GSDMD(flox/flox)-DOX
GSDMD-CKO-DOX
Control 3-MA RAPA Control 3-MA RAPA Control 3-MA RAPA Control 3-MA RAPA
LC3-I
LC3-II
Cleaved cas3
BAX
β-actin
16 kDa
14 kDa
17-19 kDa
20 kDa
42 kDa

LC3

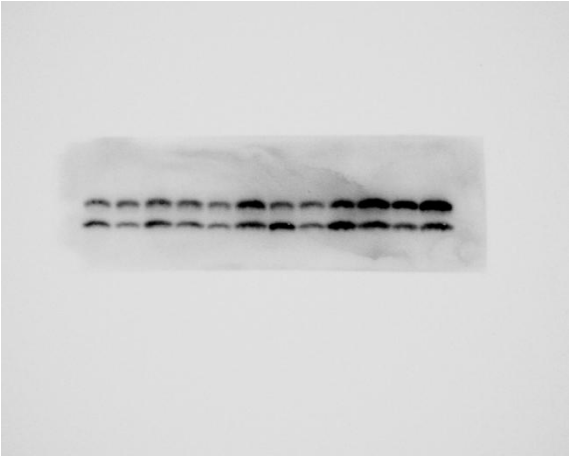

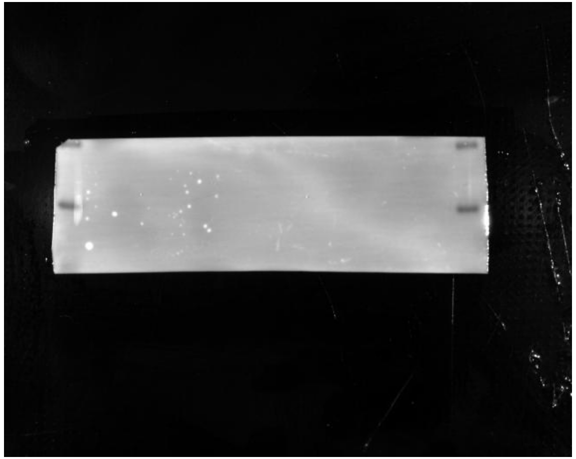

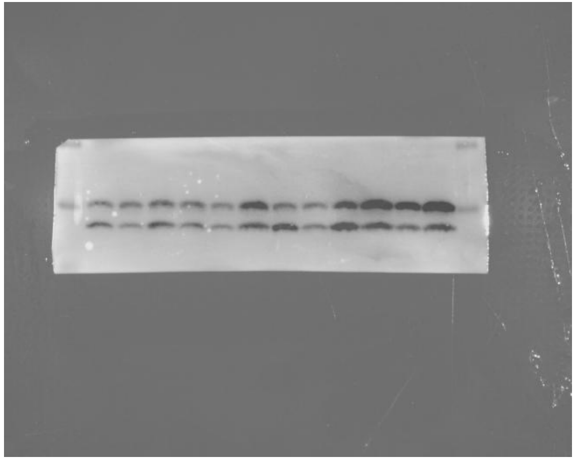

CC3

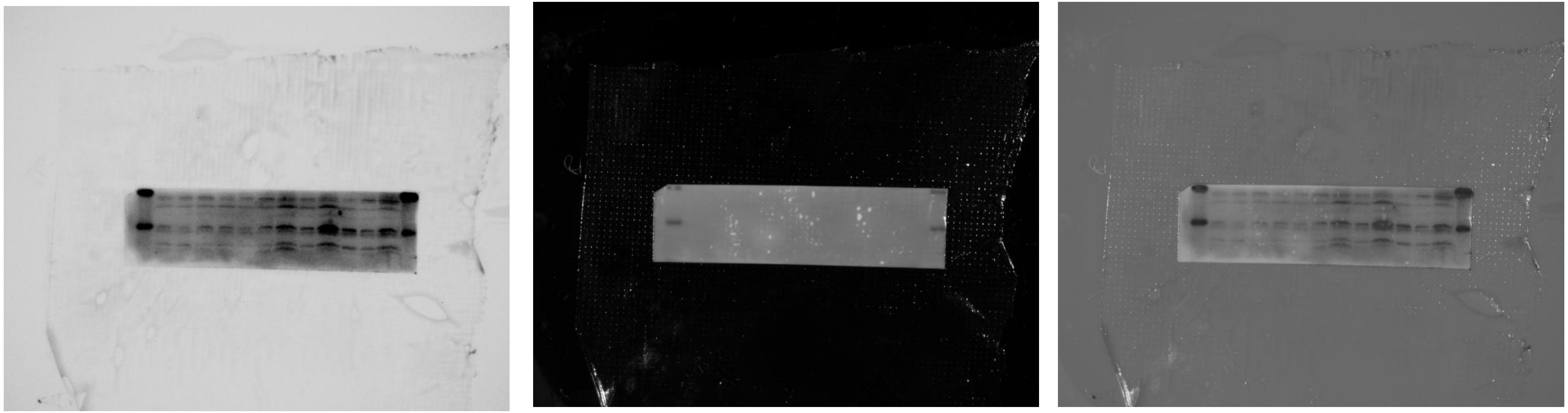

BAX

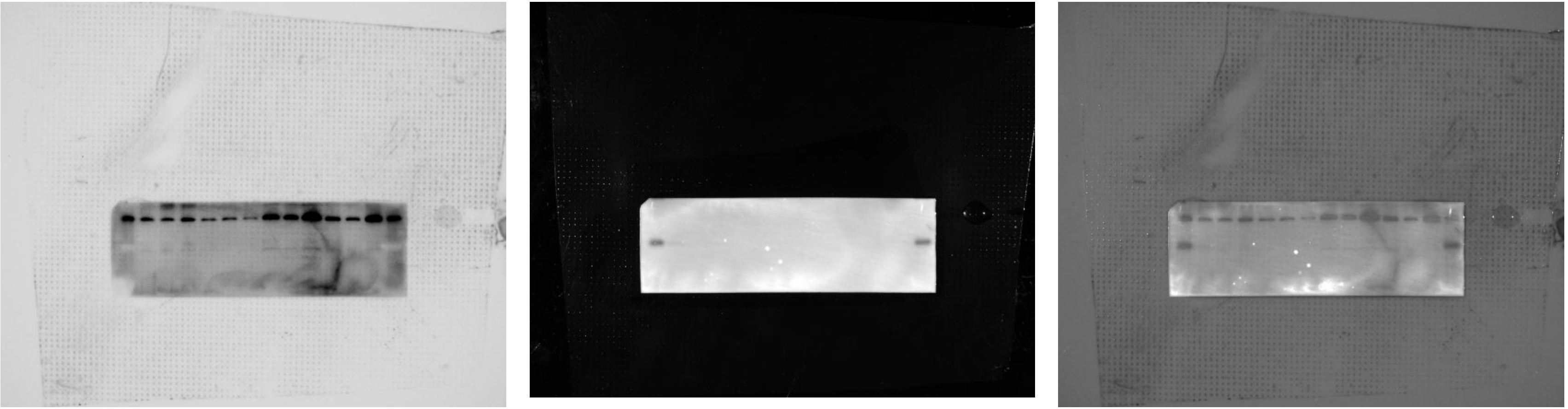

β-actin

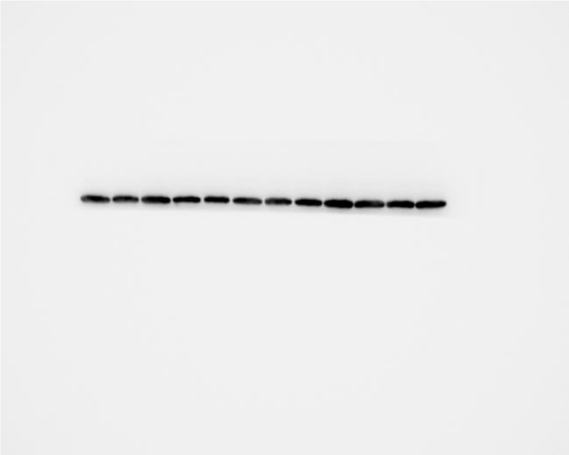

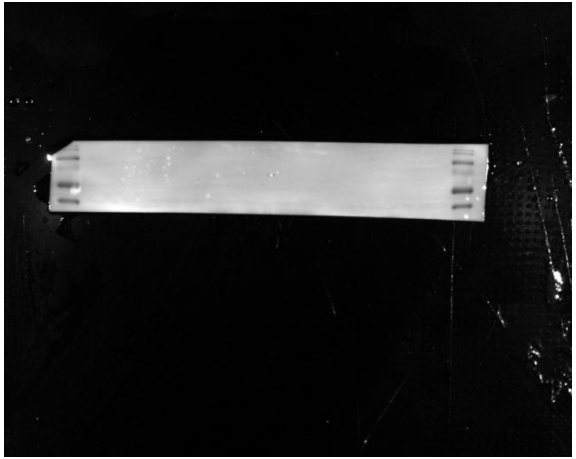

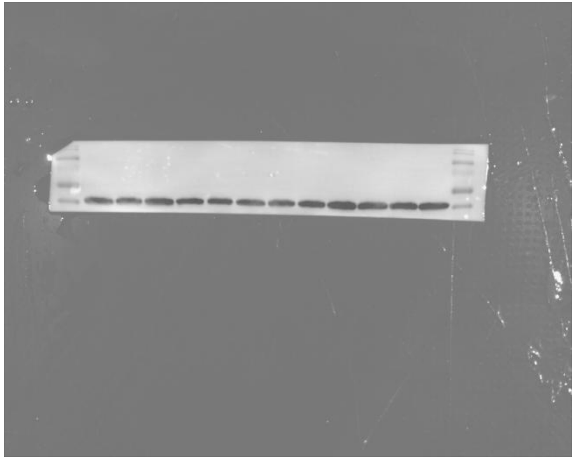

Supplementary Figure 11

A

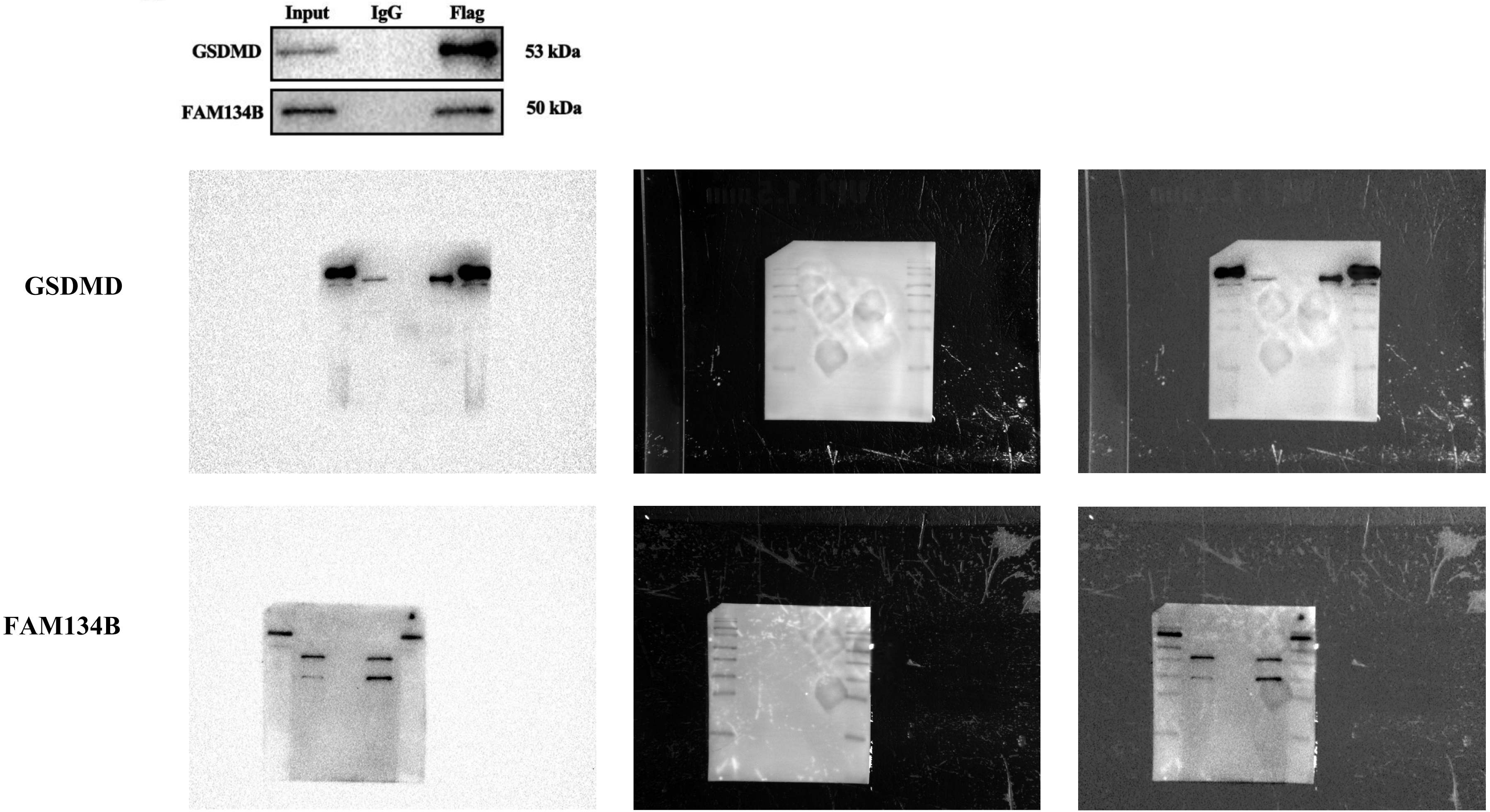

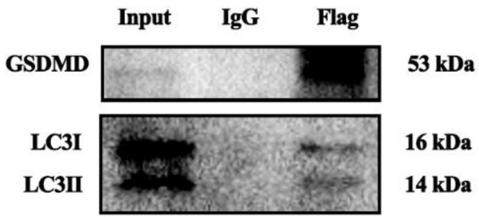

GSDMD

LC3

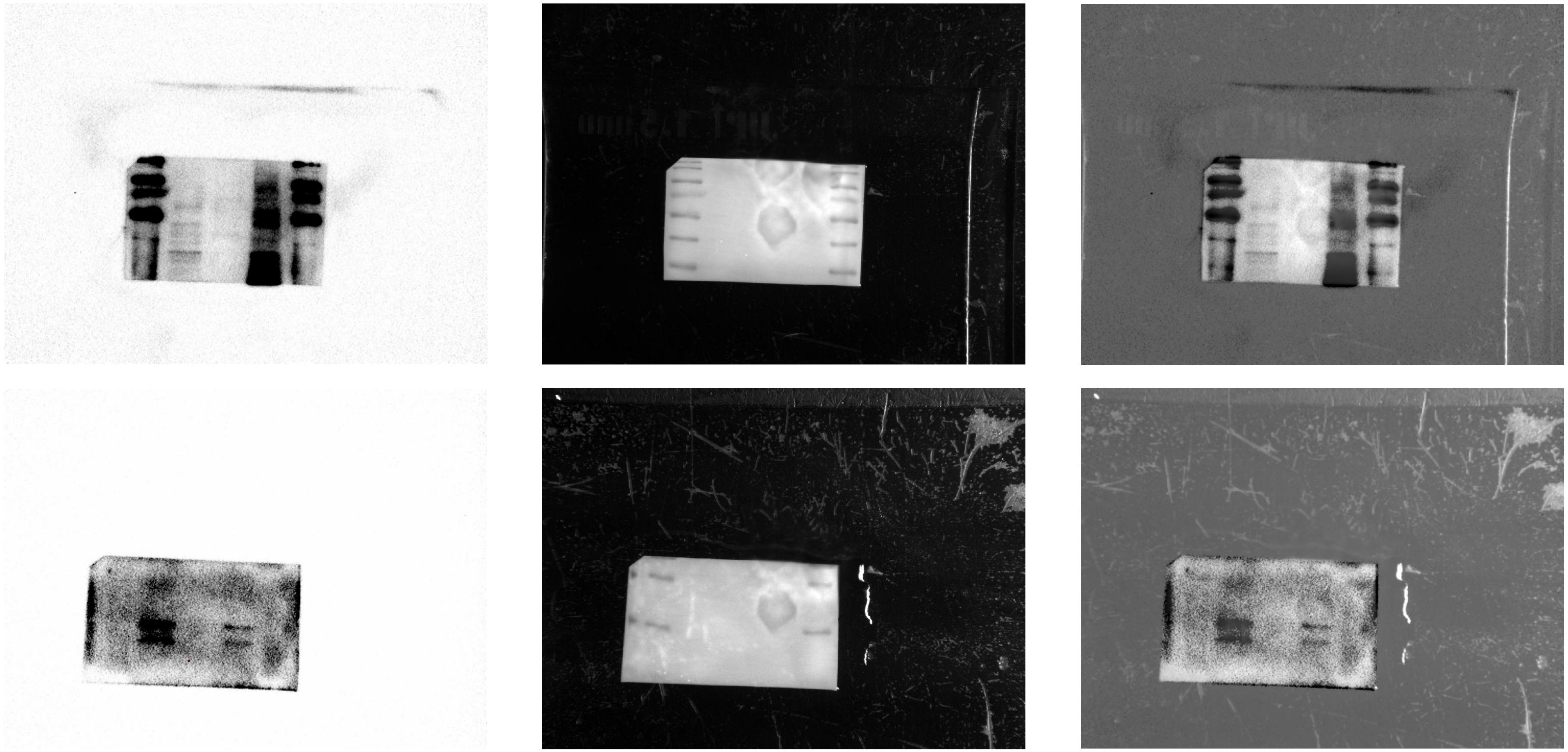

B

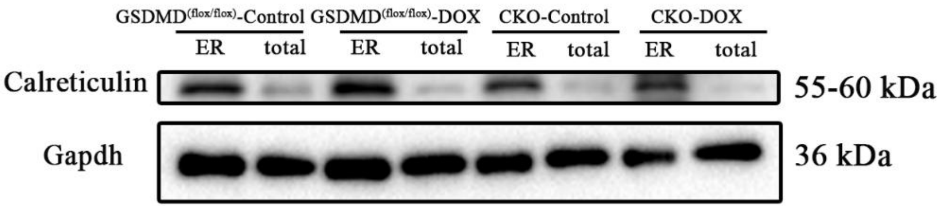

Calreticulin

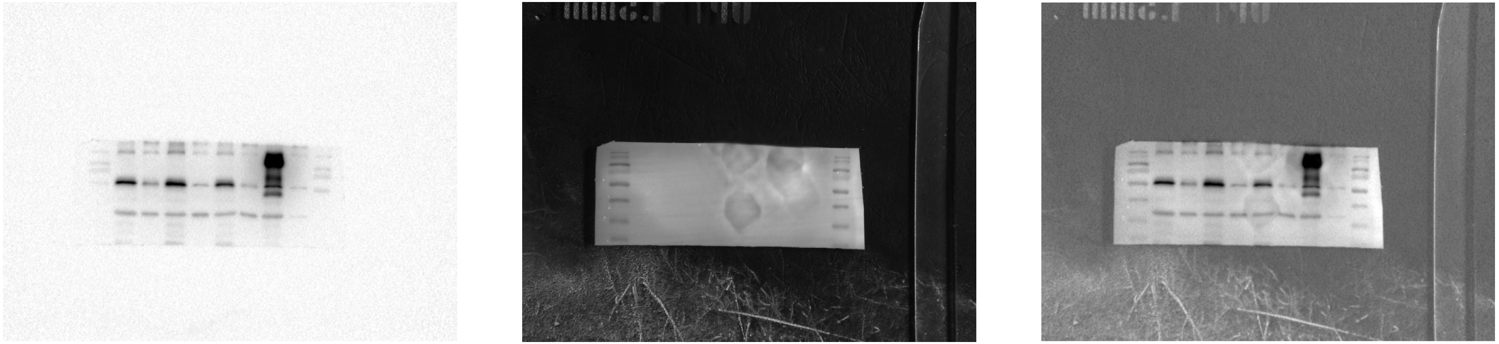

Gapdh

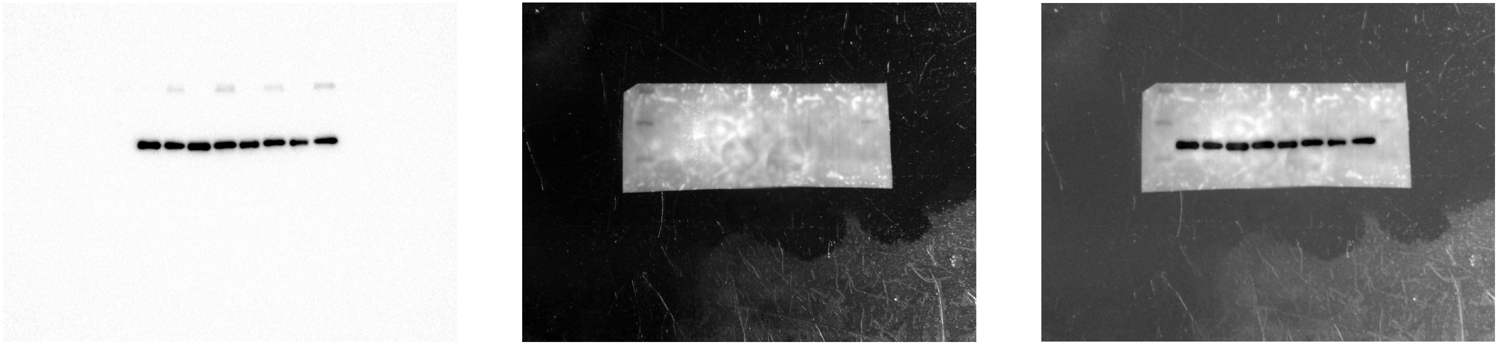

C

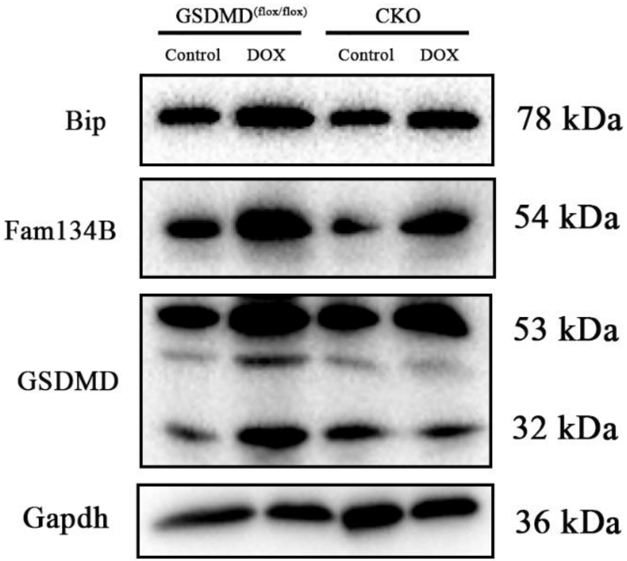
GSDMD(flox/flox)
CKO
Control
DOX
Control
DOX
Bip
Fam134B
GSDMD
Gapdh
78 kDa
54 kDa
53 kDa
32 kDa
36 kDa

Bip

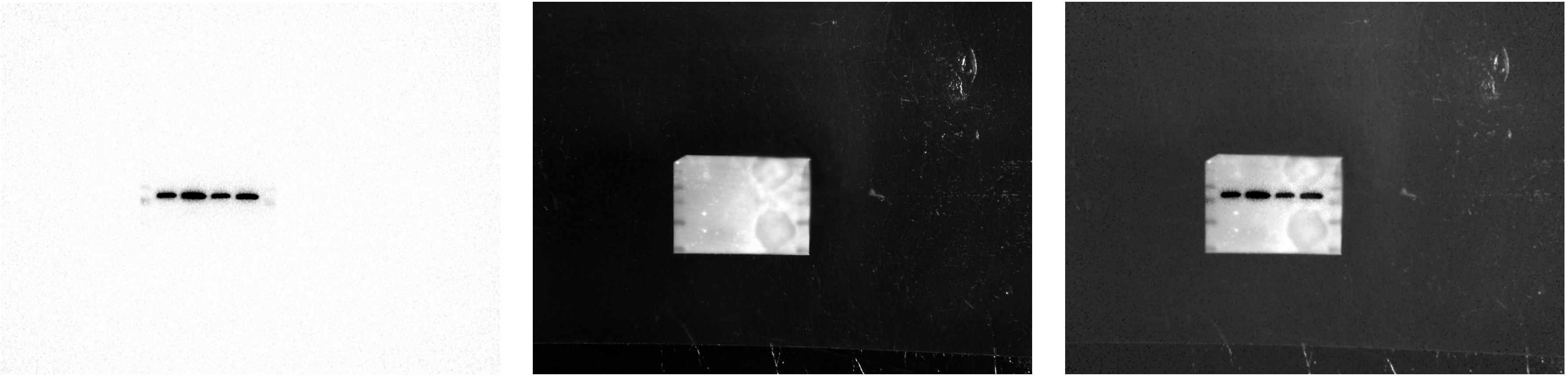

FAM134B

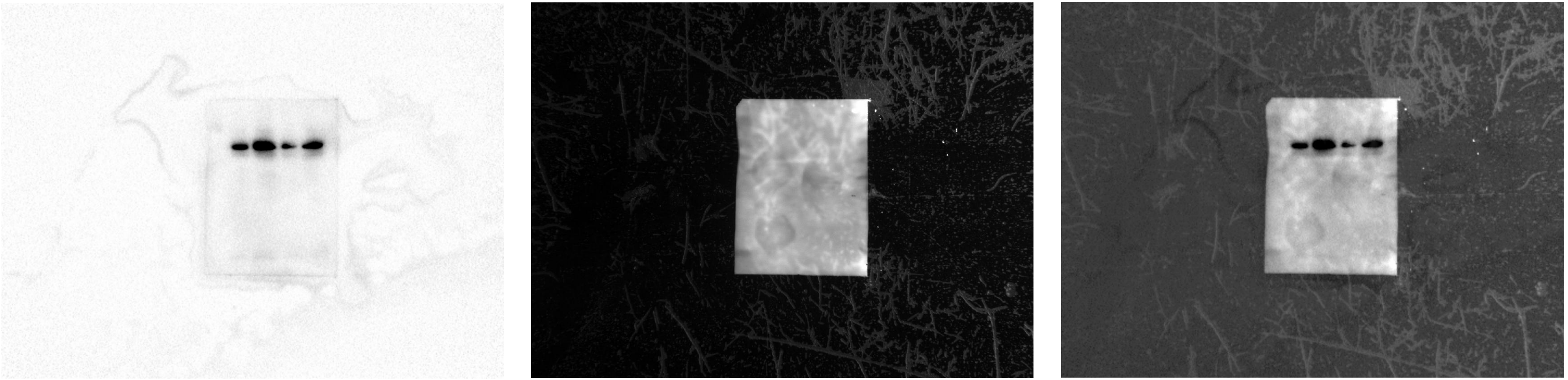

GSDMD

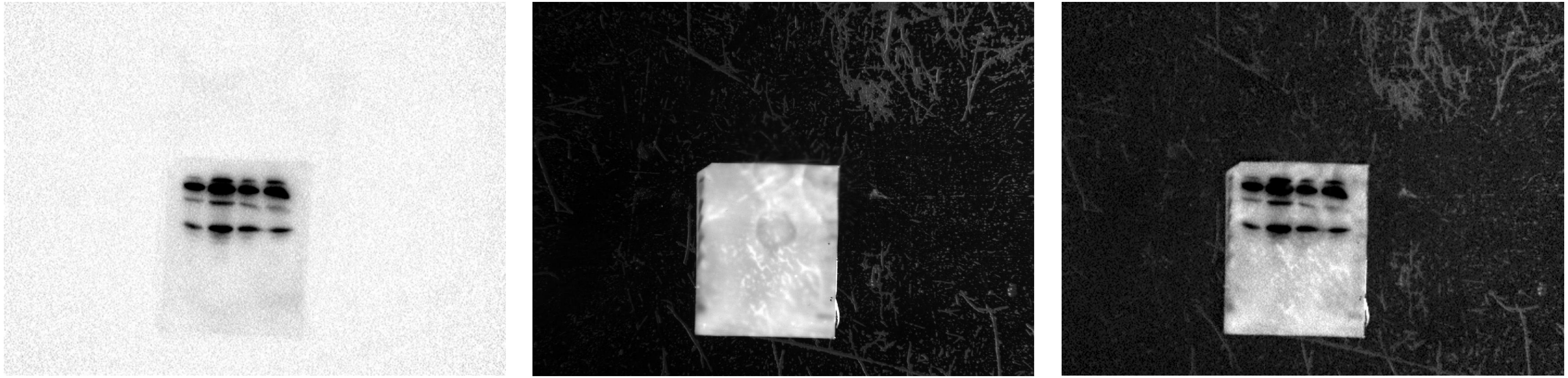

Gapdh

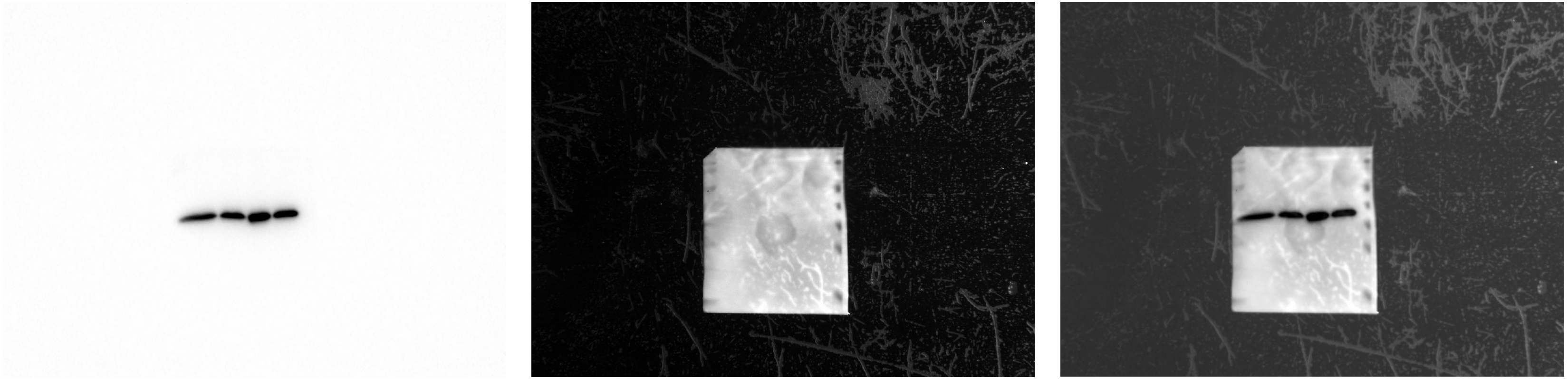

Supplement: Supplementary file 1 — WB(full length) [file 41419_2022_5333_MOESM1_ESM.pdf]
