# Supplementary material for: Gasdermin D mediates endoplasmic reticulum stress via FAM134B to regulate cardiomyocyte autophagy and apoptosis in doxorubicin-induced cardiotoxicity
Source: Cell Death Dis. 2022 Oct 26;13(10):901. doi: 10.1038/s41419-022-05333-3 (PMC9606128; doi:10.1038/s41419-022-05333-3)
Supplement: Supplementary file 14 — supplementary figure legends [file 41419_2022_5333_MOESM14_ESM.docx]

**Supplementary Figure Legends**

**Supplementary Figure 1. GSDMD is essential for myocardial I/R injury and myocardial infarction**

**A,B** Western blotting detection and quantification of GSDMD-FL, GSDMD-N, Cleaved-caspase 3 (Cleaved cas3), BAX, IL-18, IL-1β, and β-actin proteins expressions in the heart of control and I/R mice. N=6 per group. **C,D** Western blotting detection and quantification of GSDMD-FL, GSDMD-N, Cleaved-caspase 3 (Cleaved cas3), BAX, IL-18, IL-1β, and β-actin proteins expressions in the heart of control and MI mice. N=6 per group. **E-H** Representative echocardiography photographs depicting left ventricular function in the different groups of mice and analysis of the effects of GSDMD knockout on parameters of cardiac function in I/R and MI mice. N=6-7 per group. **I** LDH (lactate dehydrogenase) release in cardiomyocytes in I/R and MI groups. N=7-11 per group. **J** I/R or MI-induced reactive oxygen species formation (by dihydroethidium [DHE] staining). Scale bar, 200 μm. N=3 per group. **K** Representative images of TUNEL stainings in I/R and MI hearts. Scale bar, 200 μm. N=3 per group. Data are depicted as the mean ± SEM. Statistical significance was determined by one-way and two-way ANOVA with a post-hoc Holm-Sidak test, ns, not significant; *P<0.05; **P<0.01; ***P<0.001; ****P<0.0001.

**Supplementary Figure 2. DOX treatment exacerbates cardiac injury**

**A** Cumulative survival rates of control and DOX-treated groups. N=10 per group. **B** Heart weight/tibia length ratios (HW/TL) and body weights (BW) were measured at 7 days after DOX administration. N=7-9 per group. **C** Plasma levels of CK-MB and cTnT, respectively, measured by Elisa assay. N=4-6 per group. **D** Representative images of echocardiographs and statistics of heart rate (HR), ejection fraction (EF) and fractional shortening (FS). N=10 per group. **E,F** Resting cell length, maximal velocity of relengthening (+dL/dt), maximal velocity of shortening (-dL/dt), peak shortening (PS, normalized to resting cell length), Time-to-PS (TPS), Time-to-90% relengthening (TR90). **G,H** Protein levels of Cleaved-caspase 3 (Cleaved cas3), BAX, BCL-2 using western blot assay. N=6 per group. **I** The cardiac myocyte sizes measured by wheat germ agglutinin (WGA) and epifluorescence microscopy of reactive oxygen species (ROS). Scale bar, 100 μm for WGA and 200 μm for ROS. N=11 for WGA groups and n=12 for ROS groups. **J** Apoptosis of myocardium co-stained with cTnT determined by TUNEL staining. Scale bar, 200 μm. **K** Protein levels of GSDMD-FL and GSDMD-N using western blot assay. N=6 per group. **L** The graph of TUNEL staining. N=11 per group. Statistical significance was determined by student’s t test or one-way ANOVA with a post-hoc Holm-Sidak test. Data are depicted as the mean ± SEM, ns, not significant; *P<0.05; **P<0.05; ***P<0.001; ****P<0.0001.

**Supplememtary Figure 3. DIC injury in cardiomyocytes mediated by the Caspase 11-GSDMD-N pathway**

**A** Western blotting detection and quantification of Cleaved-caspase 1 (Cleaved CASP1), GSDMD, and β-actin proteins expressions in cardiomyocytes. N=6 per group. **B** Western blotting detection and quantification of Cleaved-caspase 3 (Cleaved CASP3), GSDMD, and β-actin proteins expressions in cardiomyocytes. N=6 per group. **C** Western blotting detection and quantification of Cleaved-caspase 11 (Cleaved CASP11), GSDMD, and β-actin proteins expressions in cardiomyocytes. N=6 per group. Data are depicted as the mean ± SEM. Statistical significance was determined by one-way and two-way ANOVA with a post-hoc Holm-Sidak test, ns, not significant; *P<0.05; **P<0.01; ***P<0.001; ****P<0.0001.

**Supplememtary Figure 4. Cardiomyocyte pyroptosis mainly occurred in the early stage of DIC and apoptosis occurred in the late stage**

**A,B** Temporal changes in GSDMD-FL, GSDMD-N, Cleaved-caspase 3 (Cleaved cas3), and BAX protein levels after 1 dose of DOX. Cardiomyocytes were harvested at different time points (0h, 3h, 6h, 12h, 24h, 48h) after treated with DOX, and 0 h group served as the control. N=4 per group. **C,D** LDH (lactate dehydrogenase) release and IL (interleukin)-18 levels in supernatants varies with time as measured by ELISA. N≥5 per group. **E** IL-18 levels and LDH release in cardiomyocytes treated with DOX for 12 h. N≥5 per group. Data are depicted as the mean ± SEM. Statistical significance was determined by one-way and two-way ANOVA with a post-hoc Holm-Sidak test, ns, not significant; *P<0.05; **P<0.01; ***P<0.001; ****P<0.0001.

**Supplememtary Figure 5. Cardiomyocytes die by apoptosis after DOX treatment**

**A,B** Dynamic changes of cardiomyocytes treated with DOX at different time points (0h, 18h, 21h, 23h, 24h) showed by the Lionheart FX living cell imaging analysis system.

**Supplememtary Figure 6. A large number of cardiomyocytes died via apoptosis with the prolongation of DOX treatment time**

**A,B** Dynamic changes of two cardiomyocytes from adult mice treated with DOX for 12 h observed by confocal microscopy.

**Supplememtary Figure 7. Deficiency of GSDMD alleviates DOX-induced cardiotoxic injury**

**A,B** Body weights (BW) and heart weight/tibia length ratios (HW/TL) in WT and GSDMD-KO mice. N=8-10 per group. **C,D** Statistics of heart rate (HR) and fractional shortening (FS) of WT and GSDMD-KO mice treated with DOX or not. N=7-9 per group. **E** Representative images of echocardiographs of WT and GSDMD-KO mice. **F-H** Resting cell length, maximal velocity of relengthening (+dL/dt), maximal velocity of shortening (-dL/dt), peak shortening (PS, normalized to resting cell length), Time-to-PS (TPS), Time-to-90% relengthening (TR90). **I** Cell sizes measured by WGA staining, compared with WT-control group. N=10 per group. **J** Quantitative data of epifluorescence microscopy of control and DOX-treated hearts in WT and KO mice for ROS. N=11 per group. **K,L** Analysis of GSDMD (FL and N teminals), Cleaved-caspase 3, BAX, IL-1β, and IL-18 proteins levels in control and GSDMD-KO hearts. N=6 per group. **M** Quantification of TUNEL-positive nuclei per total nuclei. N=11 per group. Data are depicted as the mean ± SEM. Statistical significance was determined by one-way and two-way ANOVA with a post-hoc Holm-Sidak test, ns, not significant; *P<0.05; **P<0.01; ***P<0.001; ****P<0.0001.

**Supplememtary Figure 8. Overexpression of GSDMD aggrevates DOX-induced cardiotoxic injury**

**A,B** Body weights (BW) and heart weight/tibia length ratios (HW/TL) in AAV9-NC and AAV9-GSDMD-OE mice. N=8-9 mice per group. **C** Analysis of heart rate and fractional shortening in AAV9-NC and AAV9-GSDMD-OE mice. N=8-10 per group. **D** Representative images of echocardiographs of mice infected with AAV9-NC or AAV9-GSDMD-OE with or without DOX treatment. **E,F** Resting cell length, maximal velocity of relengthening (+dL/dt), maximal velocity of shortening (-dL/dt), peak shortening (PS, normalized to resting cell length), Time-to-PS (TPS), Time-to-90% relengthening (TR90) of AAV9-NC and AAV9-GSDMD-OE mice. **G,H** Analysis of cell sizes and reactive oxygen species production levels measured by WGA and dihydroethidium (DHE) staining, respectively. N=10 for WGA groups and n=11 for ROS groups. **I,J** Analysis of GSDMD (FL and N teminals), Cleaved-caspase 3 (Cleaved cas3), and BAX proteins levels in AAV9-NC and AAV9-GSDMD-OE mice. N=6 per group. **K** Quantification of TUNEL-positive nuclei per total nuclei in AAV9-NC and AAV9-GSDMD-OE mice. N=10 per group. Data are depicted as the mean ± SEM. Statistical significance was determined by one-way and two-way ANOVA with a post-hoc Holm-Sidak test, ns, not significant; *P<0.05; **P<0.01; ***P<0.001; ****P<0.0001.

**Supplememtary Figure 9. Cardiomyocyte-specific GSDMD deficiency alleviates acute and chronic DIC.**

**A** Analysis of heart rate and fractional shortening in GSDMD^(flox/flox)^ and CKO mice in acute DIC. N=6 per group. **B** Kaplan-Meier survival analysis showing improved survival in CKO mice compared with GSDMD^(flox/flox)^ mice both in control and DOX-treated groups after 6 weeks follow-up. N≥10 per group. **C** Body weight change at different time point (2W, 4W, 6W after DOX injfection) in four groups. N=6-10 per group. **D** Heart weight/tibia length ratios (HW/TL) of GSDMD^(flox/flox)^ and CKO mice 6 weeks after saline or DOX administration. N=8-10 per group. **E,F** Representative images of echocardiographs of GSDMD^(flox/flox)^ and GSDMD-CKO mice in baseline and 2W, 4W, 6W after DOX treatment. Statistics of heart rate, ejection fraction and fractional shortening at different times. N≥6 per group. **G,H** Representative western blot data and quantitative analysis of Cleaved-caspase 3 (Cleaved cas3) and BAX from vehicle-treated control and DOX-treated hearts in the absence and presence of cardiac GSDMD. N=6 per group. Data are depicted as the mean ± SEM. Statistical significance was determined by one-way and two-way ANOVA with a post-hoc Holm-Sidak test, ns, not significant; *P<0.05; **P<0.01; ***P<0.001; ****P<0.0001.

**Supplementary Figure 10. GSDMD aggregates DIC by promoting myocardial autophagy**

**A** Body weight (BW) changes and heart weight/tibia length ratios (HW/TL) in GSDMD^(flox/flox)^ and CKO mice treated with DOX, 3MA, RAPA or not. N=6 per group. **B,C,E,F** Representative images of echocardiographs with different treatments (DOX, 3MA, RAPA) in GSDMD^(flox/flox)^ and GSDMD-CKO mice. Statistics of heart rate, ejection fraction (EF) and fractional shortening (FS) in various groups. N=6 per group. **D,G-I** Representative western blot and analysis of LC3, Cleaved caspase 3 (Cleaved cas3), and BAX protein levels in GSDMD^(flox/flox)^ and CKO mice treated with DOX, 3MA, RAPA or not. N=3 per group. Data are depicted as the mean ± SEM. Statistical significance was determined by one-way and two-way ANOVA with a post-hoc Holm-Sidak test, ns, not significant; *P<0.05; **P<0.01; ***P<0.001; ****P<0.0001.

**Supplementary Figure 11. GSDMD promotes autophagy by promoting ERS to activate FAM134B, thus aggravating cardiomyocyte apoptosis**

**A** Full-length Flag-GSDMD protein was transiently expressed in cardiomyocytes. Interaction between GSDMD and FAM134B, GSDMD and LC3 in cardiomyocytes were determined by coimmunoprecipitation. **B** ER proteins extracted from GSDMD^(flox/flox)^ and CKO mice. **C,D** Representative western blot and analysis of Bip， Fam134B, and GSDMD protein levels in ER. N=3 per group. Data are depicted as the mean ± SEM. Statistical significance was determined by one-way and two-way ANOVA with a post-hoc Holm-Sidak test, ns, not significant; *P<0.05; **P<0.01; ***P<0.001; ****P<0.0001.

**Supplementary Figure 12. GSDMD attaches to the ER for pore formation under**

**DOX treatment**

**A** GSDMD and Bip are colocalized in adult mice cardiomyocytes as shown by immunofluorescence after DOX treatment or not. scale bar: 40 μm or 10 μm. **B** Representative electron micrograph images and analysis of adult mice cardiomyocytes treated with DOX or not. The red arrow showing pore forming on the ER membrane. scale bar: 500 nm. Data are depicted as the mean ± SEM. Statistical significance was determined by one-way and two-way ANOVA with a post-hoc Holm-Sidak test, ns, not significant; *P<0.05; **P<0.01; ***P<0.001; ****P<0.0001.

**Supplementary Materials table 1:** Mouse primers used in this study.
