# Supplementary material for: Gasdermin D mediates endoplasmic reticulum stress via FAM134B to regulate cardiomyocyte autophagy and apoptosis in doxorubicin-induced cardiotoxicity
Source: Cell Death Dis. 2022 Oct 26;13(10):901. doi: 10.1038/s41419-022-05333-3 (PMC9606128; doi:10.1038/s41419-022-05333-3)
Supplement: Supplementary file 15 — supplementary materials [file 41419_2022_5333_MOESM15_ESM.docx]

**Supplemental Methods**

**Antibodies**

The following antibodies were used for western blotting or coimmunoprecipitation: anti-GSDMD (NBP2-33422, Novus Biologicals; ab209845, Abcam), anti-IL-18 (ab207323, Abcam), anti-IL-1β (ab234437, Abcam), anti-LC3 (ab221794, Abcam), P62 (ab109012, Abcam), anti-cleaved caspase3 (ab214430, Abcam), anti-BAX (2772S, Cell Signaling Technology), anti-BCL-2 (ab196495, Abcam), anti-Bip (A0241, Abclonal; ab21685, Abcam), anti-Hsp 90 (ab109248, Abcam), anti-FAM134B (ab151755, Abcam; 83414S, Cell Signaling Technology), anti-caspase-11 (ab18741, Abcam), anti-caspase-1 (A0964, Abclonal), anti-Flag antibody (14793S, Cell Signaling Technology), and anti-β-actin (4970S, Cell Signaling Technology). The following antibodies were used for immunostaining and immunofluorescence: anti-cTnT (ab8295, Abcam), anti-GSDMD (A18281, Abclonal; 66387-1-lg, Proteintech), anti-Bip (ab21685, Abcam), anti-FAM134B (83414S, Cell Signaling Technology), and anti-LC3 (13082S, Cell Signaling Technology; 98557S, Cell Signaling Technology).

**Animals**

Wild-type (WT) C57BL/6J mice (male, 6–8-week-old) were purchased from Jie Si Jie Laboratory Animal Corp. (Shanghai, China). Mice with GSDMD deletion in C57BL/6J background were generated as described previously [1]. The cardiac-specific GSDMD knockout (GSDMD-CKO) mice were generated via the GSDMD^flox/flox^ strain using the Myh6-Cre strain (Cyagen Biosciences Inc., Guangzhou, China), as previously described^1^. The recombinant adeno-associated virus (AAV) vector was constructed by OBiO Technology (Shanghai, China) using a full-length GSDMD complementary DNA (cDNA) sequence to overexpress GSDMD. To induce exogenous GSDMD expression in vivo, mice were injected with the AAV9-GSDMD virus (1×10^9^ PFU/ml per mouse) via tail vein 3 d before doxorubicin (DOX) administration. Mice were randomized into treatment group or control group. There were no differences between groups at baseline. Only male mice were used in all experiments to avoid the cardiovascular protective effects of estrogen in female mice. The mice were euthanized before killing 2% isoflurane. The organs of killed mice were harvested and weighed to compare the body weight change (BW, g) and heart weight/tibia length (HW/TL, mg/mm) ratios of KO, CKO, GSDMD^(flox/flox)^, and WT mice. The survival curves were also compared between the groups.

At the indicated time points, mice were anesthetized using isoflurane (3%, isoflurane, 1 L/min oxygen), and after verification of deep anesthesia, they were euthanized by exsanguination and pneumothorax. Tissues were subsequently collected for analysis. All animals were cared for in accordance with the National Institutes of Health Guidelines for the Care and Use of Laboratory Animals and all protocols were approved by the Animal Care and Use Committee of Zhongshan Hospital, Fudan University.

**In Vivo Model of Acute and Chronic DOX Cardiomyopathy**

DOX (20 mg/kg, Sigma, 25316-40-9, USA) was dissolved in normal saline (NS) and injected intraperitoneally, once, into 6–8-week-old GSDMD-deficient male mice and their WT littermates to induce acute cardiotoxic injury. Control groups were injected with the same volume of NS. Chronic DOX-induced cardiotoxicity (DIC) was established in WT and GSDMD-deficient mice by intraperitoneally injecting DOX (5 mg/kg, dissolved in NS) once weekly for 6 weeks. Typically, mice were anaesthetized by isoflurane inhalation and the left ventricle (LV) was dissected for analysis 7 d after DOX administration in acute DIC and after 6 weeks of DOX administration in the chronic model.

**In Vitro Model of DOX Cardiomyopathy**

Adenoviruses containing the GSDMD gene (GSDMD-OE) and a negative control (OE-C) were obtained from OBiO Technology (China, Shanghai). To establish GSDMD-overexpressing cardiomyocytes in vitro, cardiomyocytes were isolated from 6-to 8-week-old C57BL/6J mice and transfected with adenovirus vectors carrying the mouse GSDMD gene (GSDMD-OE) (MOI=10, OBiO Technology) or the corresponding control sequences (OE-C) for at least 24 h. The cells were then cultured in fresh culture medium containing DOX (1 μM) for additional 12 h. Transfection efficiency was validated by western blotting and real-time PCR.

The GV119 vector was used to construct adenoviral short hairpin RNAs (shRNAs). DNA sequences encoding shRNAs for caspase-1 (Ad-sh-caspase-1,5’-TTTCTTAACGGATGCAATT-3’), caspase-3 (Ad-sh-caspase-3, 5′-CAACGGAATTCGAGTCCTT-3′), caspase-11 (Ad-sh-caspase-11, 5’-GTACACGAAAGGCTCTTAT-3’), and a scrambled control (Ad-sh-NC, 5’-TTCTCCGAACGTGTCACGTAA-3’) were cloned into the GV119 vector (Gateway System; Invitrogen, Carlsbad, USA). The specific methodology used was as described previously [1,2].

**Cell Isolation, Culture, and Treatment**

To isolate primary adult cardiomyocytes, C57BL/6J mice (male, 6–8-week-old) or mice with GSDMD deletion and GSDMD-CKO mice were injected intraperitoneally with DOX (20 mg/kg; Sigma, 25316-40-9). Adult cardiomyocytes were isolated 7 d later. First, the mice were anesthetized with 2% isoflurane and the chest was opened. The descending aorta was cut and the base of the right ventricle (RV) was manually perfused for approximately 1 min with 7 ml of EDTA buffer (pH 7.8) to remove as much blood as possible. The heart was removed and transferred to a 60-mm dish containing EDTA. Next, the heart was sequentially placed in 10 ml of EDTA buffer (for approximately 6 min), 3 ml of perfusion buffer (for approximately 2 min), and 25–50 ml of collagenase buffer (for approximately 20 min). The heart was then pulled apart into roughly 1 mm × 1 mm pieces using forceps. After complete digestion, enzyme activity was inhibited by the addition of 5 ml of stop buffer and the cells were filtered through a 100 μm cell strainer. Cells were collected by gravity settling for 20 min, and then sequentially resuspended in three calcium reintroduction buffers to achieve healthy populations of calcium-tolerant cells. The cells were resuspended in prewarmed plating medium, when required, and cultured at 37 °C in an incubation box with 5% CO_2_ atmosphere. After 1 h, cardiomyocytes were suspended in culture medium. The medium was changed every 48 h. The culture medium was M199, supplemented with 5% Bovine serum albumin (BSA), 100× ITS, 1 mol/L BDM, 100× CD lipid, and 100× penicillin/streptomycin solution.

**Mechanical Properties of Cardiomyocytes**

The contraction and relaxation of single cardiomyocyte were measured using an IonOptix^TM^ system, as previously reported [3,4]. Cell shortening and relengthening were determined by using the following indicators: peak shortening (PS), time to PS (TPS), time to 90% relengthening (TR90), and maximal velocities of shortening/relengthening (+/- dL/dt).

**Microscopic Imaging of Cell Death**

Cardiomyocytes were cultured in 6-well plates (Corning, NY, USA) in culture medium. DOX (1 μM) was added at the start of the observation, which lasted 24 consecutive hours. Cell death, especially apoptosis, was visualized by continuous live imaging and captured every half hour by the Lionheart FX living cell imaging analysis system (BioTek, Winooski, VT, USA). At least three random fields were selected from each well.

**Detection of Reactive Oxygen Species (ROS)**

ROS measurements were performed in heart tissues or cardiomyocytes after DOX treatment. Frozen sections or cells were stained with dihydroethidium (DHE, S0033; Beyotime) at 37 °C for 30 min in the dark, washed three times with Phosphate buffered saline (PBS, pH 7.4), and sealed with 4′,6-diamidino-2-phenylindole (DAPI, C1006, Beyotime) staining solution at room temperature for 10 min in the dark. The slides were then washed with PBS (pH 7.4) and sealed with anti-fluorescence quenching sealing tablets. Staining intensity was detected by using a confocal microscope (Olympus FV3000, Japan).

**Autophagy Assay**

To induce autophagic flux in vivo or in vitro, mice or cardiomyocytes, respectively, were treated with bafilomycin A1 (BAFA1) (1.5 mg/kg, intraperitoneal injection; Selleck.cn, S1413) or NS for 2 h before euthanizing [5]. Heart tissues were lysed, and prepared for the detection of LC3II and p62 proteins. 3-Methyladenine (3-MA, 1.5 mg/100 g, intraperitoneal injection; Once every two days, a total of 3 times; HY-19312, MedChemExpress) or rapamycin (Rapa, 2.0 mg/kg, intraperitoneal injection; every day; AY-22989, MedChemExpress) were administered in the acute model from its setup to inhibit or induce autophagy, respectively.

**Analysis of Fluorescent LC3 Puncta**

Fluorescence of mRFP-GFP-LC3 was visualized in cardiomyocytes as previously described^4^. Briefly, to detect autophagic flux, cardiomyocytes were infected with mRFP-GFP-LC3 adenovirus (10^6^ PFU/ml, Hanbio Biotechnology Co., Ltd.) for 24 h. Then, the cells were treated by DOX (1 μM) for 12 h to induce autophagic flux. The cells were washed with PBS (pH 7.4) and visualized using a confocal laser-scanning microscope (Olympus FV3000, Japan). Fluorescent mCherry and GFP puncta were counted manually using ImageJ software (1.52a, NIH, USA).

**Chemical Chaperones Known to Suppress ER Stress Signaling**

One day before receiving doxorubicin, saline or 4-phenylbutyrate (100 mg/kg per day) was administered once a day for 1 week by intraperitoneal injection. Thereafter, the mice were subjected to echocardiography, and heart samples were obtained for Western blot analyses.

**ER Fractionation**

ER isolation was performed using a Minute ER Enrichment Kit (Invent Biotechnologies) according to the manufacturer’s instructions. The specific methodology used was as described previously [6].

**RNA Sequencing (RNA-seq)**

To evaluate changes on the transcriptome level associated with GSDMD deficiency, RNA was isolated from the LV of DOX-treated GSDMD^(flox/flox)^ and GSDMD-CKO mice. The specific methodology used was as described previously [7].

**Enzyme-linked Immunosorbent Assay (ELISA)**

Mouse serum samples were collected and stored at –80 ℃. The serum concentration of CK-MB (MAK116, Sigma, St. Louis) and cTnT (MAB18742-100, Novus Biologicals) and the IL-18 (EM3180S, Wellbio) levels of cardiomyocytes supernatants were determined using specific ELISA kits, in accordance with the manufacturer’s instructions, in 96-well plates. Sample absorbance in each well was determined at 450 nm.

**Cell Viability Assays**

Lactate dehydrogenase (LDH) release into the culture medium was measured by spectrophotometry using a specific kit (MAK066, Sigma, St. Louis, USA), according to the manufacturer’s instructions.

**Transmission Electron Microscopy (TEM)**

TEM was used to observe autophagosomes, mitochondria, and the bubbling phenomenon. Cardiomyocytes were collected by centrifugation and the medium was discarded. The cells were then fixed in 2.5% glutaraldehyde at 4 ℃ for 15 min. Next, the cells were scraped off, centrifuged, and stored at 4 ℃ for at least 4 h. They were then rinsed three times in 0.1 M phosphate buffer (pH 7.2), fixed at room temperature (20 ℃) for 2 h, and rinsed again. Next, the cells were passed through an alcohol gradient dehydration series and permeabilized in acetone and epoxy resin (2:1, 1:1, and only epoxy resin) for 8–12 h at 37 ℃ each time. The permeabilized samples were embedded in epoxy resin and cut into 80–100 nm-thick slices by using ultr-thin slicer (Leica UC7, DE). Finally, the samples were double stained led by uranium and observed under an electron microscope (Tecnai G20 TWIN, FEI, USA).

**Immunostaining and Immunofluorescence Detection**

Paraffin sections were dewaxed in water by using xylene and alcohol, and then repaired in a microwave oven using an antigen repair buffer with EDTA (pH 8.0), before washing with PBS (pH 7.4). A histochemical pen was used to draw around the tissue, self-fluorescence quenching agent was added for 5 min, and the tissue was rinsed with water for 10 min. BSA was added for 30 min. The sections were then incubated with a primary antibody at 4 ℃ overnight. Then, the sections were washed with PBS (pH 7.4) and incubated with a secondary antibody at room temperature for 50 min. The nuclei were counterstained with DAPI (C1006, Beyotime) for 10 min in the dark at room temperature. The sections were washed using PBS (pH 7.4) and sealed with anti-fluorescence quenching sealing tablets (S2110, Solarbio, BJS CHN) before storing at 4 ℃ in a light-proof box. The stained slides were photographed with a confocal microscope (Olympus FV3000, Japan).

**Coimmunoprecipitation (CO-IP)**

The specific methodology used was as described previously [8]. Briefly, Full-length Flag-GSDMD protein was transiently expressed in primary adult cardiomyocytes. Then cardiomyocytes were treated with 1 μM DOX for 12 h. Coimmunoprecipitation using an anti-Flag antibody or control IgG was performed. Immunoprecipitants and input cell lysates were analyzed by western blotting.

**Terminal Deoxynucleotidyl Transferase dUTP Nick-end Labeling (TUNEL)**

TUNEL assay was used to detect myocardial apoptosis. The In Situ Cell Death Detection Kit (C1088, Beyotime) was used, according to the manufacturer’s instructions. The nuclei were stained with DAPI (C1006, Beyotime). TUNEL-positive cells and all cells were counted by using ImageJ software (1.52a, NIH, USA), and the percentage of apoptotic cells was calculated in a blinded manner.

**Calcein/Propidium Iodide (PI) Live/Dead Viability Test**

PI staining was performed to evaluate apoptosis using Calcein/PI Cell Viability/Cytotoxicity Assay Kit (Beyotime). Adult mouse cardiomyocytes were added to staining solution and incubated at 37 ℃ for 30 min in the dark. PI generates red fluorescence, which was detected using fluorescence microscope and quantified by using ImageJ software (1.52a, NIH, USA).

**Echocardiography**

Cardiac function before and after DOX injection was evaluated using echocardiography. Mice were anesthetized with 2% isoflurane while fixed in the supine position, and then underwent transthoracic echocardiography (VeVo 2100 Imaging System; VisualSonics, Toronto, ON, Canada). Mouse body temperature was maintained between 36.9 °C and 37.3 °C. The heart rate was maintained at 400–550 bpm. M-mode ultrasound analysis of the heart was performed. The heart rate (HR), ejection fraction (EF), fractional shortening (FS), cardiac output (CO), left ventricular end systolic diameter (LVES), left ventricular end-diastolic diameter (LVED), and stroke volume (SV) were measured and calculated using Vevo LAB 3.1.1 software.

**Western blotting**

LV tissues or cells were lysed in RIPA lysis buffer (WB0101, Biotech Well), and protein concentrations were determined using BCA Protein Assay Kit (P0010S, Beyotime). An equal amount of tissue protein (40–50 μg) or cellular protein (20-30 μg) was separated on 10–15% SDS-polyacrylamide gels by electrophoresis and then transferred to polyvinylidene difluoride membrane (ISEQ00010, Millipore). The membrane was blocked for 1 h at room temperature using 5% nonfat dried milk in TBST, and subsequently incubated overnight at 4 °C with different primary antibodies. The membrane was then washed with TBST, incubated with a corresponding horseradish peroxidase-conjugated IgG secondary antibody for 1 h at room temperature, and again washed with TBST buffer. Protein bands were visualized by using an automatic chemiluminescence imaging analysis system (Bio-Rad, CA USA) and Pierce ECL western blotting substrate (Millipore, Billerica, MA, USA). Signal intensity and protein band quantification were done using the Image Lab 3.0 and Image J software, respectively. β-actin was used as a loading control.

**RNA Isolation and Quantitative Real-Time PCR**

Total RNA from LV tissues and primary cultured cardiomyocytes was isolated using TRIzol reagent (15596018, Ambion). RNA was reverse-transcribed into cDNA using the CFX96 real-time PCR system (Bio-Rad Laboratories, Inc., CA, USA), and SYBR Green Real Time PCR Master Mix Plus (TOYOBO) was used for subsequent real-time quantitative PCR. The 10-μl reactions included 5 μl of SYBR Green dye, 1 μl of cDNA, 0.5 μl of forward primer, 0.5 μl of reverse primer, and 3 μl of ddH_2_O. The following cycling parameters were used: 39 cycles of 95 °C for 30 s, 95 °C for 5 s, and 60 °C for 30 s. The data were analyzed using the 2^-△△Ct^ method, and mRNA levels were normalized to β-actin gene expression. The specific primer sequence information is provided in Supplementary Materials table 1.

**Statistical Analysis**

Graph Pad Prime 8.0 software was used for statistical analyses. All data are presented as the mean±SEM. Unpaired, two-sided Student’s t-test was done to assess differences between two groups. To determine differences between three or more groups, ANOVA followed by Tukey multiple comparison analysis or 2-way ANOVA followed by Bonferroni post hoc test was used. The value of P<0.05 was considered statistically significant. All experiments were repeated at least three times. In the figures, representative images were selected that best match the average data for each experiment.

**References**

1. Shi H, Gao Y, Dong Z, Yang J, Gao R, Li X, et al. GSDMD-Mediated Cardiomyocyte Pyroptosis Promotes Myocardial I/R Injury. Circ Res. 2021;129:383-96.
2. Zheng X, Zhong T, Ma Y, Wan X, Qin A, Yao B, et al. Bnip3 mediates doxorubicin-induced cardiomyocyte pyroptosis via caspase-3/GSDME. Life Sci. 2020;242:117186.

3. Ma H, Guo R, Yu L, Zhang Y, Ren J. Aldehyde dehydrogenase 2 (ALDH2) rescues myocardial ischaemia/reperfusion injury: role of autophagy paradox and toxic aldehyde. Eur Heart J. 2011;32:1025-38.

4. Ren J, Yang L, Zhu L, Xu X, Ceylan AF, Guo W, et al. Akt2 ablation prolongs life span and improves myocardial contractile function with adaptive cardiac remodeling: role of Sirt1-mediated autophagy regulation. Aging Cell. 2017;16:976-87.

5. Li DL, Wang ZV, Ding G, Tan W, Luo X, Criollo A, et al. Doxorubicin Blocks Cardiomyocyte Autophagic Flux by Inhibiting Lysosome Acidification. Circulation. 2016;133:1668-87.

6. Wang Y, Cao X, Liu P, Zeng W, Peng R, Shi Q, et al. KCTD7 mutations impair the trafficking of lysosomal enzymes through CLN5 accumulation to cause neuronal ceroid lipofuscinoses. Sci Adv. 2022;8:m5578.

7. Pan L, Bai P, Weng X, Liu J, Chen Y, Chen S, et al. Legumain Is an Endogenous Modulator of Integrin alphavbeta3 Triggering Vascular Degeneration, Dissection, and Rupture. Circulation. 2022;145:659-74.

8. Xie Y, Gao Y, Gao R, Yang W, Dong Z, Moses RE, et al. The proteasome activator REGgamma accelerates cardiac hypertrophy by declining PP2Acalpha-SOD2 pathway. Cell Death Differ. 2020;27:2952-72.
