# Supplementary material for: Gasdermin D mediates endoplasmic reticulum stress via FAM134B to regulate cardiomyocyte autophagy and apoptosis in doxorubicin-induced cardiotoxicity
Source: Cell Death Dis. 2022 Oct 26;13(10):901. doi: 10.1038/s41419-022-05333-3 (PMC9606128; doi:10.1038/s41419-022-05333-3)
Supplement: Supplementary file 17 — supplementary materials table 1 [file 41419_2022_5333_MOESM17_ESM.docx]

**Supplementary Materials table 1:** Mouse primers used in this study.

| **Gene** | **Forward primers (5’–3’)** | **Reverse primers (5’–3’)** |
| --- | --- | --- |
| ATG-4 | GTCAAGTATGGTTGGGCAGTT | TGTCACCCTCTCCCTCGAAAT |
| ATG-5 | TGTGCTTCGAGATGTGTGGTT | GTCAAATAGCTGACTCTTGGCAA |
| ATG-7 | GTTCGCCCCCTTTAATAGTGC | TGAACTCCAACGTCAAGCGG |
| ATG-10 | GTAGTTACCAAGTGCCGGTTC | AGCTAACGGTCTCCCATCTAAA |
| ATG-12 | AGATTCAGAGGTTGTGCTGC | CAATGAGTCCTTGGATGGTC |
| ATG-16 | CAGAGCAGCTACTAAGCGACT | AAAAGGGGAGATTCGGACAGA |
| Beclin-1 | CCAGAGAAGAATGCTGTACGAAT | TCCATCGAGTCTTATCCTCCAC |
| Bnip-3 | TCCTGGGTAGAACTGCACTTC | GCTGGGCATCCAACAGTATTT |
| LC3B | CGTCCTGGACAAGACCAAGT | AGTGCTGTCCCGAACGTCTC |
| Sqstm1 | ATGTGGAACATGGAGGGAAGA | GGAGTTCACCTGTAGATGGGT |
| Gabarapl1 | GGACCACCCCTTCGAGTATC | CCTCTTATCCAGATCAGGGACC |
| TFEB | CCACCCCAGCCATCAACAC | CAGACAGATACTCCCGAACCTT |
| FAM134B | AAACAGCAGAGTCCTGGCAAG | AGGTAGCTGAGTATGACCCCA |
| β-actin | GGCTGTATTCCCCTCCATCG | CCAGTTGGTAACAATGCCATGT |
